# Inhibition of endoplasmic reticulum stress signaling pathway: A new mechanism of statins to suppress the development of abdominal aortic aneurysm

**DOI:** 10.1371/journal.pone.0174821

**Published:** 2017-04-03

**Authors:** Yuanyuan Li, Gangsheng Lu, Dating Sun, Houjuan Zuo, Dao Wen Wang, Jiangtao Yan

**Affiliations:** Division of Cardiology, Department of Internal Medicine, Tongji Hospital, Tongji Medical College, Huazhong University of Science and Technology, Wuhan, China; Max Delbruck Centrum fur Molekulare Medizin Berlin Buch, GERMANY

## Abstract

**Background:**

Abdominal aortic aneurysm (AAA) is a potentially lethal disease with extremely poor survival rates once the aneurysm ruptures. Statins may exert beneficial effects on the progression of AAA. However, the underlying mechanism is still not known. The purpose of the present study is to investigate whether statin could inhibit AAA formation by inhibiting the endoplasmic reticulum (ER) stress signal pathway.

**Methods:**

A clinically relevant AAA model was induced in Apolipoprotein E-deficient (*ApoE*^−/−^) mice, which were infused with angiotensin II (Ang II) for 28 days. These mice were randomly divided into following 4 groups: saline infusion alone; Ang II infusion alone; Ang II infusion plus Atorvastatin (20mg/kg/d); and Ang II infusion plus Atorvastatin (30mg/kg/d). Besides, another AAA model was induced in C57 mice with extraluminal *CaCl*_2_, which were divided into 3 groups: sham group, *CaCl*_2_-induced AAA group, and *CaCl*_2_-induced AAA plus atorvastatin (20mg/kg/d) group. Then, aortic tissue was excised for further examinations, respectively. *In vitro* studies, Ang II with or without simvastatin treatment were applied to the vascular smooth muscle cells (VSMCS) and Raw 264.7 cells. The ER stress signal pathway, apoptosis and inflammatory response were evaluated by *in vivo* and *in vitro* assays.

**Results:**

We found that higher dose of atorvastatin can effectively suppress the development and progression of AAA induced by Ang II or *CaCl*_2_. Mechanistically, the activation of ER stress and inflammatory response were found involved in Ang II-induced AAA formation. The atorvastatin infusion significantly reduced ER stress signaling proteins, the number of apoptotic cells, and the activation of Caspase12 and Bax in the Ang II-induced *ApoE*^−/−^ mice, compared with mice treated by Ang II alone. Furthermore, proinflammatory cytokines such as IL-6, IL-8, IL-1β were all remarkably inhibited after atorvastatin treatment. *In vitro*, the inhibitory effect of simvastatin on the ER stress signal pathway could be observed in both vascular smooth muscle cells and macrophages, and these inhibitory effects of statin were in a dose-dependent manner. In addition, apoptosis was induced with Ang II treatment. The maximal inhibitory effect of simvastatin on apoptosis was observed at 10 μmol/l.

**Conclusions:**

We conclude that higher dose of statin can effectively suppress the development of AAA, and reduce ER stress, ER stress-associated apoptosis signaling pathways, and inflammatory response. These findings reveal a new mechanism underlying the inhibitory effect of statin on AAA formation/progression.

## Introduction

Abdominal aortic aneurysms (AAAs) occur mostly among men aged 65–85 years in developed countries, and usually remain evidently asymptomatic until the catastrophic event of rupture[1]. It is a complex disease process involving the infiltration of inflammatory cells, the production of reactive oxygen species (ROS), upregulation and activation of degradative proteases, stimulation of apoptosis, degradation of elastin, and transmural inflammation[1]. However, no effective pharmacological strategies are established to suppress the development of AAA or prevent the need for invasive aneurysm repair.

Recent studies demonstrated that 3-hydroxy-3-methyl-glutaryl-CoA reductase (HMG-CoA reductase) inhibitors (or statins) may exert beneficial effects on the progression of AAA. Although some trials reported that the clinical efficacy of statins to reduce AAA progression is limited[2], other observational studies, more experimental researches and meta-analysis data suggested that statin therapy can attenuate AAA growth and it is associated with decreased expansion rates in patients with AAAs[3–5].The recently published European Society of Cardiology guidelines advise the use of statins in patients with AAA, citing data that the use of these agents is associated with threefold reduction in cardiovascular death following elective surgical repair[6]. While most data suggested the mechanism of statin on the AAA is related to the reduction of plasma cholesterol levels[7, 8], it may also involve the pleiotropic effects of statins, including antioxidant, anti-inflammatory, and anti-angiogenic effects[3, 9, 10]. However, the molecular mechanisms of therapeutic effect of statin on AAA is still not well-understood, which need to be further investigated.

Endoplasmic reticulum (ER) stress signaling, often referred to as the unfolded protein response (UPR), can lead to cell pathology and subsequent tissue dysfunction[11]. Previous studies have demonstrated ER stress participates in the pathogenesis of atherosclerosis, heart failure and other cardiovascular diseases by activating C/EBP homologous protein (CHOP)—mediated apoptosis and inflammation[12, 13], in which the pathological progress is similar to the development of AAA. However, whether ER stress is involved in AAA formation have not been studied. Given the protective effects of statin on delaying the progression of AAA, it is reasonable to postulate that statin may target the ER signal pathway and thereby to block AAA progression and exhibit therapeutic effects in clinic. The purpose of our present study is to investigate whether statin could inhibit AAA formation/progression by regulating the ER stress signal pathway. We found that statin can effectively suppress the development of AAA, significantly reduce ER stress signaling and ER stress-associated apoptosis, and inflammatory response.

## Material and methods

### Antibodies and reagents

Angiotensin II (Ang II) and tunicamycin (TM) were obtained from Sigma–Aldrich (St. Louis, MO). Simvastatin was purchased from Cayman Chemical Company (Ann Arbor, MI), and atorvastatin was purchased from Pfizer (New York City, NY, USA). Antibodies against β-actin(BM0627), IL-6 (BA4339-2), IL-8(BA0996), IL-1β(BA2782), MCP-1 (BA1843-2), CD68(BA3638) were from Boster Biological Technology(Pleasanton, CA). Protein kinase RNA (PKR)-like ER kinase (PERK)(sc-32577) (H-300), phosphorylated protein kinase RNA (PKR)-like ER kinase (p-PERK) (sc-32577), inositol-requiring protein-1α (IRE1α) (sc-20790), glucose-regulated protein 78 (GRP78) (sc-13968), ER marker KDEL(sc-58774), phosphorylated α-subunit of eukaryotic translation initiation factor-2 (p-EIF2α)(FL-315), and Caspase12(sc-21747) were purchased from Santa Cruz Biotechnology (Santa Cruz, CA). Activating transcription factor-6α (ATF6α)(24169-1-AP) was from Proteintech Group, Inc (Chicago, USA). Antibody for CHOP(L63F7) was purchased Cell Signaling Technology (Beverly, MA). Dulbecco's modified Eagle's medium (DMEM) and fetal bovine serum (FBS) were obtained from Life Technologies (Grand Island, NY). Horseradish peroxidase-conjugated secondary antibodies were from Thermo Fisher Scientific (Rockford, IL). All other chemicals were purchased from Sigma-Aldrich (St. Louis, MO) unless otherwise indicated.

### Animals

All animal work has been conducted according to relevant national and international guidelines. Animals were anesthetized by injecting 1% pentobarbital before animal work. About 7 mice died unexpectedly before the experimental endpoint. Mice were housed in temperature-controlled cages with a 12-h light-dark cycle and given free access to water and food. This research is approved by animal care facility of Tongji Medical College. C57BL/6 mice were obtained from Animal Biosafety Level 3 Laboratory of Wuhan University. *ApoE*^−/−^ (C57BL/6 background) mice supplied by Charles River Laboratories (Massachusetts, USA) were housed at the animal care facility of Tongji Medical College under specific pathogen-free conditions, and were fed with normal diet. All animal experimental protocols followed the Guide for the Care and Use of Laboratory Animals published by the US National Institutes of Health (NIH Publication No. 85–23, revised 1996) and the Public Health Service (PHS) Policy on Humane Care and Use of Laboratory Animals. The animal studies were approved by the Institutional Animal Research Committee of Tongji Medical College.

### Establishment of the Ang II-induced mouse AAA model

Forty 8-week-old *ApoE*^−/−^ mice were randomly divided into the following 4 groups: saline infusion alone (n = 10); Ang II infusion alone (n = 10); Ang II infusion plus Atorvastatin (20mg/kg/d, n = 10); and Ang II infusion plus Atorvastatin (30mg/kg/d, n = 10). Each atorvastatin was suspended in 0.9% normal saline and administrated by gavage once a day. Osmotic minipumps (Model 2004, Durect Corporation, Cupertino, CA, USA) were implanted into the mice to continuously deliver Ang II (Sigma-Aldrich, St. Louis, MO) subcutaneously at a dose of 1,000 ng/kg/min or saline vehicle for 28 days, as previously described[14]. The next day after implanting, mice were treated with each drug by gavage for 28 days.

### Establishment of the *CaCl*_2_-induced mouse AAA model

Briefly, forty C57/BL6 mice (8 weeks) which were chosen to undergo this treatment were randomly divided into 3 groups: *CaCl*_2_-induced AAA, sham group, *CaCl*_2_-induced AAA plus atorvastatin (20mg/kg/d). Each mouse was anesthetized by injecting 1% pentobarbital before the abdominal aortic region located distal to the renal arteries and proximal to the iliac bifurcation were surgically exposed. A gauze pre-soaked in *CaCl*_2_ (0.5M) was then directly applied to the adventitia of the infra-renal abdominal aorta for a period of time (about 15 minutes) and then removed. Alternatively, a swab soaked with *CaCl*_2_ solution was employed to “paint” the abdominal aorta for a period of time. The abdominal cavity was then washed with warm sterile saline before the abdominal cavity was closed. Control animals were exposed to a sham procedure performed as described above except that sterile saline solution was used instead of *CaCl*_2_ solution. One week after implanting, mice were treated with each drug by gavage for seven weeks. Eight weeks after the *CaCl*_2_ treatment, mice were randomly chosen and sacrificed. The diameters of their abdominal aortas were measured under a microscope, and the vessels were collected for histological analysis.

### Quantification analysis of AAA

Animals were sacrificed at the end of the interventions. For AAA quantification, the maximum width of the abdominal aorta was measured in each mouse by Image Pro Plus software (Media Cybernetics, Bethesda, MD). AAA in mice was defined as a 50% or greater increase in the external width of the suprarenal aorta compared with aortas from the controls[11]. Grading system: Type I, dilated lumen without thrombus; Type II, remodeled tissue with little thrombus; Type III, a pronounced bulbous form of Type II with thrombus; Type IV, multiple, often overlapping aneurysms containing thrombus[15].

### Histological analysis

Abdominal aortic tissues were harvested, fixed in 4% paraformaldehyde in PBS, and embedded in paraffin for histological analysis. Some aortic tissues were obtained and kept frozen in liquid nitrogen immediately, and then stored at −80°C for western blot. Three micron cross-sections were prepared and subsequently stained with hematoxylin and eosin, Verhoeff-Van Gieson (EVG) respectively. Immunohistochemical staining was performed according to the manufacture's description (Zsbio, Beijing, China). The following antibodies were applied: IL-6, IL-8, IL-1β, α-smooth muscle actin (α-SMA), macrophage marker protein CD68, and monocyte chemotactic protein-1 (MCP-1) from Santa Cruz Biotechnologies (California, USA). Negative controls have been done to validate the specificity of each immunostaining. The immunohistochemical staining results were quantified by Image Pro Plus software.

### Terminal deoxynucleotidyl transfer-mediated dUTP nick end-labeling (TUNEL) staining

TUNEL staining was conducted using POD, an in-situ cell death detection kit (Roche, Germany), according to the manufacturer’s instructions. Following deparaffinization and rehydration, the sections were treated with 10 mM protease K for 15 min. The slides were immersed in a TUNEL reaction mixture for 60 min at 37°C in a humidified atmosphere in the dark. The slides were incubated in Converter-POD for 30 min to show blue nuclear staining and then analyzed via optical microscopy. The TUNEL index (%) was computed by dividing the ratio of the number of TUNEL-positive cells by the total number of cells.

### Cell culture and treatment

Vascular smooth muscle cells, a subclone of the original clonal cell line derived from mouse thoracic aorta, were obtained from American Type Culture Collection (VA, USA). RAW 264.7 cells, a mouse macrophage cell line, were purchased from American Type Culture Collection (VA, USA). Both cells were cultured in Dulbecco’s modified Eagle’s medium supplemented with 10% fetal bovine serum and penicillin-streptomycin (100 IU/ml) in a humidified atmosphere of 95% air and 5% CO_2_ at 37°C. Cells were incubated with different concentration of Ang II for 24 h (1μmol/l, 10μmol/l, 20 μmol/l, 50 μmol/l) respectively. If needed, 0.1 μmol/l simvastatin, 1 μmol/l simvastatin, or 10 μmol/l simvastatin was added 1 h before Ang II treatment (20 μmol/l).

### Annexin V-FITC/PI double staining assay

Cultured cells were harvested with trypsin and resuspended in binding buffer. Five microliters Annexin V-fluorescein isothiocyanate and 5 ml propidium iodide (50 mg/ml) were added according to the manufacturer’s protocol (KeyGEN, Nanjing, China). Cells were then analyzed with a FACStar-Plus flow cytometer (Becton Dickinson, Franklin Lakes, NJ).

### Western blot analysis

Tissue samples and cell lysates were extracted according to the manufacturer's protocol (Boster Biological). Protein concentrations were determined using the BCA assay. Western blots were performed using various antibodies. Briefly, equal amounts of extracts were separated by 10% SDS-PAGE, transferred onto PVDF membranes. After blocking with 5% (w/v) bovine serum albumin (BSA), the membranes were incubated with appropriate primary antibodies at 4°C overnight, followed by incubation with peroxidase- conjugated secondary antibodies at room temperature for 2 h. The blots were visualized with enhanced chemiluminescence detection reagent. β-actin was used as internal controls. Bands were quantified by densitometry using Quantity One software (Bio-Rad, Hercules, CA).

### Statistical analysis

All data are presented as mean ± SEM. After confirming the normal distribution using the Kolmogorov-Smirnov test, statistical differences were evaluated by ANOVA followed by Turkey comparison test. Chi-Square test was applied to comparisons of AAA incidence and aortic rupture rate. Statistical significance was evaluated with GraphPad Prism4. *P* < 0.05 was accepted as statistically significant.

## Results

### Higher dose of atorvastatin attenuates Ang II-induced AAA formation in *ApoE*^−/−^ mice

**Fig 1:**
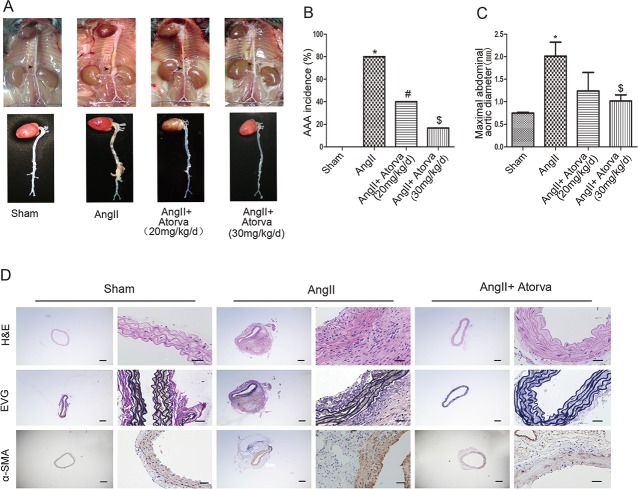
Atorvastatin attenuates Ang II-induced AAA formation in *ApoE*^−/−^ mice. (A) Gross morphology of abdominal aorta with indicated interventions. The arrow indicates a typical AAA or not. (B) The incidence of Ang II-induced AAA. (C) Maximal abdominal aortic diameter. (D) Representative staining with HE, EVG, and α- SMA in suprarenal aortas of *ApoE*^−/−^ mice. Scale bar: in each group, left 200μm and right 50μm. All results present 5–10 mice. *P < 0.05 versus control. #P<0.05 versus Ang II infusion alone.

Ang II-induced AAA is a well-characterized mouse model[11]. After 4-week Ang II infusion, obvious abdominal aortic aneurysm was developed in *ApoE*^−/−^ mice (Fig 1A). Consistent with previous reports[16], the incidence of AAA induced by Ang II infusion was 80% (Fig 1B) in *ApoE*^−/−^ mice accompanied by markedly increased maximal abdominal aortic diameter (Fig 1C). Histological analysis showed that Ang II-treated *ApoE*^−/−^ mice exhibited a significantly increased size of the aortic lumen and wall thickness. More importantly, the elastic lamina was severely disrupted and degraded in Ang II-treated *ApoE*^−/−^ mice (Fig 1D). There was no abdominal aortic aneurysm formation in saline-infused *ApoE*^−/−^ mice and aortas in control mice were intact and normal.

In contrast, only 17% Ang II-infused *ApoE*^−/−^ mice with intragastric atorvastatin (30mg/kg/d) infusion developed AAA and 40% mice with intragastric atorvastatin (20mg/kg/d) infusion had AAA formation (Fig 1B). The maximal abdominal aortic diameter was markedly reduced in mice with intragastric atorvastatin (30mg/kg/d) infusion, compared with the mice with Ang II infusion alone or with Ang II and intragastric atorvastatin (20mg/kg/d) infusion (Fig 1C). Meanwhile, atorvastatin (30mg/kg/d) infusion remarkablely reduced aortic rupture rate and systolic blood pressure compared with Ang II infusion alone (S1A and S1C Fig). Histologically, aortic wall thickness/aortic expansion or elastic lamina degradation in mice with intragastric atorvastatin infusion (30mg/kg/d) were dramatically improved (Fig 1D). According to the grading system, 16.7% mice with Ang II infusion alone were Type I AAAs and 66.7% of Ang II infused mice were Type III AAAs; 40% mice with Ang II infusion plus atorvastatin (20mg/kg/d) presented Type III aneurysms while 14.3% mice with Ang II infusion plus atorvastatin (30mg/kg/d) presented Type II aneurysms (S1 Table). Overall, these results suggest that high-dose atorvastatin infusion protects against Ang II-induced AAA in *ApoE*^−/−^ mice.

### Atorvastatin suppresses *CaCl*_2_-induced AAA formation in C57/BL6 mice

**Fig 2:**
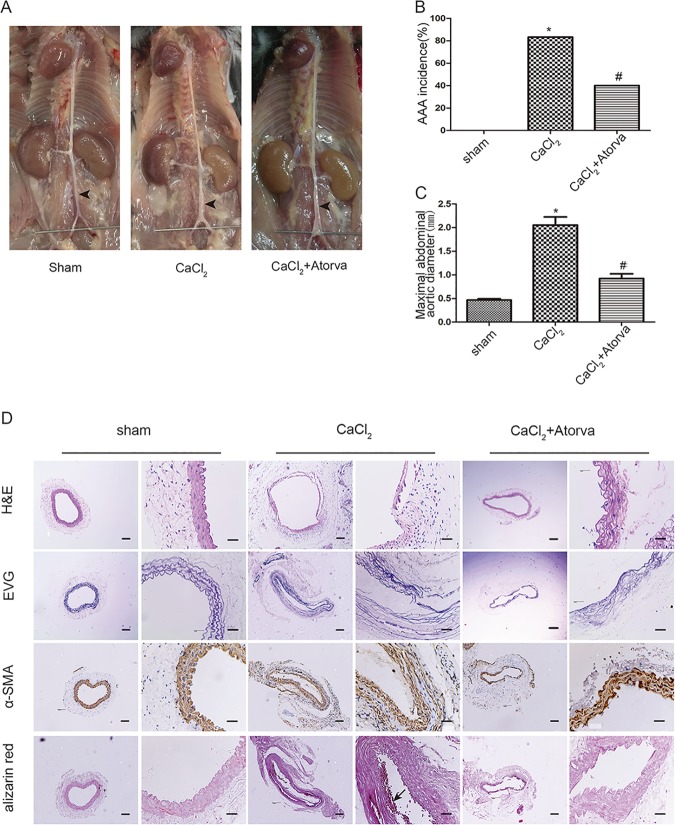
Atorvastatin prevents the progression of *CaCl*_2_-induced AAA in C57/BL6 mice. (A) Gross morphology of abdominal aorta with indicated interventions. The arrow indicates a typical AAA or not. (B) The incidence of *CaCl*_2_-induced AAA. (C) Maximal abdominal aortic diameter. (D) Representative staining with HE, EVG, α- SMA and Alizarin red in infrarenal aortas of *CaCl*_2_ mice. Scale bar: in each group, left 200μm and right 50μm. All results present 5–10 mice. *P < 0.05 versus control. #P<0.05 versus *CaCl*_2_ alone.

In the next step, we used another well-established AAA mouse model, *CaCl*_2_-induced AAA formation to further demonstrate the protective effect of statin against the development of AAA. After 8-week *CaCl*_2_ treatment, the incidence of AAA was 83% (Fig 2A and 2B). Compared with sham-operated mice, the maximal abdominal aortic diameter was evidently increased in the *CaCl*_2_-treated group (Fig 2C). Meanwhile, it was also found significant aortic dilation, serious destruction of elastic lamina, disordered arrangement of smooth muscle cells, and obvious calcium deposition throughout the media of *CaCl*_2_-treated mice (Fig 2D). Otherwise sham-operated mice were almost normal (Fig 2D).

However, the incidence of AAA in mice with *CaCl*_2_ injury plus atorvastatin (20mg/kg/d) infusion was reduced to 40% (Fig 2B). And the maximal abdominal aortic diameter was markedly decreased and less calcium deposition occurred throughout the media (Fig 2C and 2D). Moreover, atorvastatin protected these mice from disruption of elastic lamellae and made the smooth muscle cells arrangements more regular than mice with *CaCl*_2_ injury. These results suggest that atorvastatin could also reduce *CaCl*_2_-induced AAA formation *in vivo*.

### Atorvastatin reduces ER Stress signaling and apoptosis in Ang II-induced AAA formation *in vivo*

**Fig 3:**
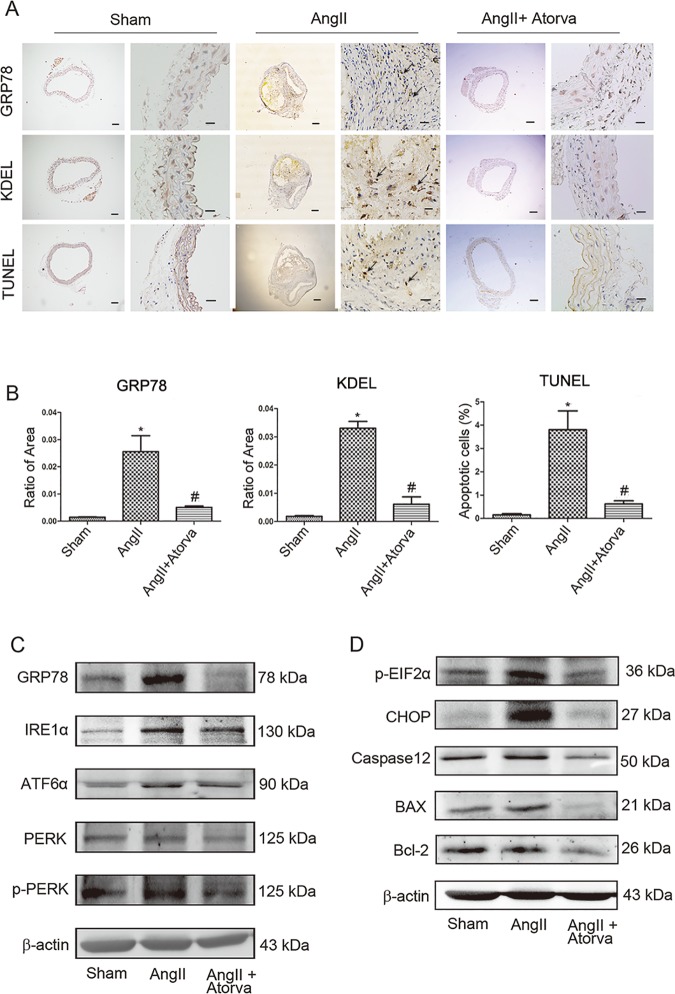
Atorvastatin decreases ER Stress signaling and apoptosis in Ang II-induced AAA in vivo. (A) Representative immuno-histochemical staining image of GRP78, KDEL, TUNEL. Scale bar: in each group, left 200μm and right 50μm. (B) Ratio of positive staining area of GRP78 and KDEL; Percentage of apoptotic cells. N is 5 in each group (C-D) Western blots show PERK-p-EIF2α-CHOP and apoptosis pathway. N = 3. *P < 0.05 versus control. #P<0.05 versus Ang II infusion alone.

To dissect the molecular mechanism underlying the protective effect of statin against AAA development, we first determined whether ER stress signaling contributes to Ang II-induced AAA formation. Immunohistochemical results showed that the expression level of GRP78 and KDEL, two ER Stress marker proteins[17], were markedly elevated in abdominal aortic tissues compared with control mice (Fig 3A and 3B). However, the expression of these two proteins was attenuated in atorvastatin infused mice. Further, western blots analysis indicated that expression of ER stress molecules including GRP78, ATF6α, IRE1α, p-PERK, p-EIF2α and CHOP were all significantly increased in Ang II-induced *ApoE*^−/−^ mice (Fig 3C and 3D, S3 Fig). In contrast, after atorvastatin infusion, the Ang II-induced *ApoE*^−/−^ mice displayed reduced expression of GRP78, ATF6α, IRE1α, p-PERK, p-EIF2α and CHOP proteins.

Next, we tested whether ER stress induces apoptosis in these mice, we used TUNEL staining to quantify the number of vascular smooth cells undergoing apoptosis. Compared with saline-treated group, Ang II-treated mice exhibited an increase in the percentage of TUNEL-positive cells (Fig 3A and 3B). And western blots data showed that the expression of Caspase12 and the ratio of Bax/Bcl-2 were significantly higher in the Ang II-treated mice than in the saline-treated mice (Fig 3C and 3D, S3 Fig). However, it was observed that atorvastatin infused mice displayed fewer TUNEL-positive cells, reduced activation of Caspase12 and lower ratio of Bax/Bcl-2 compared with the Ang II alone -treated mice.

Taken together, these data suggest that the signals of ER stress and apoptosis are both significantly activated in Ang II-induced *ApoE*^−/−^ mice, and these changes were attenuated by treatment with atorvastatin. Our results support the notion that atorvastatin can protect *ApoE*^−/−^ mice treated with Ang II from ER stress and ER stress-associated apoptosis.

### Atorvastatin reduces inflammatory response in the Ang II-induced *ApoE*^−/−^ mice

**Fig 4:**
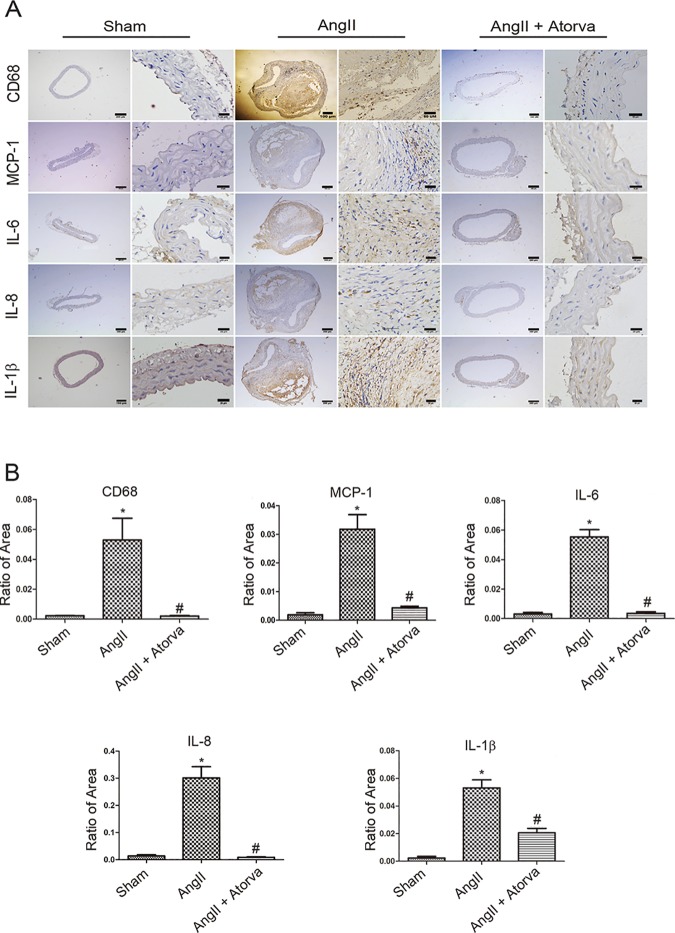
Atorvastatin inhibits the infiltration of inflammatory cells and proinflammatory cytokines in Ang II-induced *ApoE*^−/−^ mice. (A) Representative immuno-histochemical staining image of macrophage marker protein CD68 and inflammatory cytokines IL-6, IL-8, IL-1β, MCP-1 in these treated mice. Scale bar: in each group, left 200μm and right 50μm. (B-F) Ratio of positive staining area of CD68, IL-6, IL-8, IL-1β, MCP-1. N is 5 in each group. *P < 0.05 versus control. #P<0.05 vs. Ang II alone.

We detected the infiltration of inflammatory cells and cytokines with immunohistochemical assay. The expression levels of monocyte chemotactic protein-1 (MCP-1) and macrophage marker protein CD68 in Ang II-induced *ApoE*^−/−^ mice were obviously higher than that of saline-treated mice (Fig 4). And proinflammatory cytokines, such as IL-6, IL-8 and IL-1β, were all up-regulated after Ang II infusion. However, they were remarkably reduced after atorvastatin treatment (Fig 4). Taken together, these data suggest that inflammatory responses are significantly activated in Ang II-induced *ApoE*^−/−^ mice and atorvastatin reduces inflammatory response in these mice.

### Angiotensin II distinctly induced ER stress-associated apoptotic signaling pathways *in vitro*

We next performed experiments in VSMCS and RAW 264.7 cells to further elucidate the mechanism involved, since VSMCS are the major cell type in Ang II-induced AAA in mice[18] and early macrophage infiltration is a key feature in AAA pathogenesis[19].

**Fig 5:**
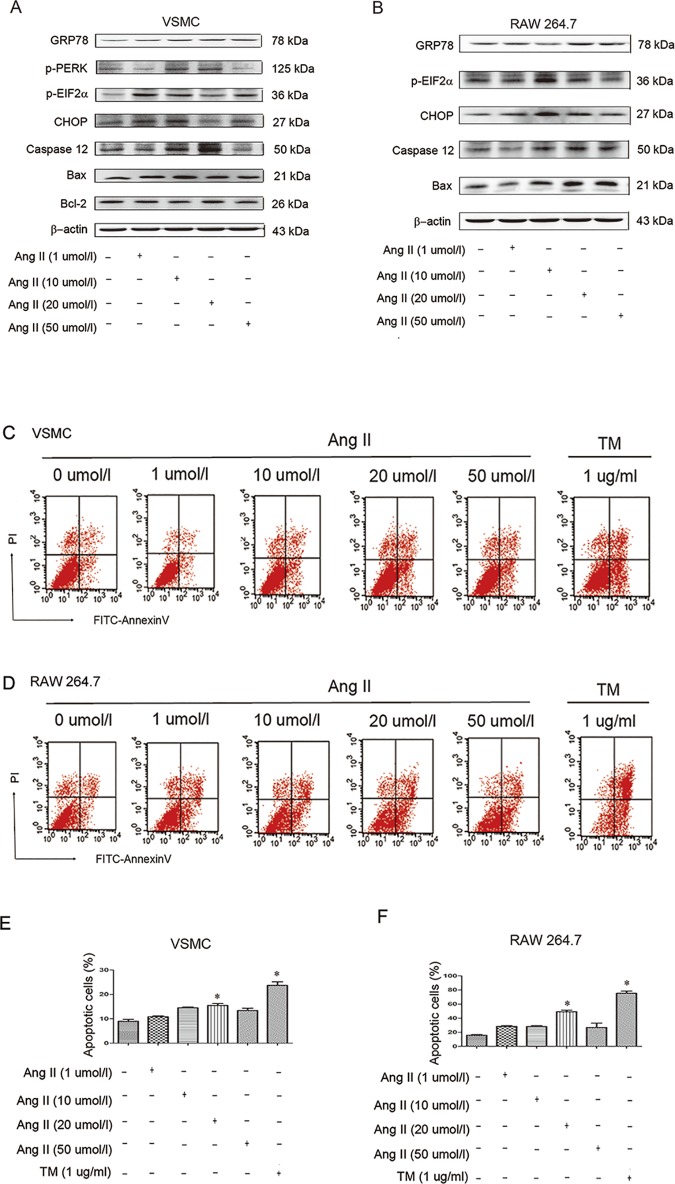
Angiotensin II induces ER stress-associated apoptotic signaling pathways in vitro. (A) Representative immunoblots of ER stress pathway protein induced by concentration gradient of Ang II (1–50μmol/l) in vascular smooth muscle cells. (B) Representative immunoblots of ER stress pathway protein induced by concentration gradient of Ang II (1–50μmol/l) in RAW 264.7 cells. (C) Annexin V-FITC/PI double staining assay was performed to examine apoptosis of vascular smooth muscle cells treated with Ang II (1–50μmol/l) and TM (1ug/ml). (D) Annexin V-FITC/PI double staining assay was performed to examine apoptosis of RAW 264.7 cells treated with Ang II (1–50μmol/l) and TM (1ug/ml). (E) quantitative estimates of apoptotic vascular smooth muscle cells in total cells. (F) quantitative estimates of apoptotic RAW 264.7 cells in total cells. N = 3. *P < 0.05 versus control.

Firstly, we induced VSMCS with different concentrations of Ang II (1–50μmol/l). Western blots results showed that the levels of ER stress and apoptosis associated proteins could be increased by Ang II treatment. However, these proteins exhibited different responses to different concentrations of Ang II treatment (Fig 5A and S2A Fig). For example, it was observed that the GRP78, p-PERK, Caspase12 exhibited concentration (1–20μmol/l) dependence. The CHOP protein level and the Bax/Bcl-2 ratio were highest at the concentration of 10μmol/l, while the p-EIF2α was highest at 1μmol/l (Fig 5A and S2A Fig).

Then we treated RAW 264.7 cells with different concentrations of Ang II. The expression of ER stress and apoptosis associated proteins has also been analyzed. Similarly to the VSMCS, the different concentrations of Ang II markedly increased expression levels of these proteins. Since the cell background of RAW 264.7 cells is different from VSMCS, the proteins involved and their expression levels were different. Notably, the GRP78, Caspase 12 and Bax were showed to be Ang II concentration (1–20μmol/l) dependent. Other proteins, such as CHOP and p-EIF2α were activated most obviously at 10μmol/l (Fig 5B and S2C Fig).

Additionally, Annexin V-FITC/PI double staining assay in both VSMCS and RAW 264.7 cells suggested that cell apoptosis also exhibited Ang II concentration (1–20μmol/l) dependence and ER stress inducer TM (1ug/ml) triggered obvious cell apoptosis (Fig 5C–5F).

In conclusion, these data show that Ang II can markedly induce ER stress and cell apoptosis *in vitro*.

### Simvastatin inhibits the ER stress-associated apoptosis *in vitro*

**Fig 6:**
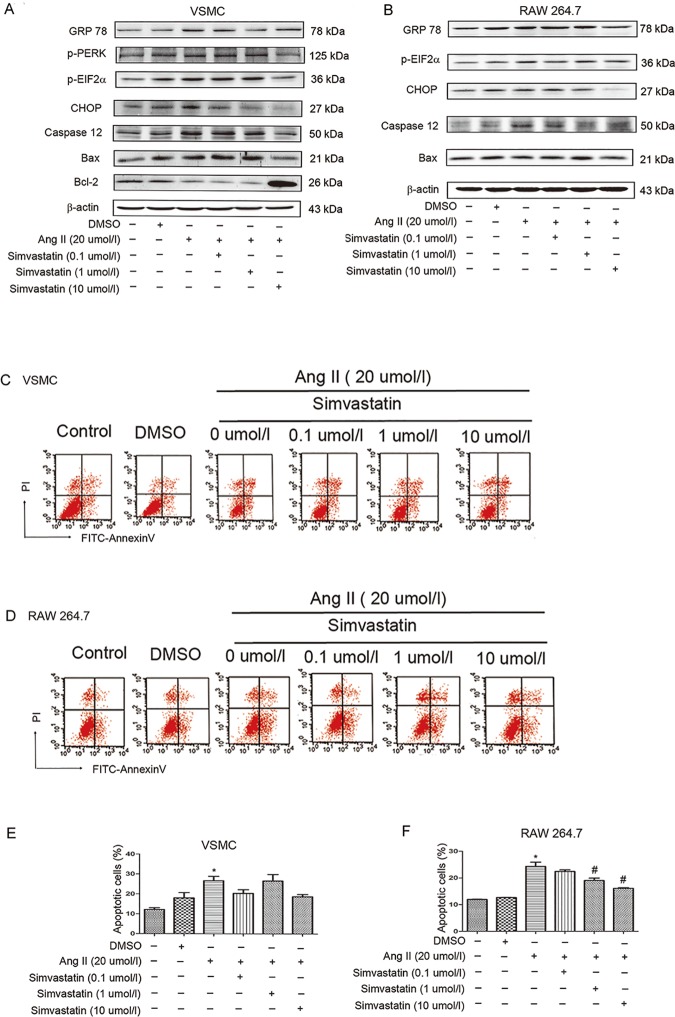
Simvastain inhibits ER stress signaling pathways and apoptosis induced by Ang II. (A) Representative immunoblots of ER stress pathway protein induced by Ang II (20μmol/l) with pretreatment of simvastatin (0–10μmol/l) in vascular smooth muscle cells. (B) Representative immunoblots of ER stress pathway protein induced by Ang II (20μmol/l) with pretreatment of simvastatin (0–10μmol/l) in RAW 264.7 cells. (C) Annexin V-FITC/PI double staining assay was performed to examine apoptosis of vascular smooth muscle cells with indicated interventions. (D) Annexin V-FITC/PI double staining assay was performed to examine apoptosis of RAW 264.7 cells with indicated interventions. (E) quantitative estimates of apoptotic vascular smooth muscle cells in total cells. (F) quantitative estimates of apoptotic RAW 264.7 cells in total cells. N = 3. *P < 0.05 versus control. #P<0.05 vs. Ang II (20μmol/l) plus simvastatin (0μmol/l).

In order to determine whether simvastatin blocks ER stress, we treated VSMCS with different concentrations of simvastatin (0.1–10μmol/l) before Ang II (20μmol/l) induction. As shown in the Fig 6A and S2B Fig, the inhibitory effect of simvastatin on ER stress was observed in a dose-dependent response. The GRP78 protein expression was reduced within the concentration range between 0 μmol/l and 1μmol/l. While, the inhibitory effects of simvastatin upon p-PERK, CHOP, and Caspase 12 were between 0.1 and 10 μmol/l. The expression of p-EIF2α and the ratio of Bax/Bcl-2 were suppressed most potently by simvastatin at the concentration of 10 μmol/l (Fig 6A and S2B Fig).

Then we confirmed these observations by using RAW 264.7 cells as our in vitro model. RAW 264.7 cells were treated with different concentrations of simvastatin (0.1–10μmol/l) before Ang II (20μmol/l) induction, and similar effects were observed (Fig 6B and S2D Fig). We found a dose-dependent inhibition of CHOP, Caspase 12, and Bax. The expression of GRP78 and p-EIF2α proteins were most significantly reduced at the concentration of simvastatin at 10 μmol/l.

In addition, Annexin V-FITC/PI double staining assay in both VSMCS and RAW 264.7 cells showed that the percentage of apoptotic cells was reduced to the lowest level when the concentration of simvastatin was 10 μmol/l (Fig 6C–6F).

Together, these data suggest that high-dose simvastatin significantly suppressed ER stress-associated apoptotic signaling pathways.

## Discussion

Pharmaceutical management to stabilize and prevent AAA progression is a big challenge in clinical practice and is of great interest to cardiovascular researchers. It is thought that statins may be relevant to pathogenesis of AAA due to its pleiotropic effect on inflammation and effect on atherosclerosis. In our present study, we demonstrated that high-dose atorvastatin can effectively suppressed the development and progression of AAA induced by Ang II or *CaCl*_2_. Mechanistically, atorvastatin could reduce ER stress and inflammatory response involved in Ang II-induced AAA formation *in vivo*, and simvastatin can inhibit the ER stress-associated apoptosis signaling pathways *in vitro*. Notable, the involvement of ER stress provides novel mechanism for explaining the function of statins in protecting AAA formation.

In our study, we clearly demonstrated the effect of statin on AAA progression. However, previous studies [20] suggested that there is an apparent paradox in conclusions for an effect of statins on AAA progression, particularly from the clinical studies. There are several possibilities to explain. Firstly, most of clinical studies are prospective design rather than randomized clinical and limited number of patients were involved with a relatively small sample size. Secondly, these studies were conducted with a small cohort of patients with small AAAs excluding other AAA type. Thus, the clinical effects of statin on AAA progression remains to be controversial. It is worthy of noting that strong evidence from a number of independent studies demonstrated that statins may influence aneurysm growth rate, presumably via these pleiotropic effects [21],[22]. Our experimental data may provide a solid rationale to support further clinical studies of statin in AAA by improving the clinical study design for example stratifying different subtypes of AAA for statin treatment.

Angiotensin model is a traditional AAA model with dose of 1000ng/kg/min for 28 days[11]. Previous studies have demonstrated that infusion of Ang II into apolipoprotein E–deficient (apoE-/-) or fat-fed, LDL receptor-/- mice leads to reproducible formation of AAAs, particularly in male mice[23]. Results from their studies demonstrate that medial accumulation of macrophages and dissection are early events in Ang II-induced AAA. One of our most important findings is the activation of ER stress signal pathway in the AAA formation in Ang II-induced *ApoE*^−/−^ mice. This conclusion is supported by our in vitro and in vivo assays. As we known, ER stress signaling, often referred to the unfolded protein response (UPR), is triggered by 3 upstream proteins, the protein kinase and RNA processing enzyme IRE1, the protein kinase PERK, and the transcription factor ATF6[11, 17, 24]. ER stress signaling is mediated through IRE1α and then activated IRE1α promotes the expression of XBP1[25]; PERK, through phospho-eIF2α–mediated upregulation of ATF4[26, 27], also leads to the induction of CHOP[28]; the activated ATF6 can act independently or synergistically with XBP1s for induction of UPR target genes[11]. Although accumulating evidence demonstrates the ER stress is involved in a variety of cardiovascular diseases and it has been thought to be a key player in development of atherosclerosis and diabetes[28–32], little is known about the role of ER stress in AAA formation. Previous study only reported an enhanced expression of CHOP and KDEL (ER stress markers) in aorta tissue with AAA[33, 34], which provides a possible link between ER stress and AAA formation. In our study, first, we showed that essential players of ER stress signaling, including GRP78, ATF6α, IRE1α, p-PERK, p-EIF2α, and CHOP, were all significantly increased in Ang II-induced *ApoE*^−/−^ mice, which demonstrated the activation of ER stress. Second, our data found that the Ang II-treated mice exhibited an increase in the percentage of vascular smooth cells undergoing apoptosis, and ER stress-associated apoptosis signals (eg: CHOP, Caspase 12, Bax) were also significantly higher in the Ang II-treated mice. In fact, consistent with our observations, previous studies have shown that chronic or severe ER stress activates the UPR leading to apoptotic death[11], and PERK-p-EIF2α-CHOP signaling is a primary determinant for apoptosis[35]. Further, our *in vitro* study showed the activation of ER stress signal in the Ang II treated VSMCS and macrophages, which were both key cell types involved in the development of AAA and ER stress[1, 28, 36]. Thus, these findings demonstrated that PERK-p-EIF2α-CHOP and its related apoptosis pathway were activated in our *in vivo* and *in vitro* models, and our study revealed an important role of ER stress in the pathogenesis of AAA formation. In addition, previous study and our supplementary data suggested that the significantly increased levels of ER stress associated protein in Ang II-treated mice that develop AAA are attributed to the cellular proliferation and intense infiltration of inflammatory cells in the aortic wall [37].

Another important finding of this study is a novel mechanism underlying the protective effect of statin against AAA formation. The statins can reduce or inhibit proteolysis and inflammation of the aneurysmal wall[2, 3, 16], and it has been suggested that this class of drugs may possess inhibitory effects on aneurysm growth[38–40]. The mechanism by which statins affect the progression of AAAs is likely due to their pleiotropic effects, and is thought to be independent of serum cholesterol levels[7, 8] as our data shown in S1B Fig. The function to regulate leukocyte recruitment and immuno-inflammatory responses, platelet activation, oxidative stress, proteolysis, extracellular matrix breakdown, and upregulation of Heme oxygenase-1 are possible mechanisms of statin on inhibiting the development of AAA[3, 9, 10, 41]. However, the underlying mechanism still needs to be investigated. The ER stress involved in the mechanism of statin has been reported in some diseases. It was reported that atorvastatin treatment decreases apoptosis of myocardial cells by down-regulating GRP78, caspase-12 and CHOP expression in myocardial cells after myocardial infarction, suggesting a possible mechanism of ER stress by atorvastatin[42]. Whether ER stress plays a role in statin-mediated protective effect on the AAA remains unknown. Our study firstly showed that statins could improve AAA progression in general. Our data further found that stains could inhibit apoptosis and inflammation of aorta via down-regulating PERK-p-EIF2α-CHOP associated ER stress signaling pathway. We believe it is one of mechanisms how statins could affect AAA progression, although more studies are needed for other possible mechanisms. For our best knowledge, inhibition of ER stress-associated apoptosis and signaling pathways by statins is a novel mechanism involved in the AAA formation. And our further experiments in VSMCs and RAW 264.7 cells suggest that this effect is not via AT1 receptor (S5 Fig and S6 Fig). *In vivo* study, our data showed that these key proteins involved in ER stress signal pathway proteins were significantly inhibited after atorvastatin infusion; fewer apoptosis cells were observed and the activation of Caspase12, the Bax/Bcl-2 ratio were also reduced in the atorvastatin infused mice. Furthermore, our *in vitro* study showed high-dose simvastatin significantly suppressed ER stress-associated apoptotic signaling pathways. The inhibitory effect of simvastatin upon the ER stress signal pathway could be observed both in the VSMCS and macrophages, and some effects were exhibited dose-dependent responses. In summary, our study showed that, statins have inhibitory effects on the ER stress-associated apoptosis signaling pathways, which attenuate the development of AAA. This provide new mechanistic insights into protective effects of statin against AAA formation.

In addition, our data also showed the expression levels of MCP-1, macrophage marker protein CD68, and proinflammatory cytokines were all up-regulated after Ang II infusion. Interestingly, Atorvastatin treatment reduces inflammatory response in the Ang II-induced *ApoE*^−/−^ mice which are consistent with the data reported before[2, 3].

In summary, we demonstrate the function of statins in preventing the development and progression of AAA and provide evidence to support a crucial role of ER stress in the formation of AAA. More importantly, our study uncovers a novel mechanism underlying the therapeutic effects of statins in AAA through the reduction of ER stress, ER stress-associated apoptosis, and inflammatory response.

## Supporting information

S1 Fig Aortic rupture rate data, Lipid levels and systolic blood pressure. (A) Aortic rupture rate of the 3 groups respectively are: 0.4, 0.375, 0.125. N = 5–10. (B) Lipid levels: there was no statistical significance of lipid levels among groups. N = 3–5. (C) systolic blood pressure: N = 5–6. *P < 0.05 versus control. #P<0.05 versus Ang II infusion alone.
(TIF)

S2 Fig Quantitation of Western blotting. (A) Quantitation of Fig 5A. (B) Quantitation of Fig 6A. (C) Quantitation of Fig 5B. (D) Quantitation of Fig 6B. N = 3. *P < 0.05 versus control. #P<0.05 versus Ang II+ Simva(0umol/L).
(TIF)

S3 Fig Quantitation of Western blotting. (A) Quantitation of Fig 3C. (B) Quantitation of Fig 3D. N = 3. *P < 0.05 versus control. #P<0.05 versus Ang II infusion alone.
(TIF)

S4 Fig Western blotting of *ApoE*^−/−^ mice wih AngII alone/plus atorvastatin within 1–5 days. (A) Western blots show PERK-p-EIF2α-CHOP apoptosis pathway. (B) Quantitation of Fig A. N = 3. It indicates no significant difference among groups.
(TIF)

S5 Fig Anti-apoptosis effects of Ang II on VSMCs may not be through AT1 receptor antagonist. (A) Representative immunoblots of ER stress pathway protein induced by Ang II (20μmol/l) with pretreatment of simvastatin (10μmol/l), Lorsatan(10μmol/l) or both in vascular smooth muscle cells. (B) Quantitation of Fig A. *P < 0.05 versus control. #P<0.05 versus Ang II. Data are shown from three independent experiments.
(TIF)

S6 Fig Anti-apoptosis effects of Ang II on RAW 264.7 may also not be through AT1 receptor antagonist. (A) Representative immunoblots of ER stress pathway protein induced by Ang II (20μmol/l) with pretreatment of simvastatin (10μmol/l), Lorsatan(10μmol/l) or both. (B) Quantitation of Fig A. *P < 0.05 versus control. #P<0.05 versus Ang II. Data are shown from three independent experiments.
(TIF)

S7 Fig Negative controls of immunostaining: Negative controls of immunostaining of GRP78, KDEL, TUNEL, IL-6, IL-8, IL-1β, CD68, MCP-1.
(TIF)

S1 Table Evaluation of Ang II-induced AAA model with Grading system: 16.7% mice with Ang II infusion alone were Type I AAAs and 66.7% of Ang II infused mice were Type III AAAs; 40% mice with Ang II infusion plus atorvastatin (20mg/kg/d) presented Type III aneurysms while 14.3% mice with Ang II infusion plus atorvastatin (30mg/kg/d) presented Type II aneurysms.
(XLSX)

S1 File Supplement Data file: Pressure data of Ang II-induced AAA model and individual data points corresponding to each statistical graph.
(ZIP)
