# Supplementary figures and images for: Inhibition of endoplasmic reticulum stress signaling pathway: A new mechanism of statins to suppress the development of abdominal aortic aneurysm
Source: PLoS One. 2017 Apr 3;12(4):e0174821. doi: 10.1371/journal.pone.0174821 (PMC5378361; doi:10.1371/journal.pone.0174821)

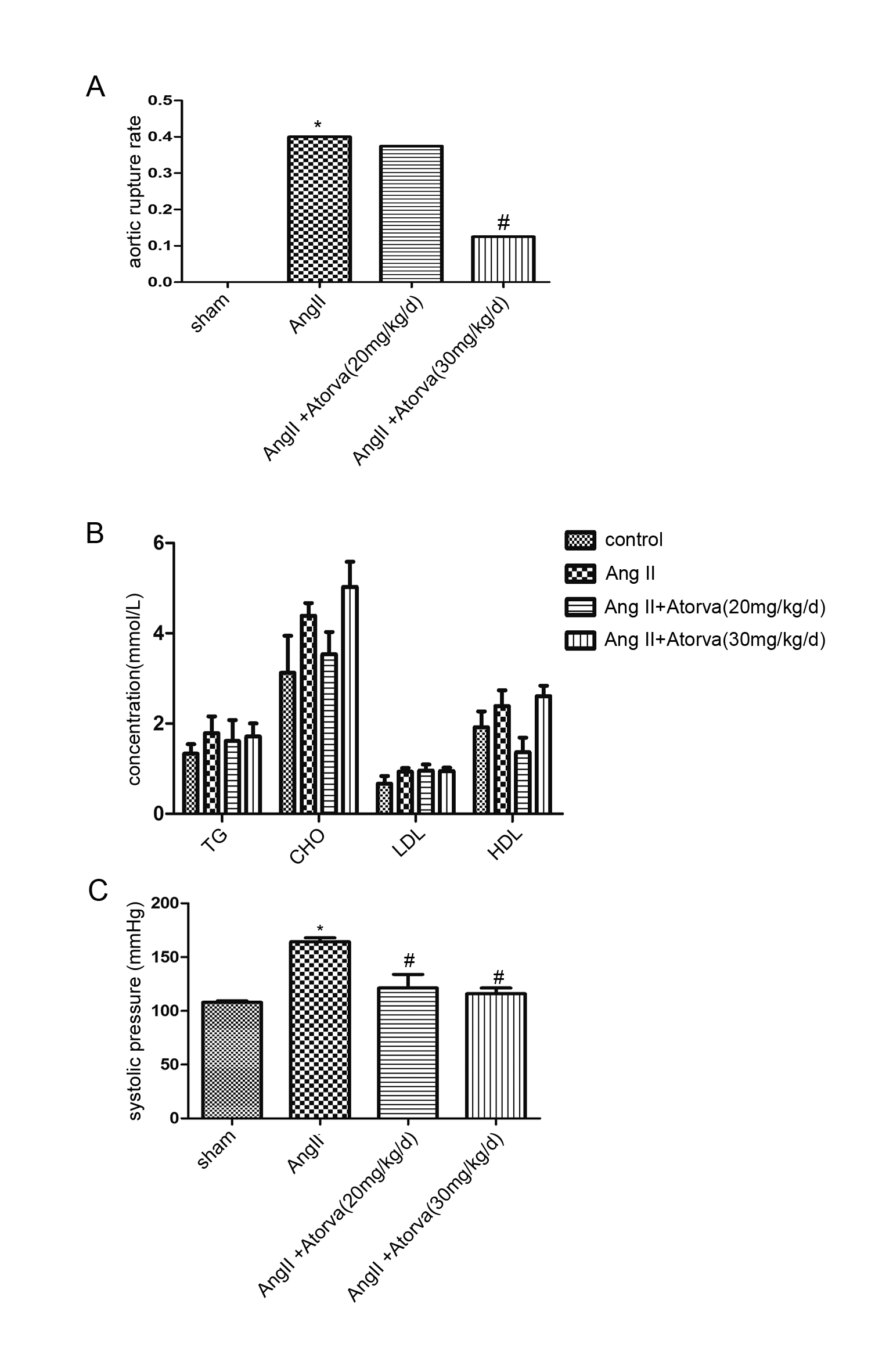

Supplement: S1 Fig — (A) Aortic rupture rate of the 3 groups respectively are: 0.4, 0.375, 0.125. N = 5–10. (B) Lipid levels: there was no statistical significance of lipid levels among groups. N = 3–5. (C) systolic blood pressure: N = 5–6. *P < 0.05 versus control. #P<0.05 versus Ang II infusion alone. (TIF) [file pone.0174821.s001.tif]

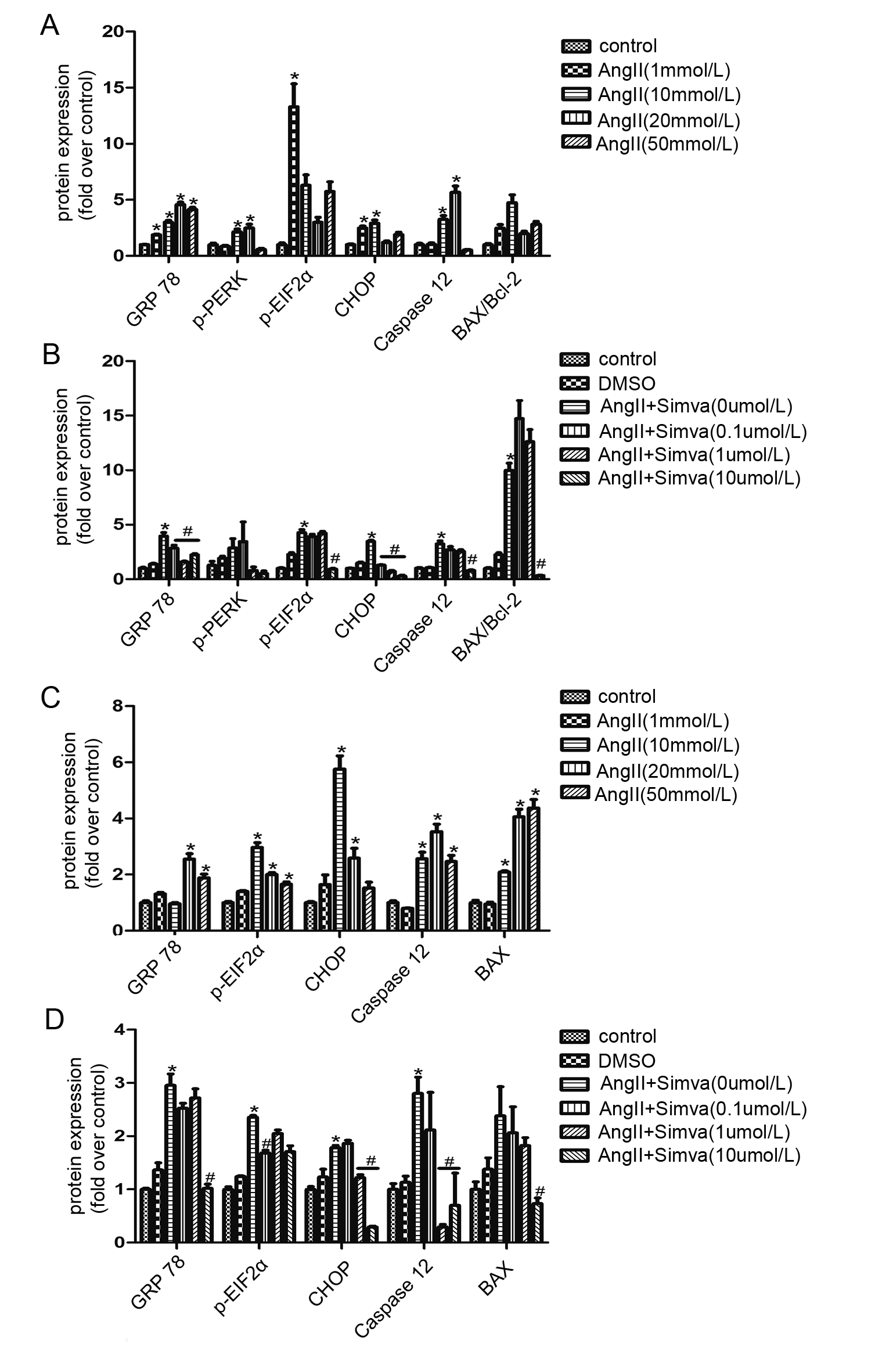

Supplement: S2 Fig — (A) Quantitation of Fig 5A. (B) Quantitation of Fig 6A. (C) Quantitation of Fig 5B. (D) Quantitation of Fig 6B. N = 3. *P < 0.05 versus control. #P<0.05 versus Ang II+ Simva(0umol/L). (TIF) [file pone.0174821.s002.tif]

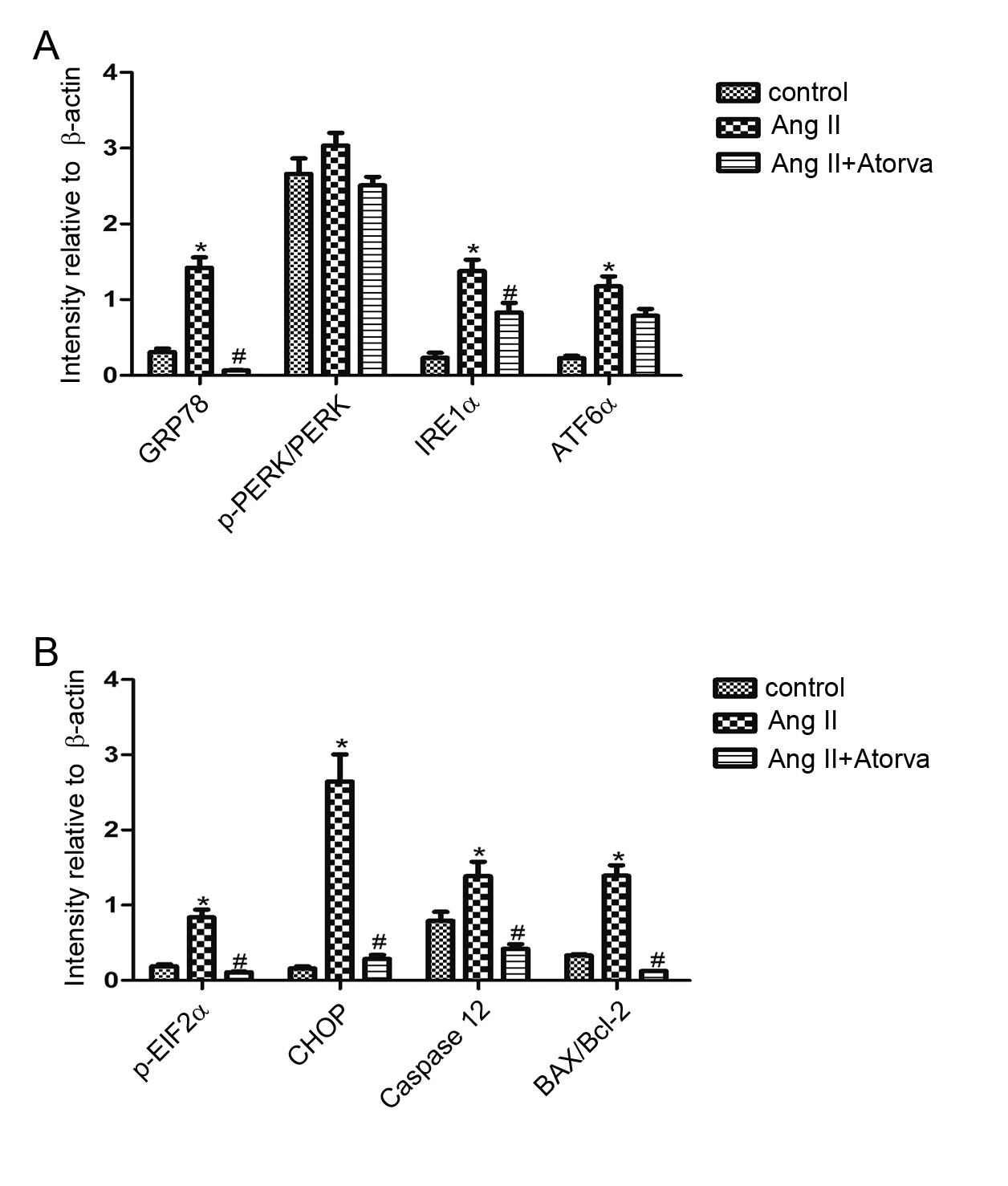

Supplement: S3 Fig — (A) Quantitation of Fig 3C. (B) Quantitation of Fig 3D. N = 3. *P < 0.05 versus control. #P<0.05 versus Ang II infusion alone. (TIF) [file pone.0174821.s003.tif]

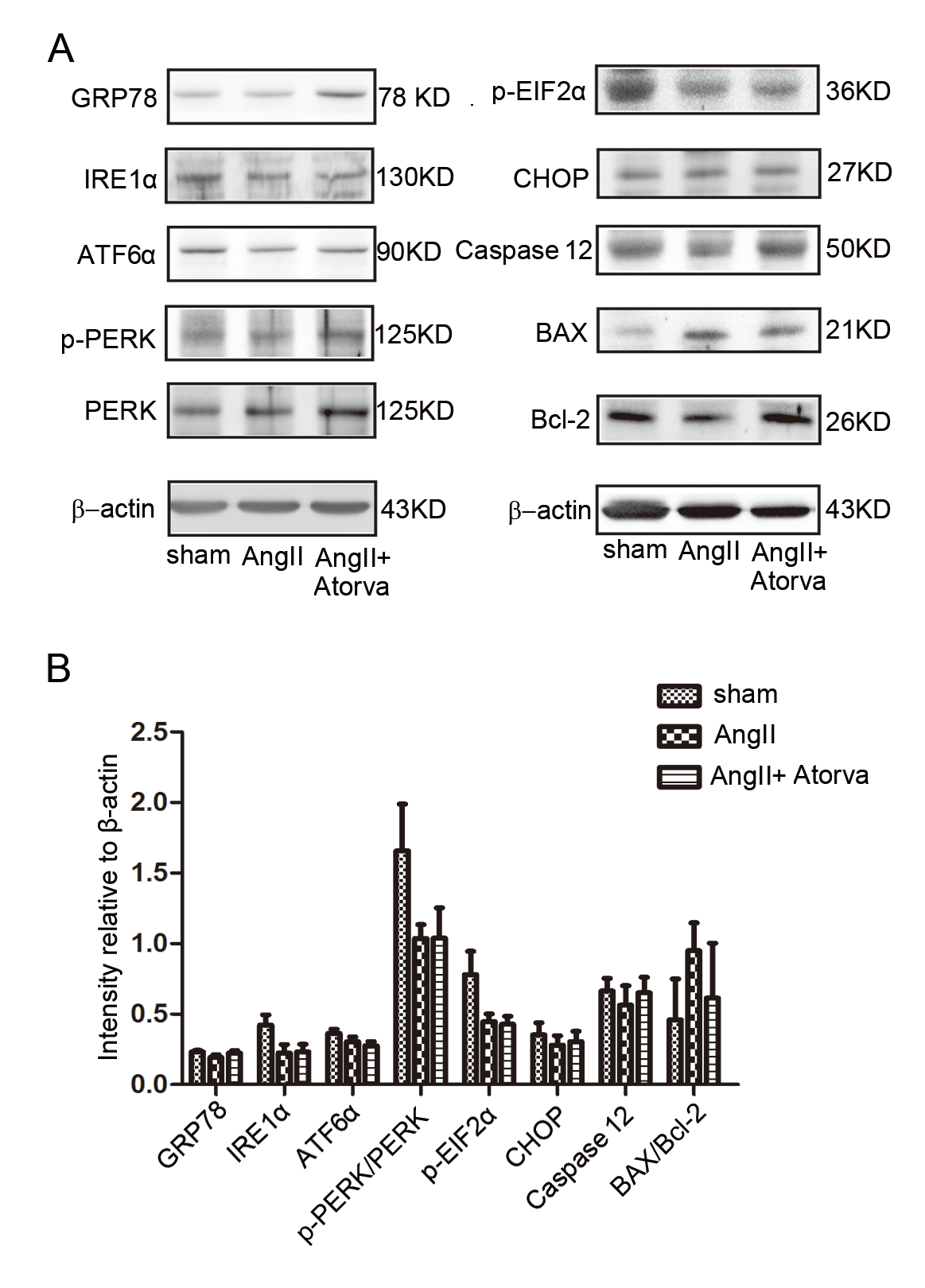

Supplement: S4 Fig — (A) Western blots show PERK-p-EIF2α-CHOP apoptosis pathway. (B) Quantitation of Fig A. N = 3. It indicates no significant difference among groups. (TIF) [file pone.0174821.s004.tif]

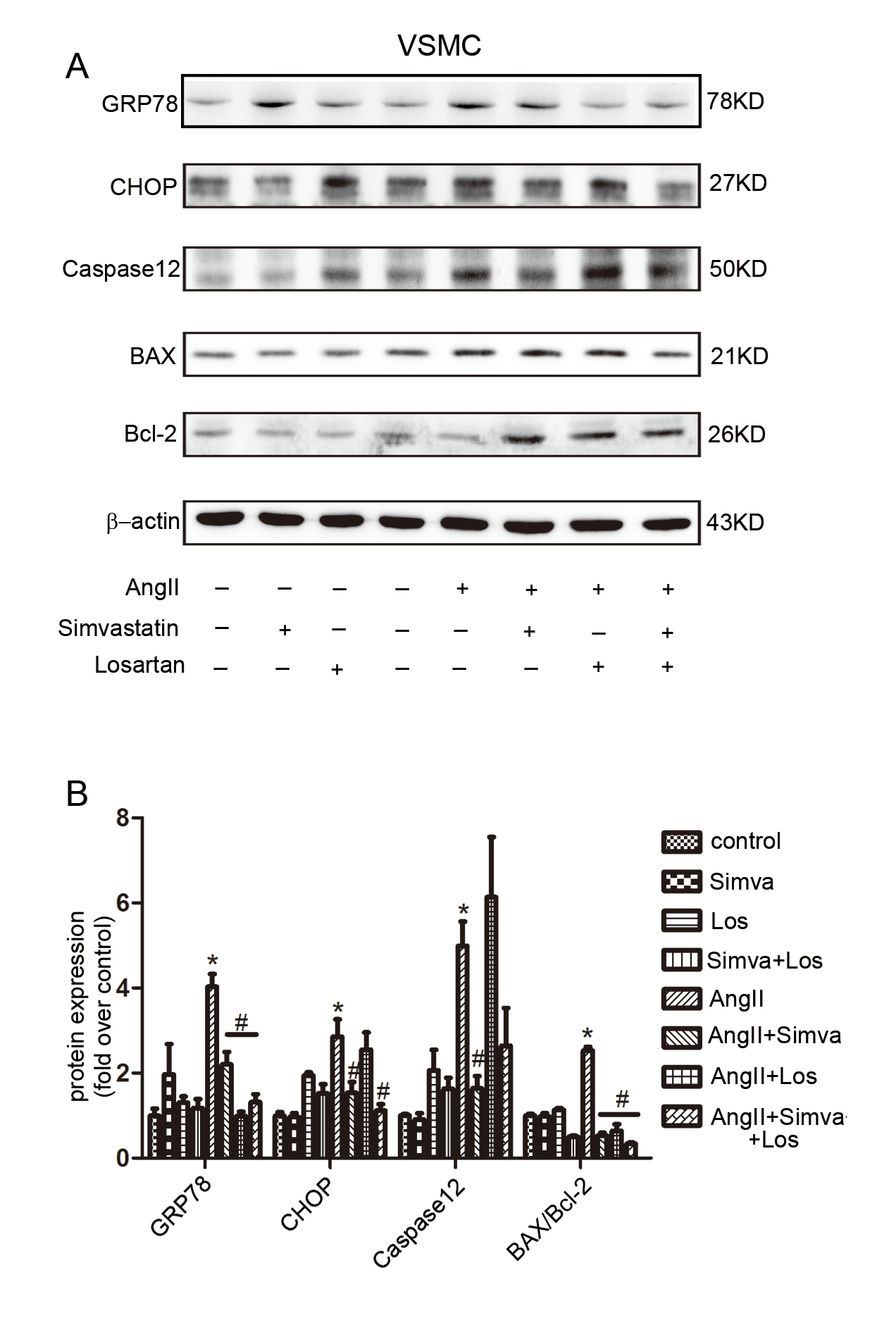

Supplement: S5 Fig — (A) Representative immunoblots of ER stress pathway protein induced by Ang II (20μmol/l) with pretreatment of simvastatin (10μmol/l), Lorsatan(10μmol/l) or both in vascular smooth muscle cells. (B) Quantitation of Fig A. *P < 0.05 versus control. #P<0.05 versus Ang II. Data are shown from three independent experiments. (TIF) [file pone.0174821.s005.tif]

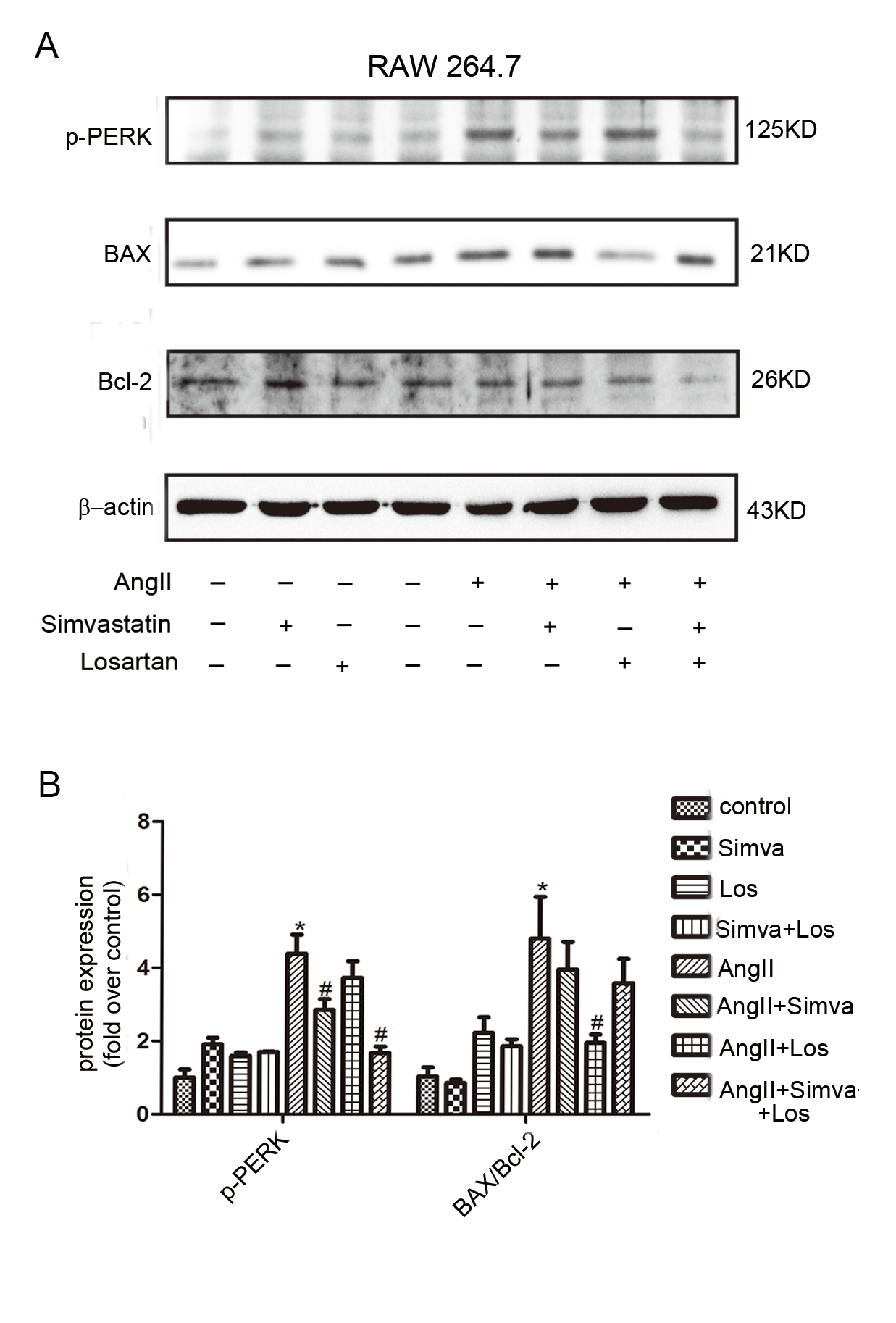

Supplement: S6 Fig — (A) Representative immunoblots of ER stress pathway protein induced by Ang II (20μmol/l) with pretreatment of simvastatin (10μmol/l), Lorsatan(10μmol/l) or both. (B) Quantitation of Fig A. *P < 0.05 versus control. #P<0.05 versus Ang II. Data are shown from three independent experiments. (TIF) [file pone.0174821.s006.tif]

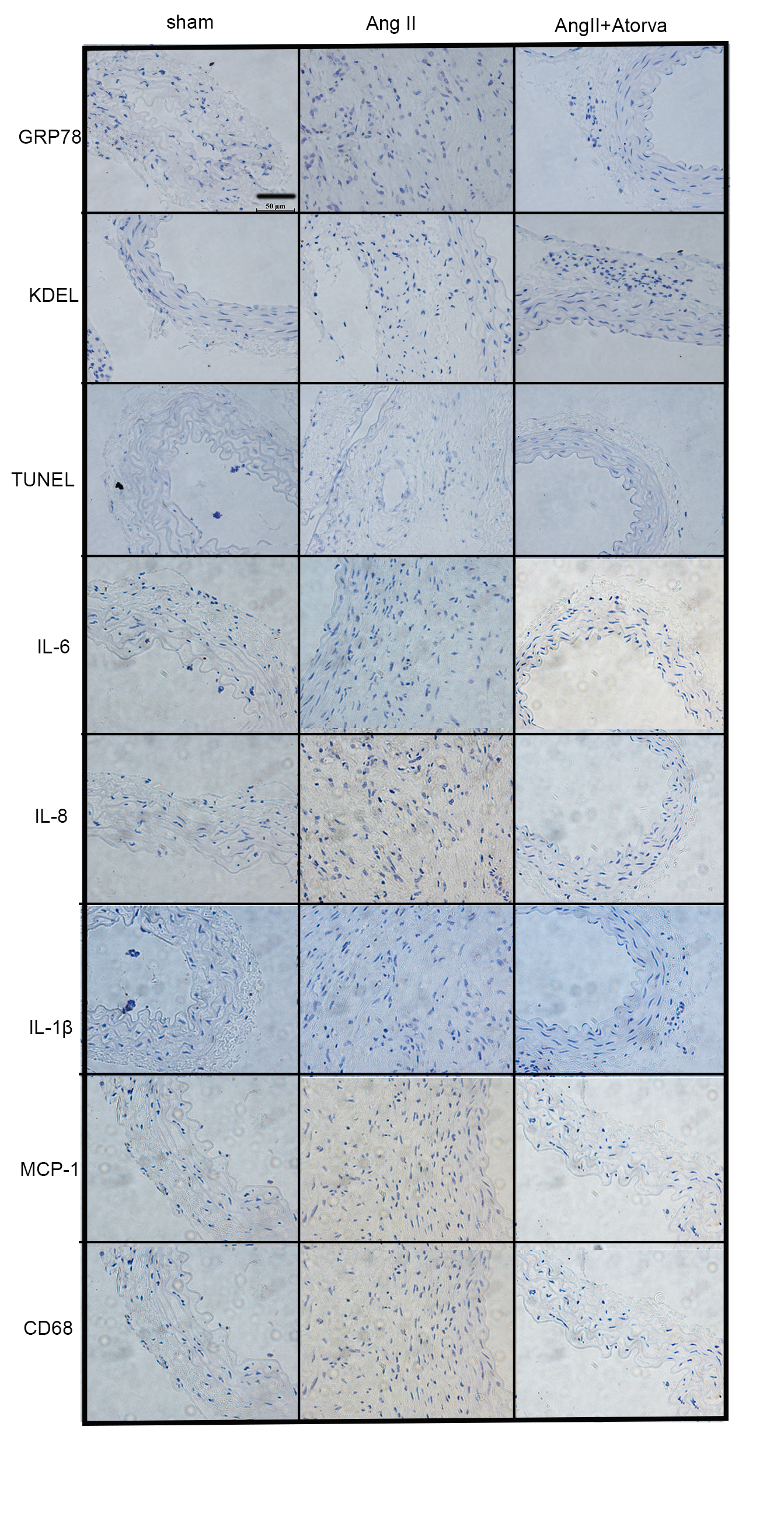

Supplement: S7 Fig — Negative controls of immunostaining of GRP78, KDEL, TUNEL, IL-6, IL-8, IL-1β, CD68, MCP-1. (TIF) [file pone.0174821.s007.tif]

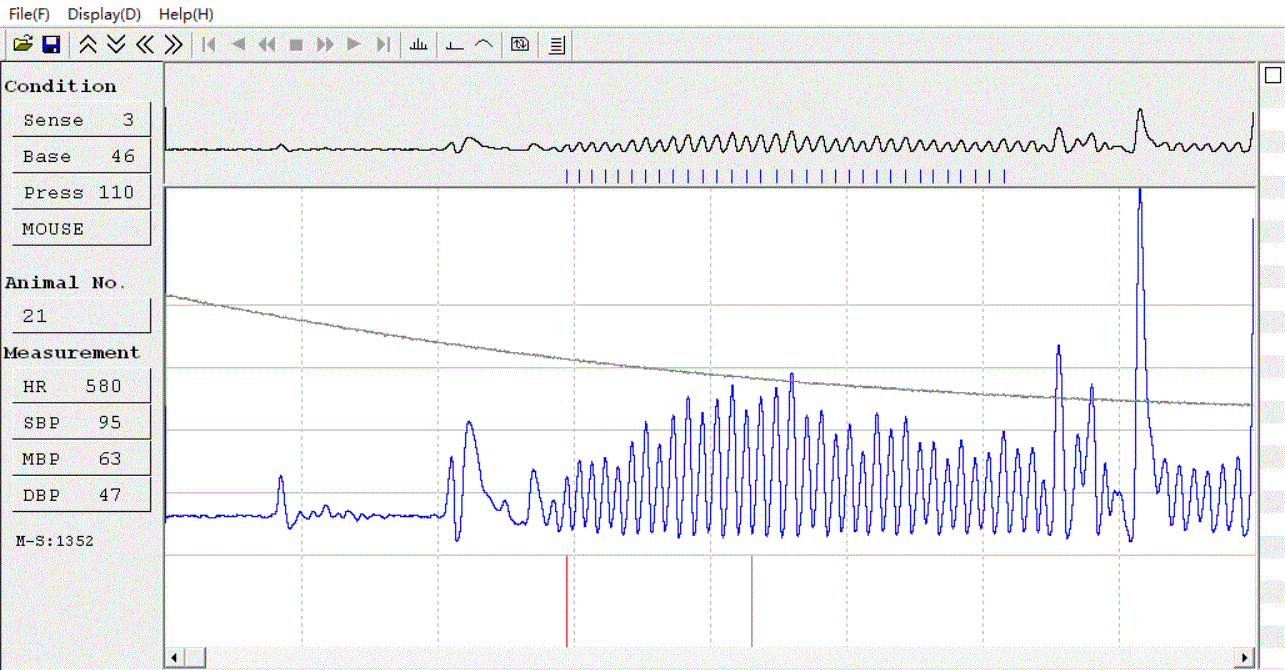

Supplement: S1 File — Pressure data of Ang II-induced AAA model and individual data points corresponding to each statistical graph. (ZIP) [file pone.0174821.s009.zip › Supplyment Data/Ang II model pressure/Image of pressure/1-1.GIF]

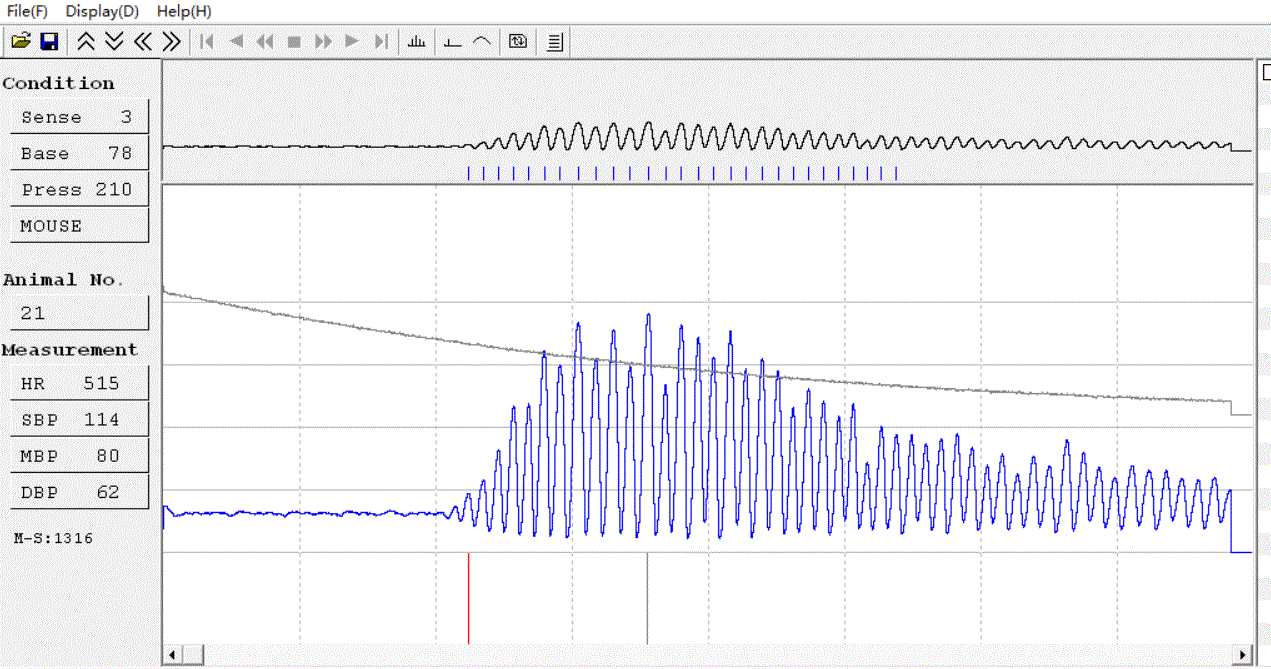

Supplement: S1 File — Pressure data of Ang II-induced AAA model and individual data points corresponding to each statistical graph. (ZIP) [file pone.0174821.s009.zip › Supplyment Data/Ang II model pressure/Image of pressure/1-2.GIF]

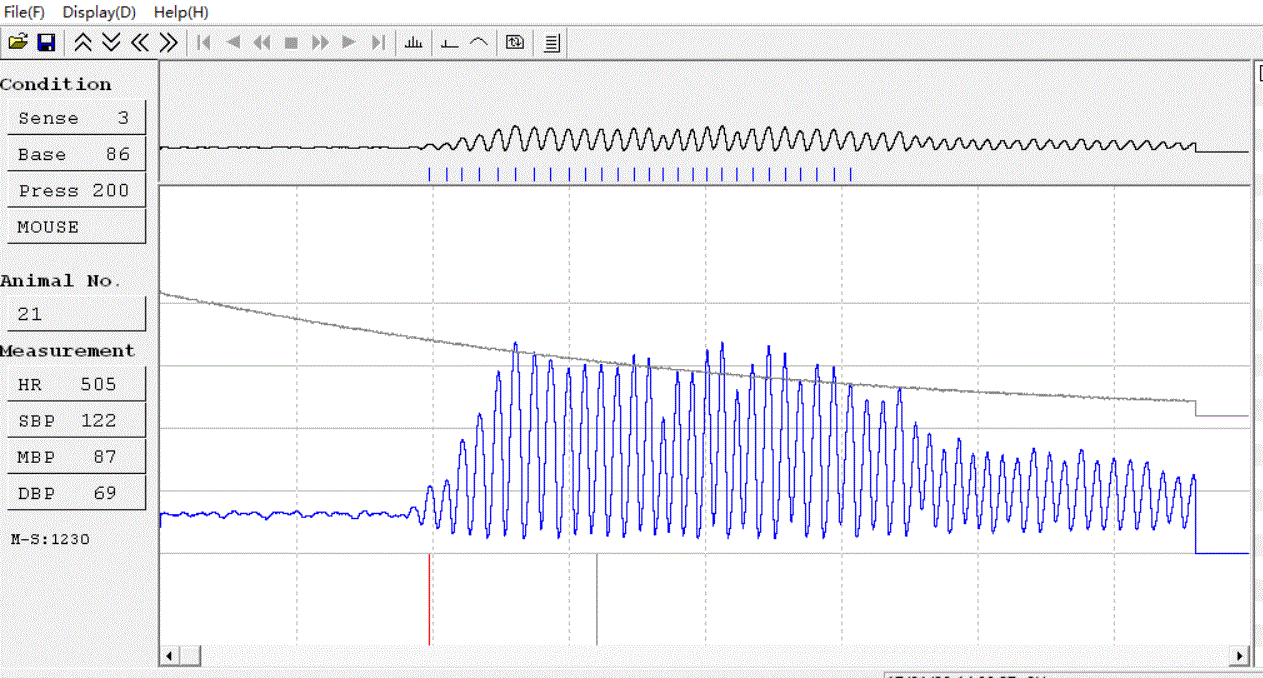

Supplement: S1 File — Pressure data of Ang II-induced AAA model and individual data points corresponding to each statistical graph. (ZIP) [file pone.0174821.s009.zip › Supplyment Data/Ang II model pressure/Image of pressure/1-3.GIF]

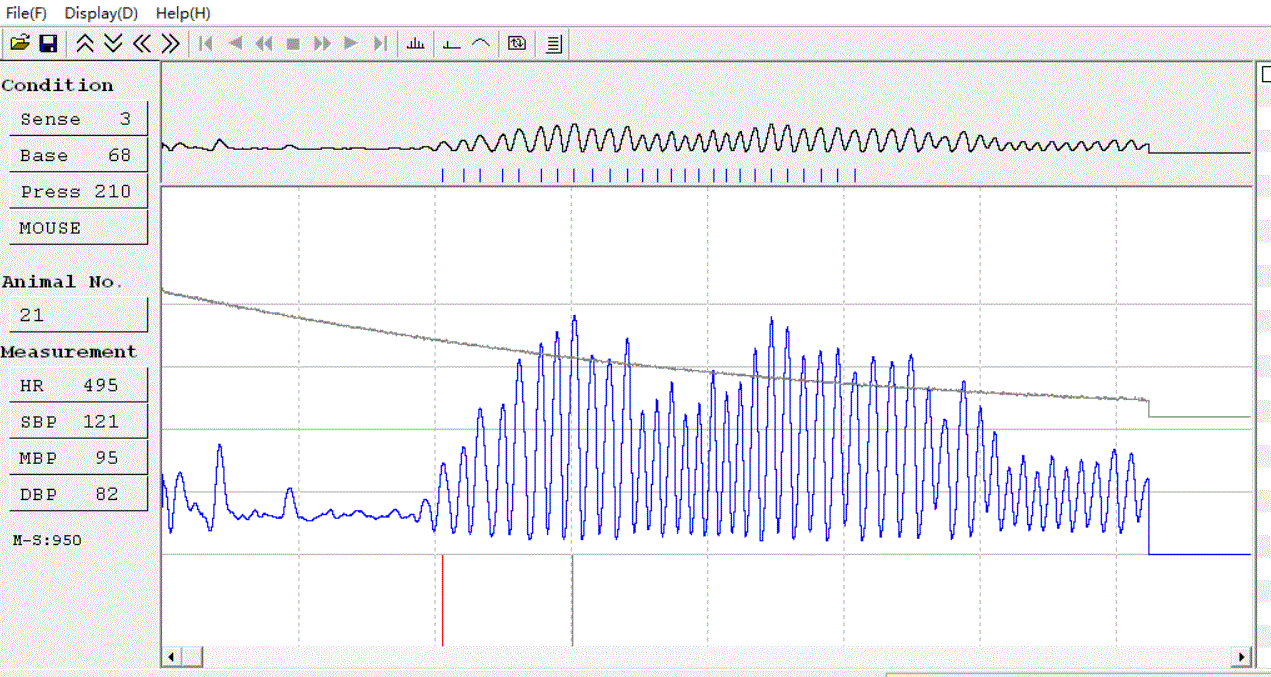

Supplement: S1 File — Pressure data of Ang II-induced AAA model and individual data points corresponding to each statistical graph. (ZIP) [file pone.0174821.s009.zip › Supplyment Data/Ang II model pressure/Image of pressure/2-1.GIF]

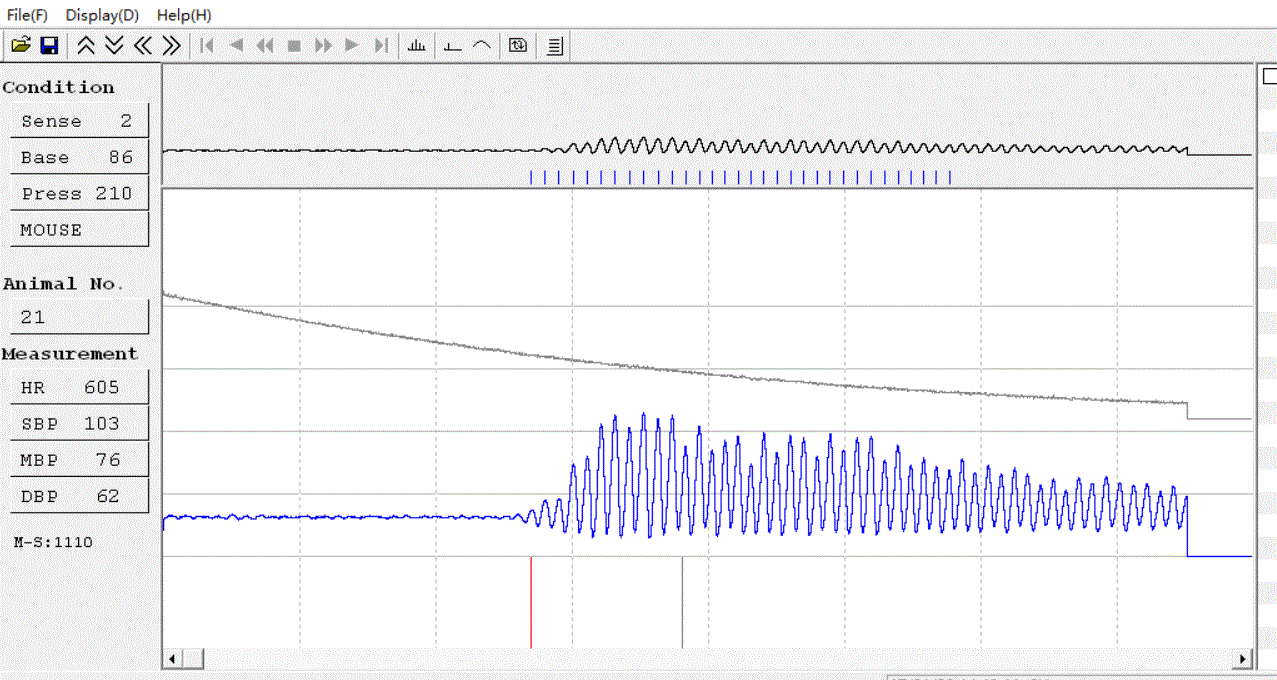

Supplement: S1 File — Pressure data of Ang II-induced AAA model and individual data points corresponding to each statistical graph. (ZIP) [file pone.0174821.s009.zip › Supplyment Data/Ang II model pressure/Image of pressure/2-2.GIF]

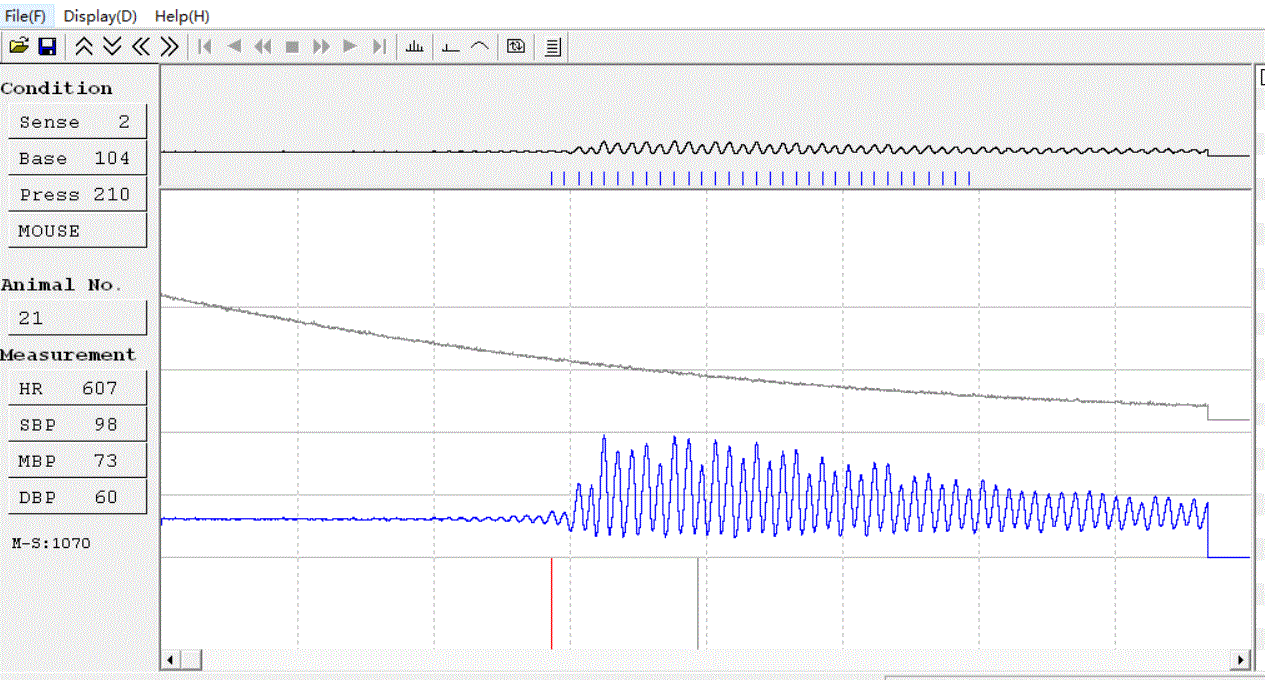

Supplement: S1 File — Pressure data of Ang II-induced AAA model and individual data points corresponding to each statistical graph. (ZIP) [file pone.0174821.s009.zip › Supplyment Data/Ang II model pressure/Image of pressure/2-3.GIF]

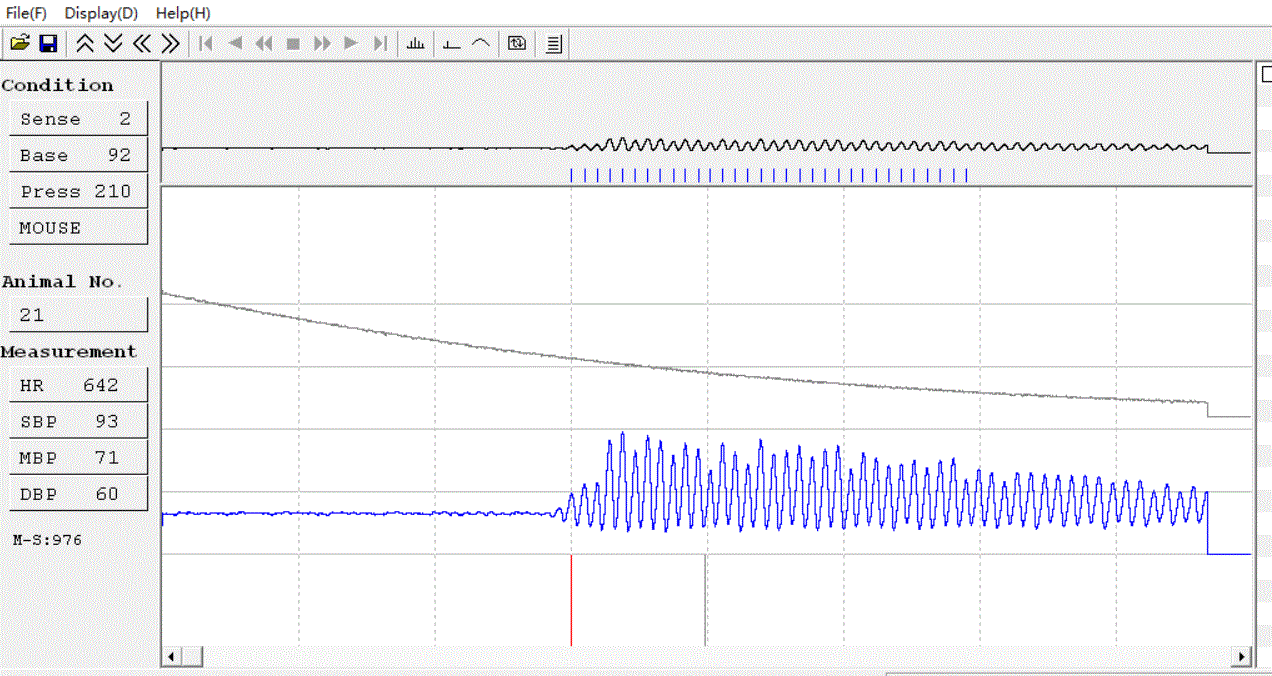

Supplement: S1 File — Pressure data of Ang II-induced AAA model and individual data points corresponding to each statistical graph. (ZIP) [file pone.0174821.s009.zip › Supplyment Data/Ang II model pressure/Image of pressure/3-1.GIF]

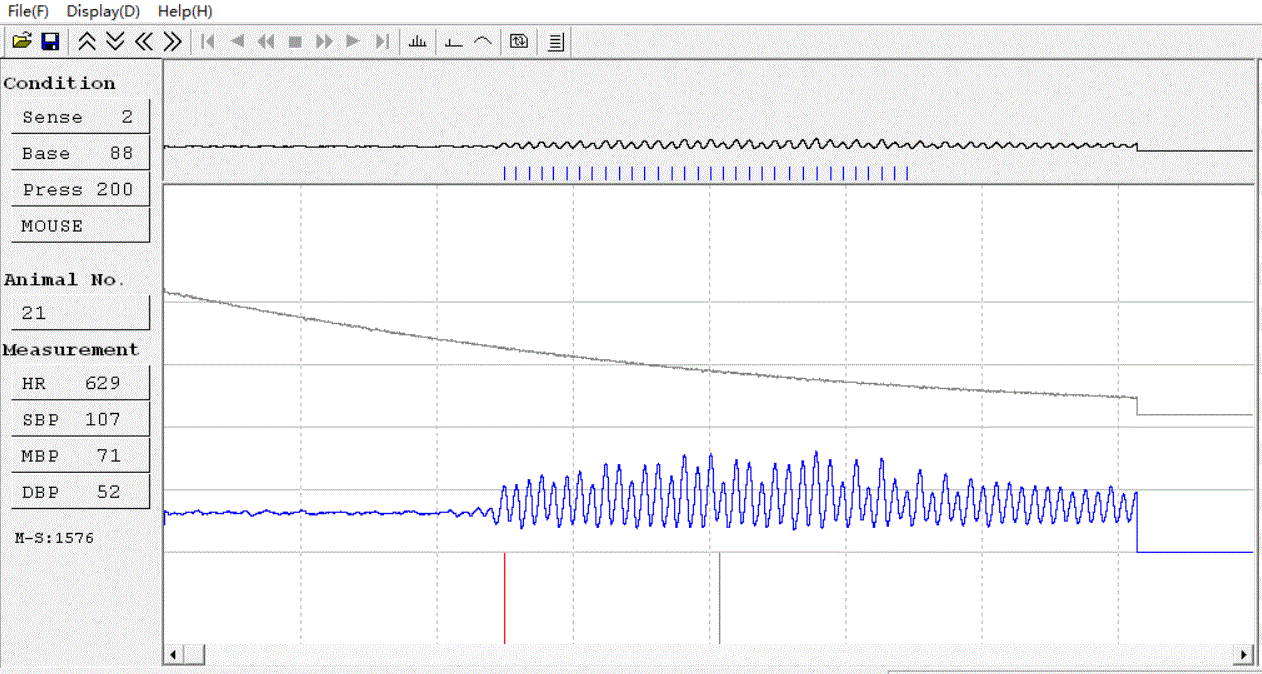

Supplement: S1 File — Pressure data of Ang II-induced AAA model and individual data points corresponding to each statistical graph. (ZIP) [file pone.0174821.s009.zip › Supplyment Data/Ang II model pressure/Image of pressure/3-2.GIF]

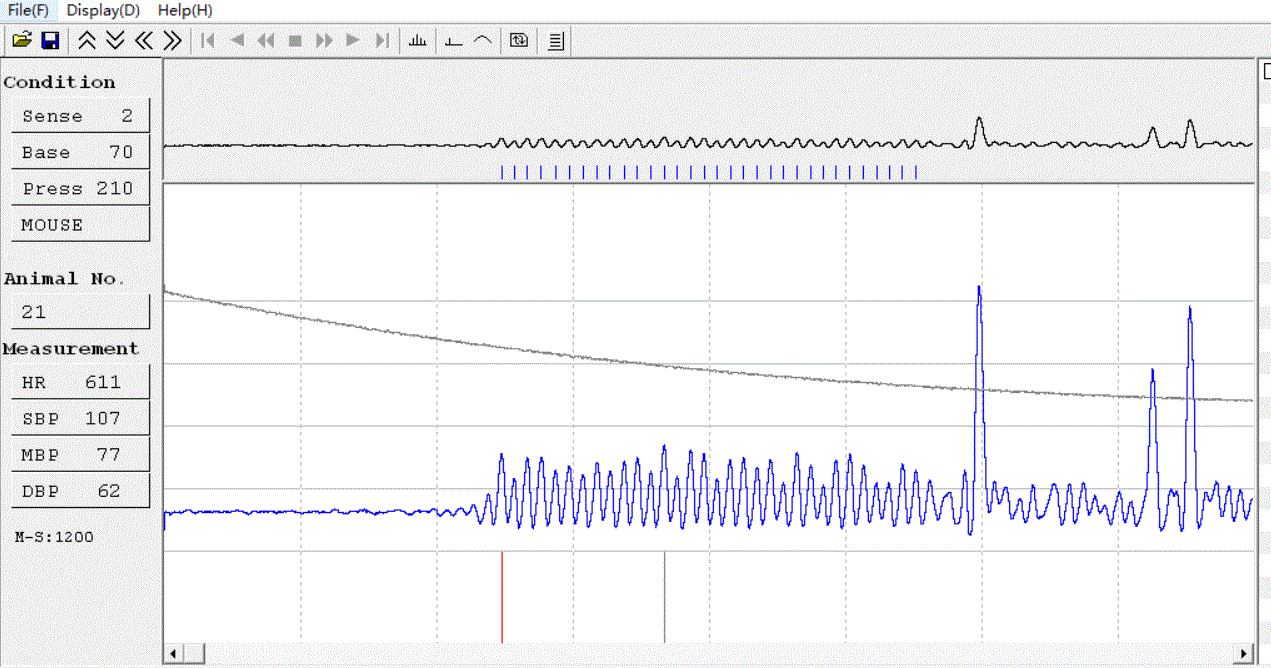

Supplement: S1 File — Pressure data of Ang II-induced AAA model and individual data points corresponding to each statistical graph. (ZIP) [file pone.0174821.s009.zip › Supplyment Data/Ang II model pressure/Image of pressure/3-3.GIF]

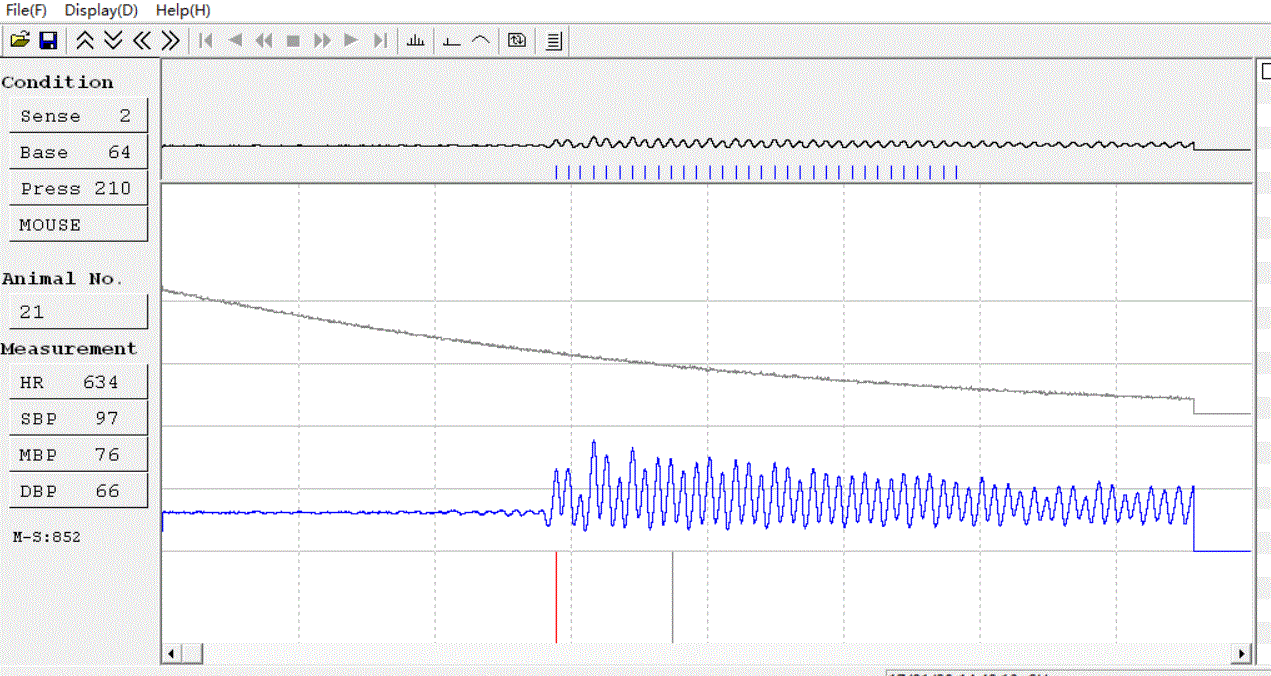

Supplement: S1 File — Pressure data of Ang II-induced AAA model and individual data points corresponding to each statistical graph. (ZIP) [file pone.0174821.s009.zip › Supplyment Data/Ang II model pressure/Image of pressure/4-1.GIF]

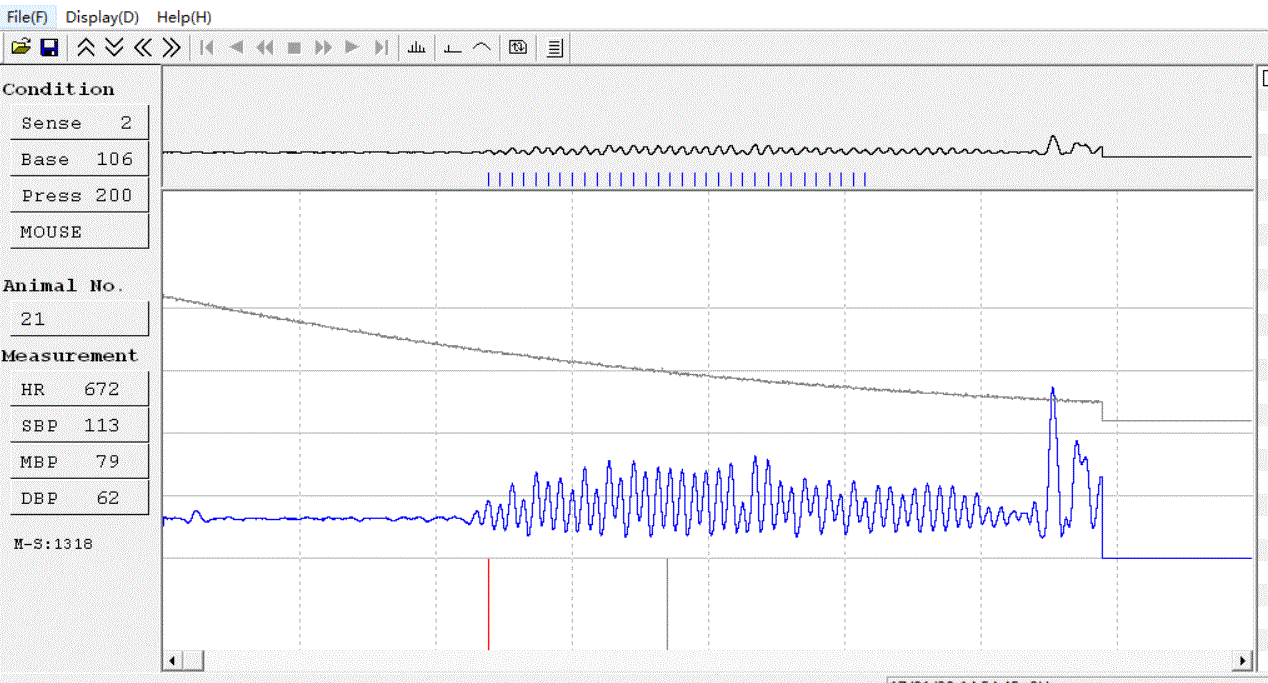

Supplement: S1 File — Pressure data of Ang II-induced AAA model and individual data points corresponding to each statistical graph. (ZIP) [file pone.0174821.s009.zip › Supplyment Data/Ang II model pressure/Image of pressure/4-2.GIF]

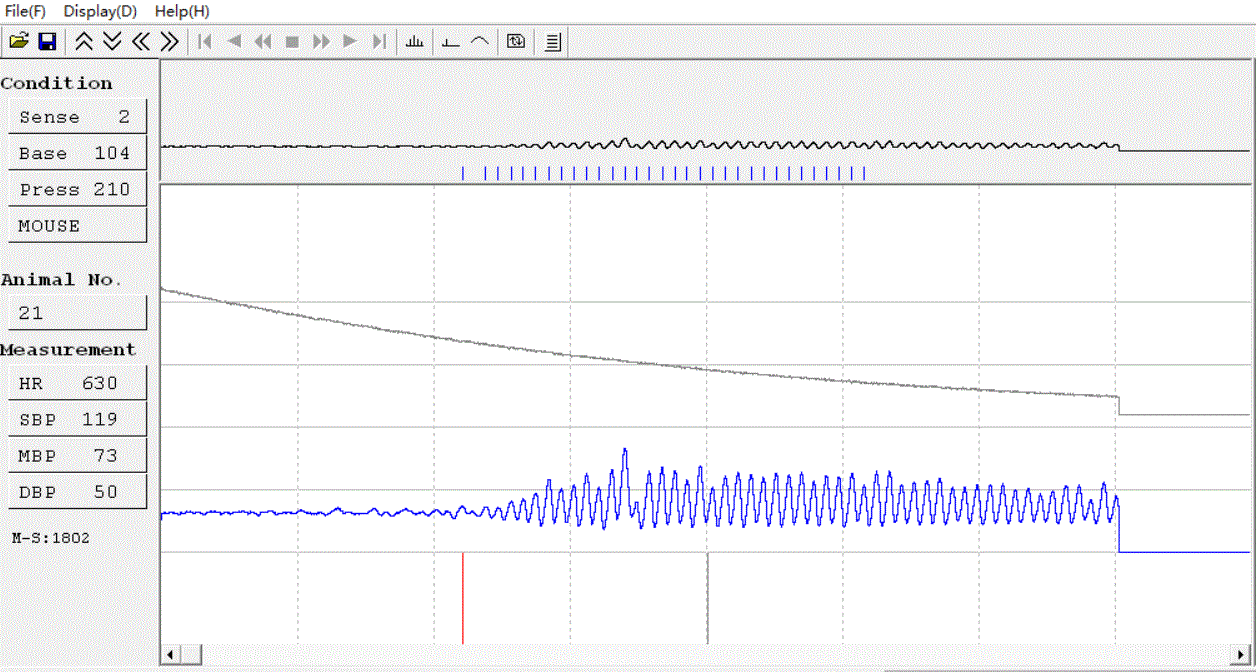

Supplement: S1 File — Pressure data of Ang II-induced AAA model and individual data points corresponding to each statistical graph. (ZIP) [file pone.0174821.s009.zip › Supplyment Data/Ang II model pressure/Image of pressure/4-3.GIF]

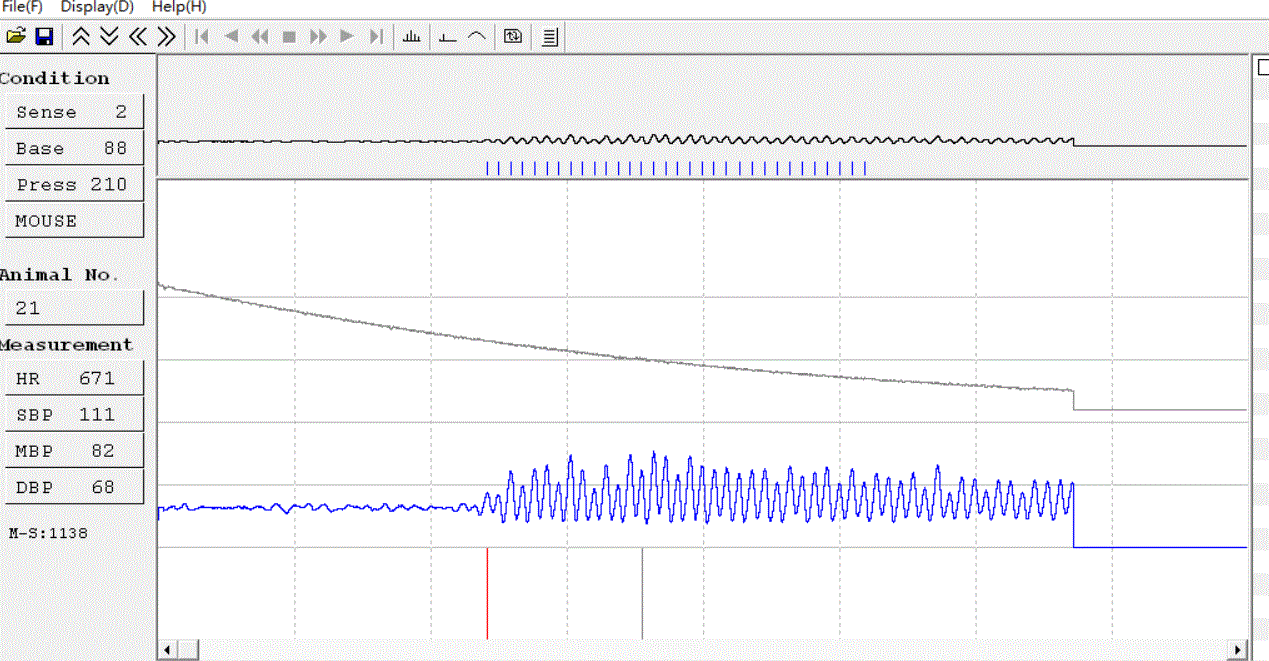

Supplement: S1 File — Pressure data of Ang II-induced AAA model and individual data points corresponding to each statistical graph. (ZIP) [file pone.0174821.s009.zip › Supplyment Data/Ang II model pressure/Image of pressure/5-1.GIF]

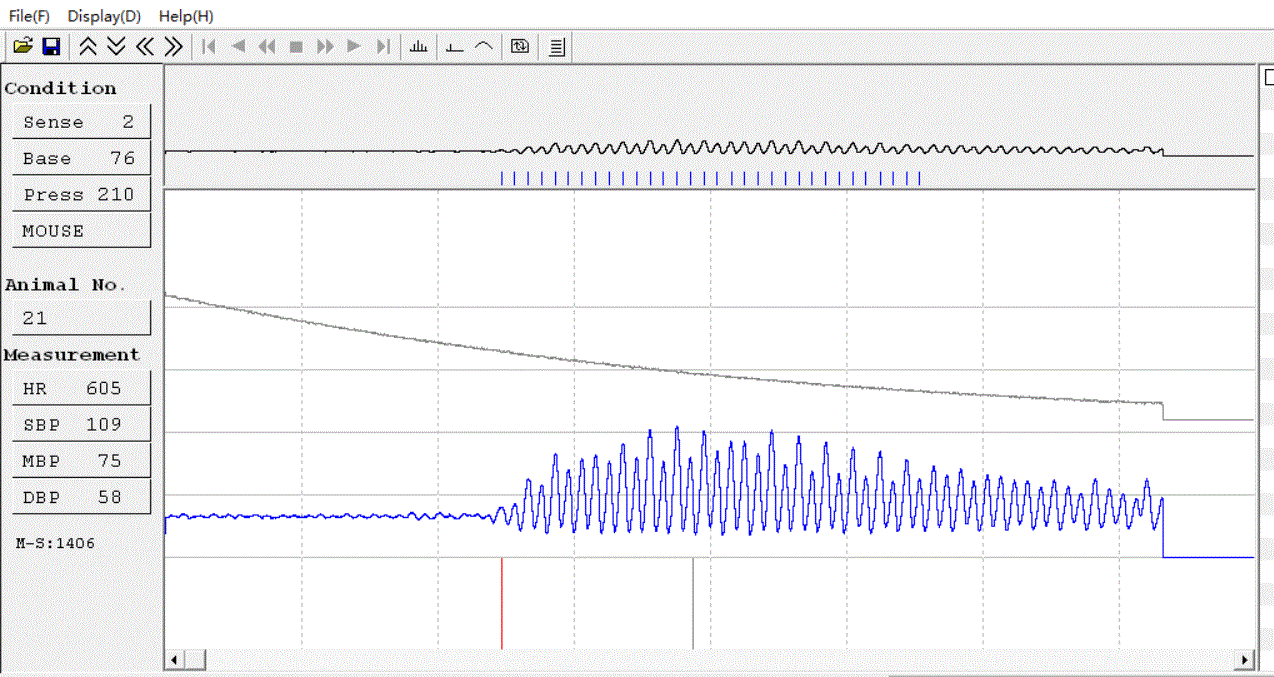

Supplement: S1 File — Pressure data of Ang II-induced AAA model and individual data points corresponding to each statistical graph. (ZIP) [file pone.0174821.s009.zip › Supplyment Data/Ang II model pressure/Image of pressure/5-2.GIF]

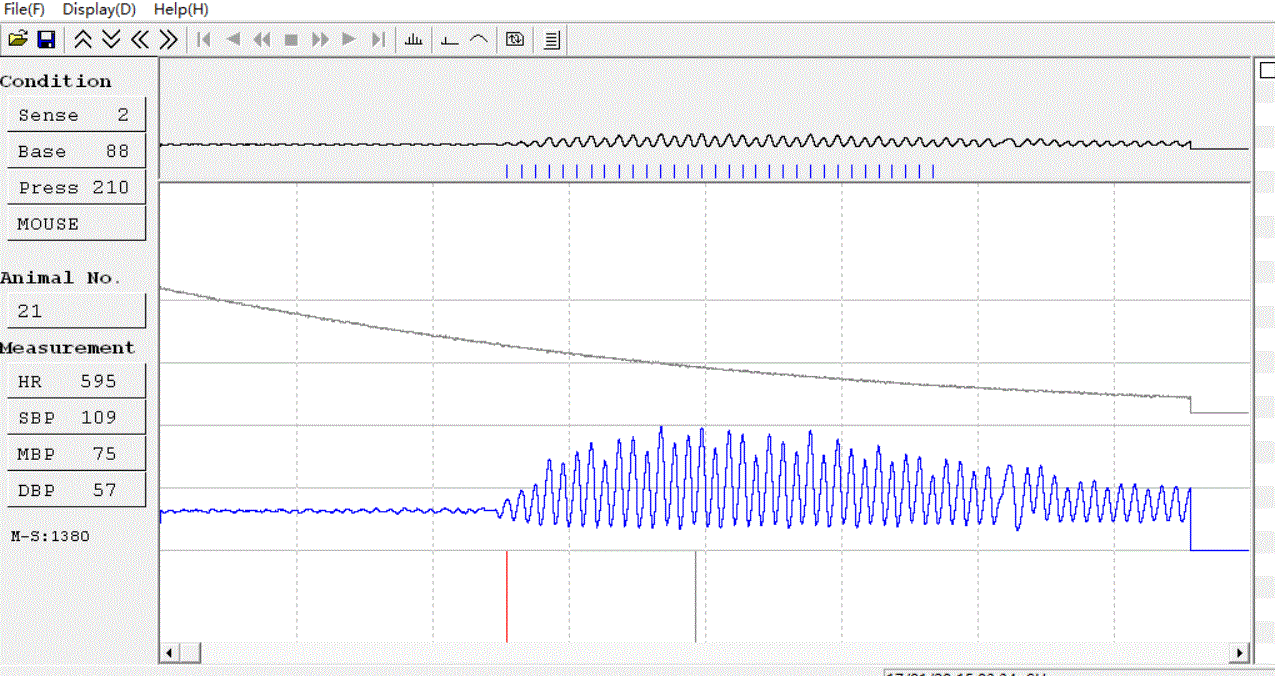

Supplement: S1 File — Pressure data of Ang II-induced AAA model and individual data points corresponding to each statistical graph. (ZIP) [file pone.0174821.s009.zip › Supplyment Data/Ang II model pressure/Image of pressure/5-3.GIF]

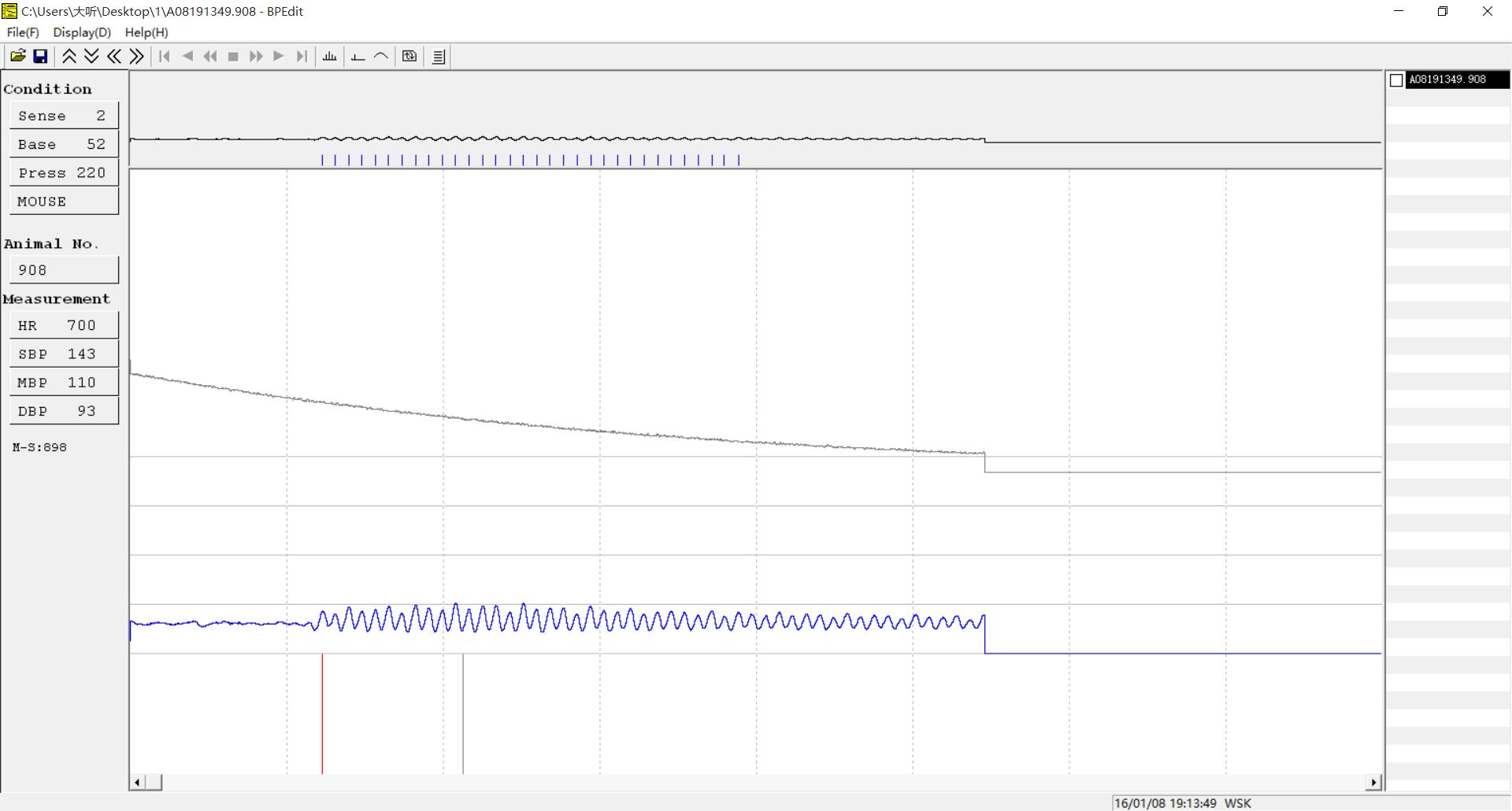

Supplement: S1 File — Pressure data of Ang II-induced AAA model and individual data points corresponding to each statistical graph. (ZIP) [file pone.0174821.s009.zip › Supplyment Data/Ang II model pressure/Image of pressure/A2-1.jpg]

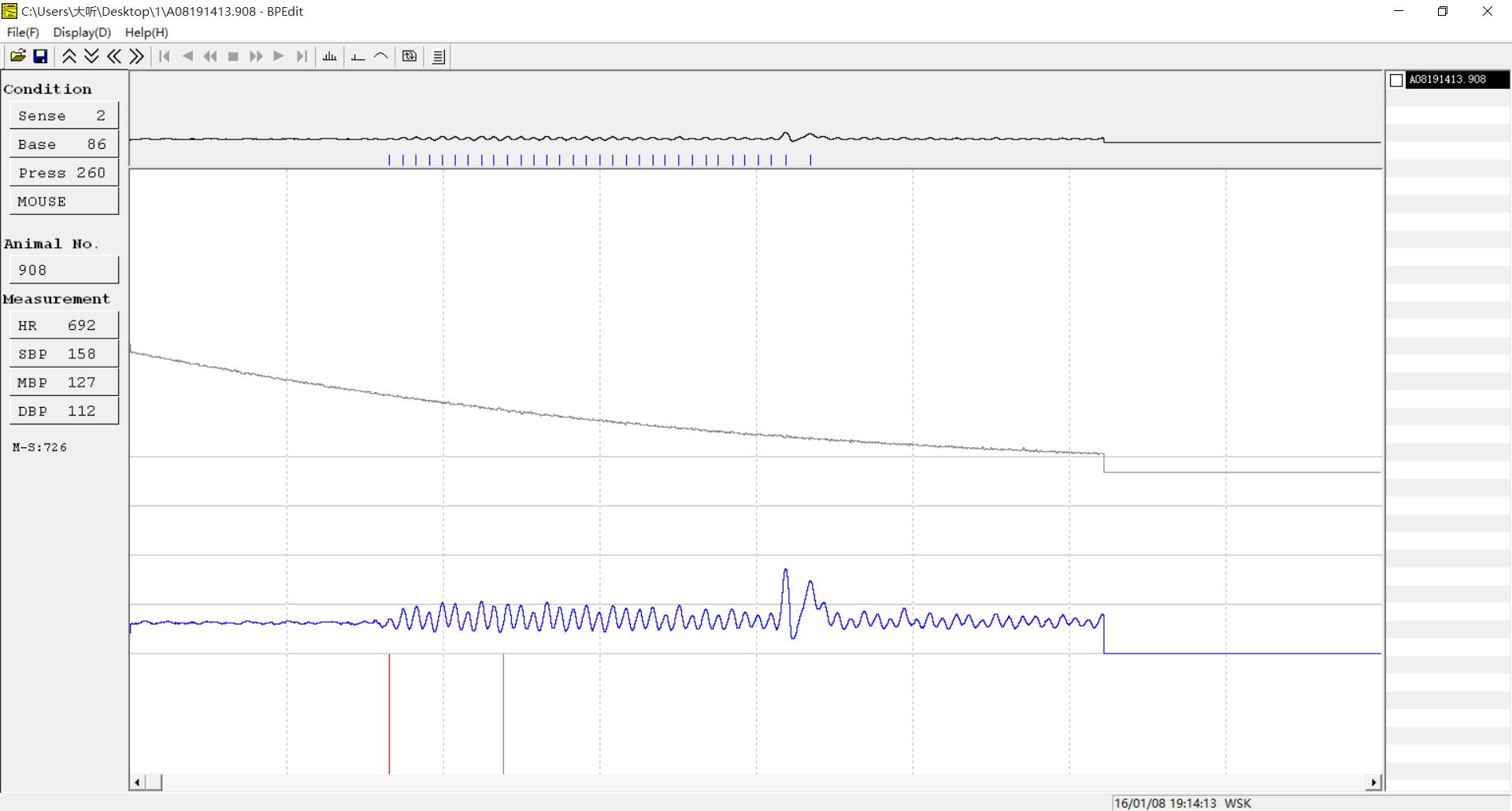

Supplement: S1 File — Pressure data of Ang II-induced AAA model and individual data points corresponding to each statistical graph. (ZIP) [file pone.0174821.s009.zip › Supplyment Data/Ang II model pressure/Image of pressure/A2-2.jpg]

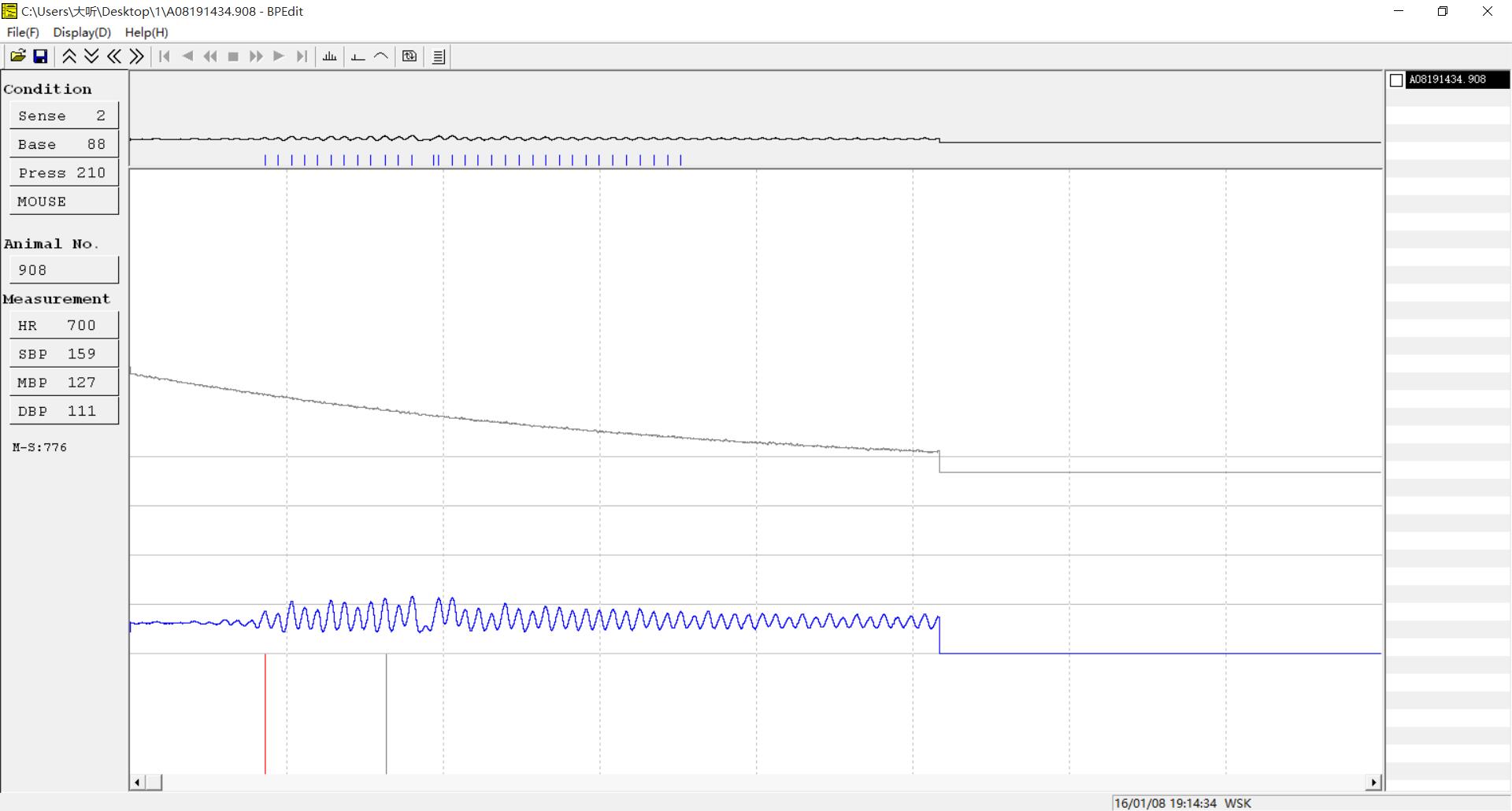

Supplement: S1 File — Pressure data of Ang II-induced AAA model and individual data points corresponding to each statistical graph. (ZIP) [file pone.0174821.s009.zip › Supplyment Data/Ang II model pressure/Image of pressure/A2-3.jpg]

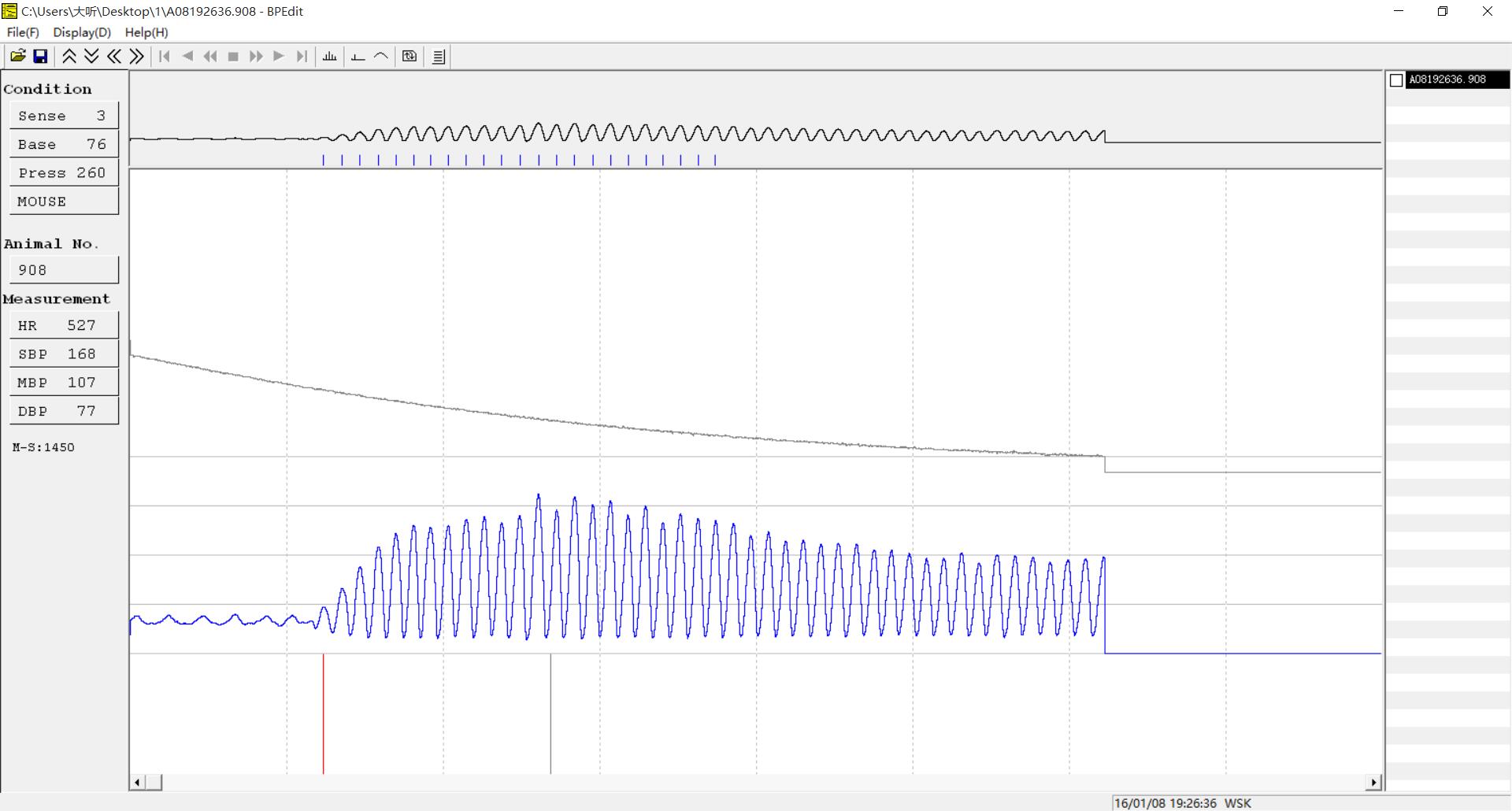

Supplement: S1 File — Pressure data of Ang II-induced AAA model and individual data points corresponding to each statistical graph. (ZIP) [file pone.0174821.s009.zip › Supplyment Data/Ang II model pressure/Image of pressure/A4-1.jpg]

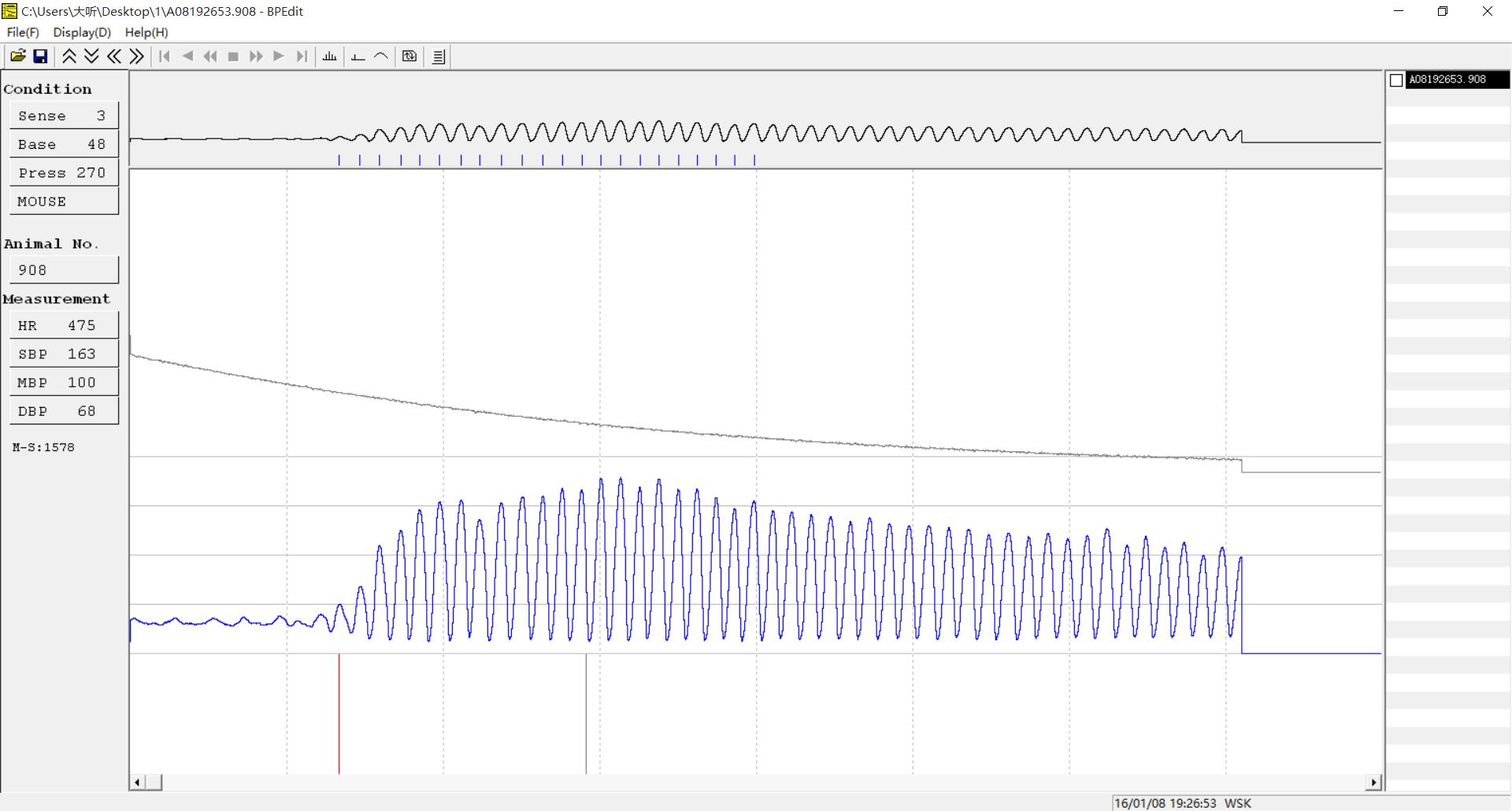

Supplement: S1 File — Pressure data of Ang II-induced AAA model and individual data points corresponding to each statistical graph. (ZIP) [file pone.0174821.s009.zip › Supplyment Data/Ang II model pressure/Image of pressure/A4-2.jpg]

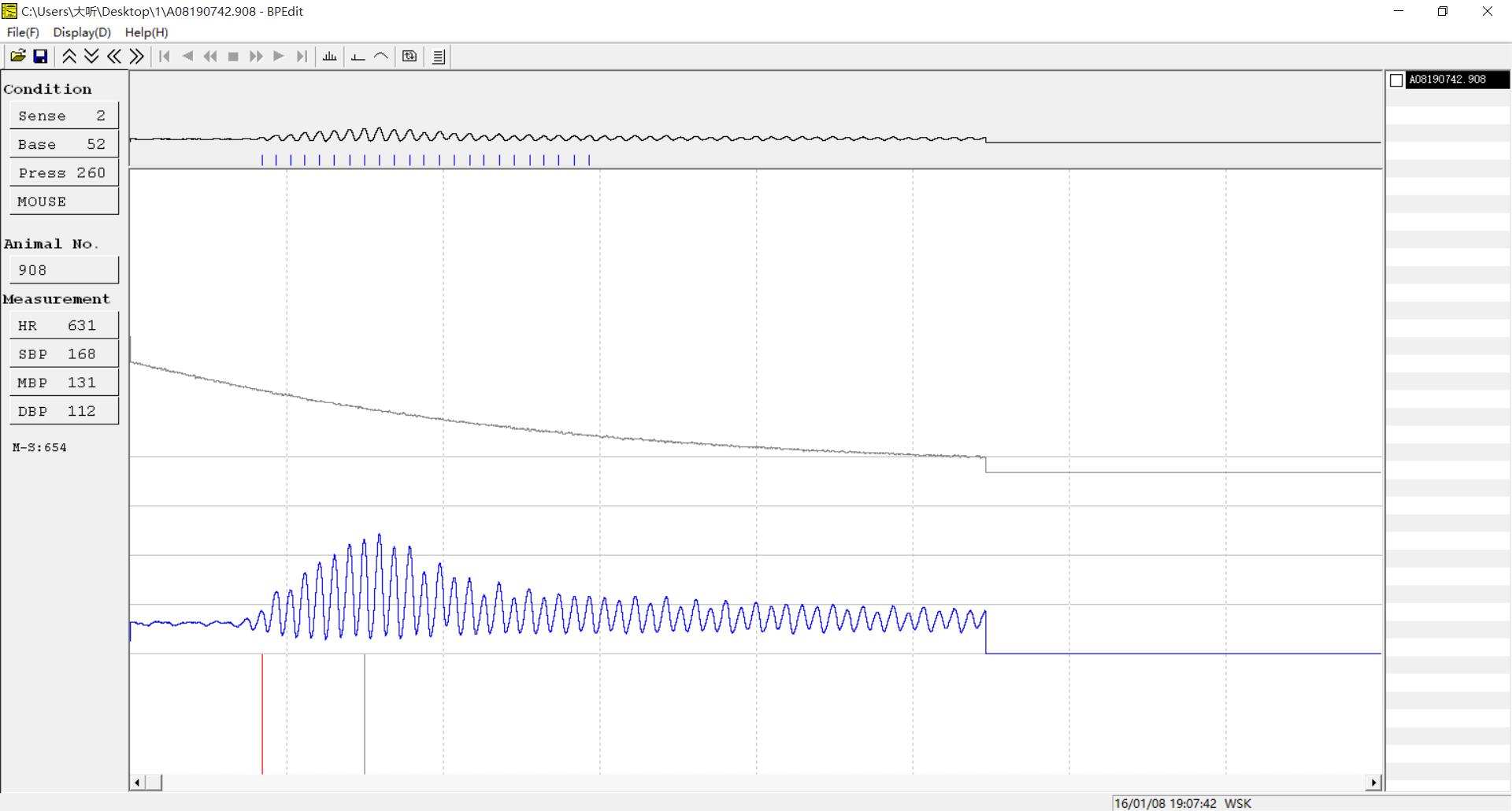

Supplement: S1 File — Pressure data of Ang II-induced AAA model and individual data points corresponding to each statistical graph. (ZIP) [file pone.0174821.s009.zip › Supplyment Data/Ang II model pressure/Image of pressure/A5-1.jpg]

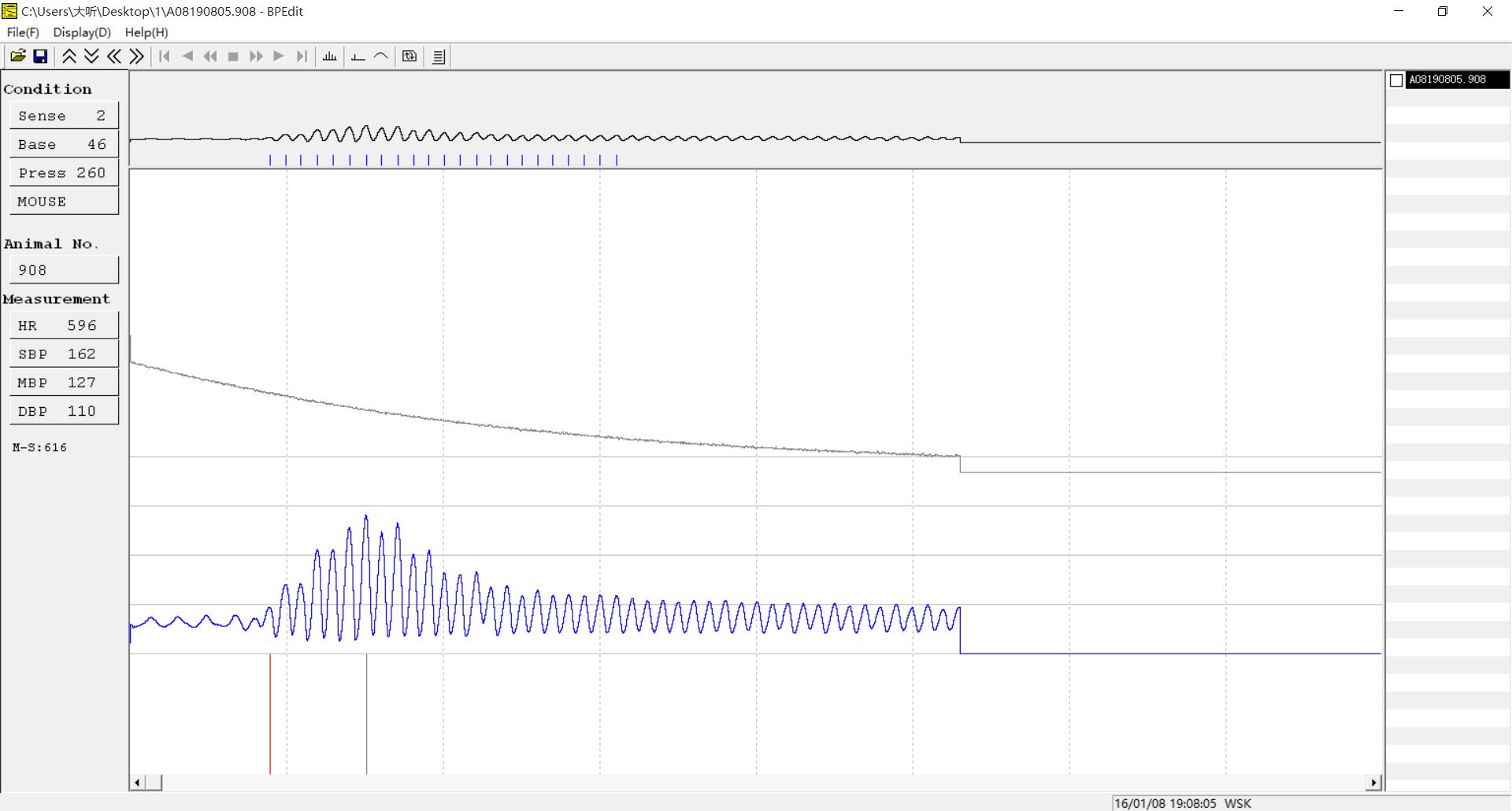

Supplement: S1 File — Pressure data of Ang II-induced AAA model and individual data points corresponding to each statistical graph. (ZIP) [file pone.0174821.s009.zip › Supplyment Data/Ang II model pressure/Image of pressure/A5-2.jpg]

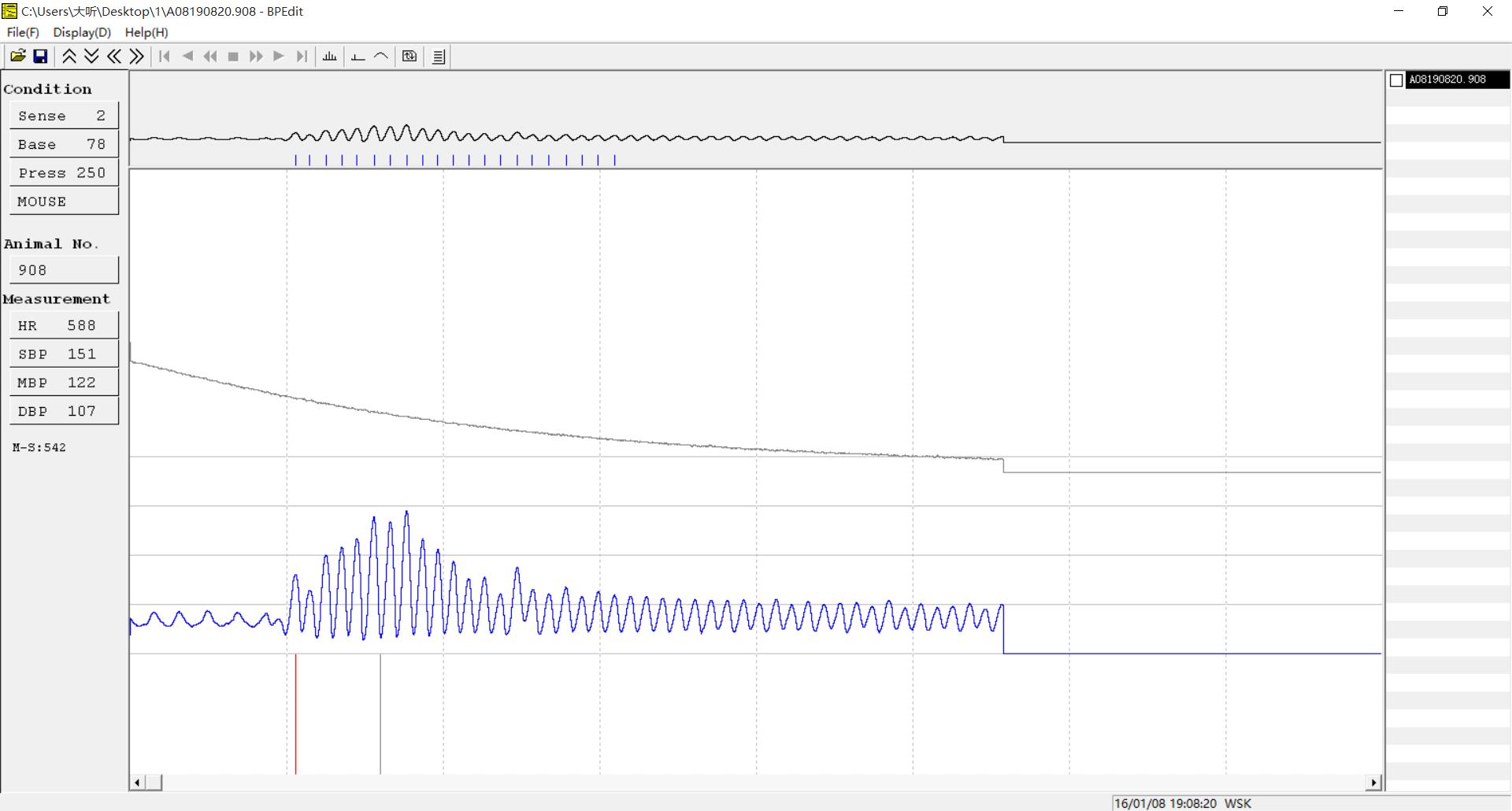

Supplement: S1 File — Pressure data of Ang II-induced AAA model and individual data points corresponding to each statistical graph. (ZIP) [file pone.0174821.s009.zip › Supplyment Data/Ang II model pressure/Image of pressure/A5-3.jpg]

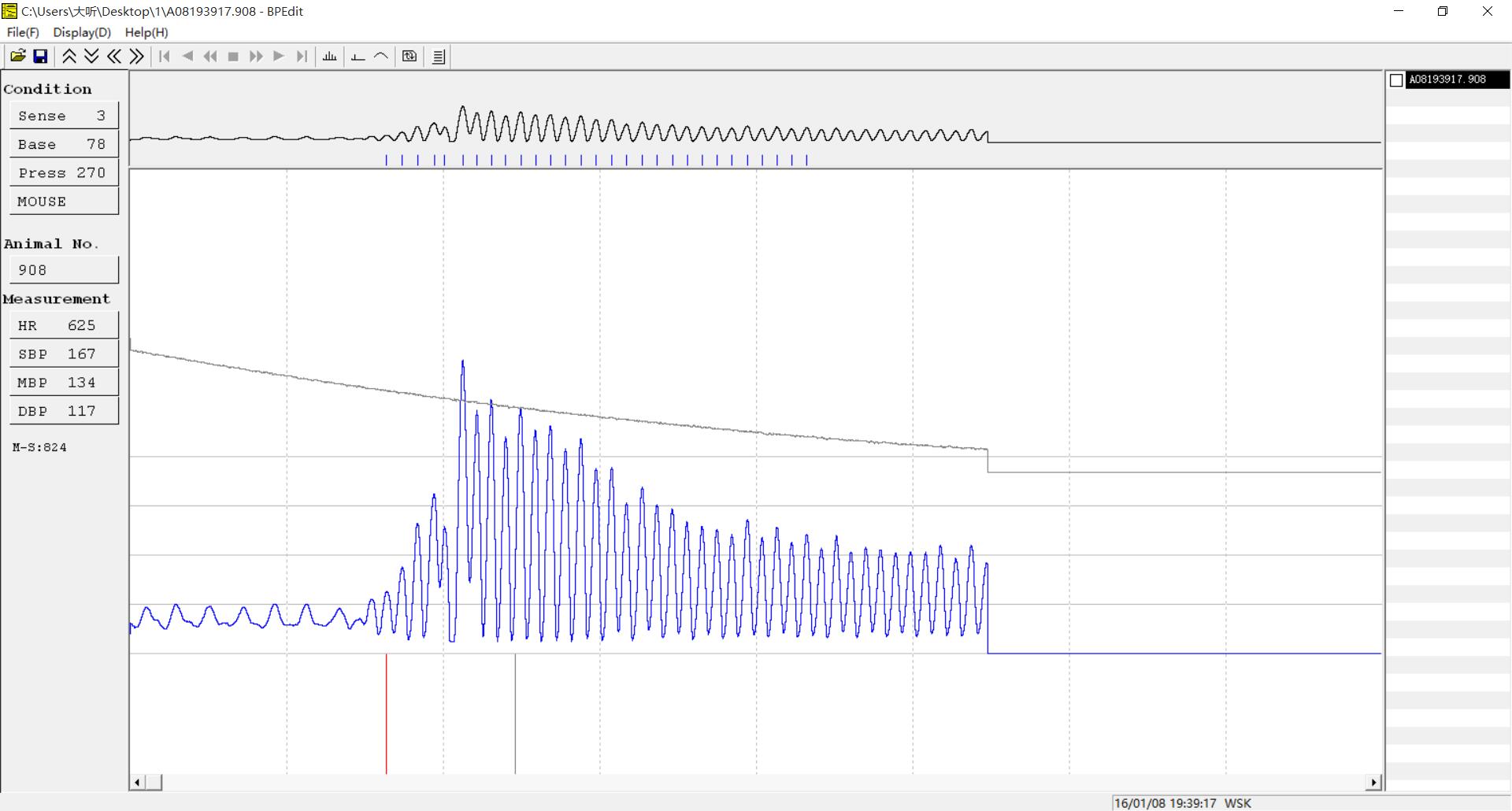

Supplement: S1 File — Pressure data of Ang II-induced AAA model and individual data points corresponding to each statistical graph. (ZIP) [file pone.0174821.s009.zip › Supplyment Data/Ang II model pressure/Image of pressure/A6-1.jpg]

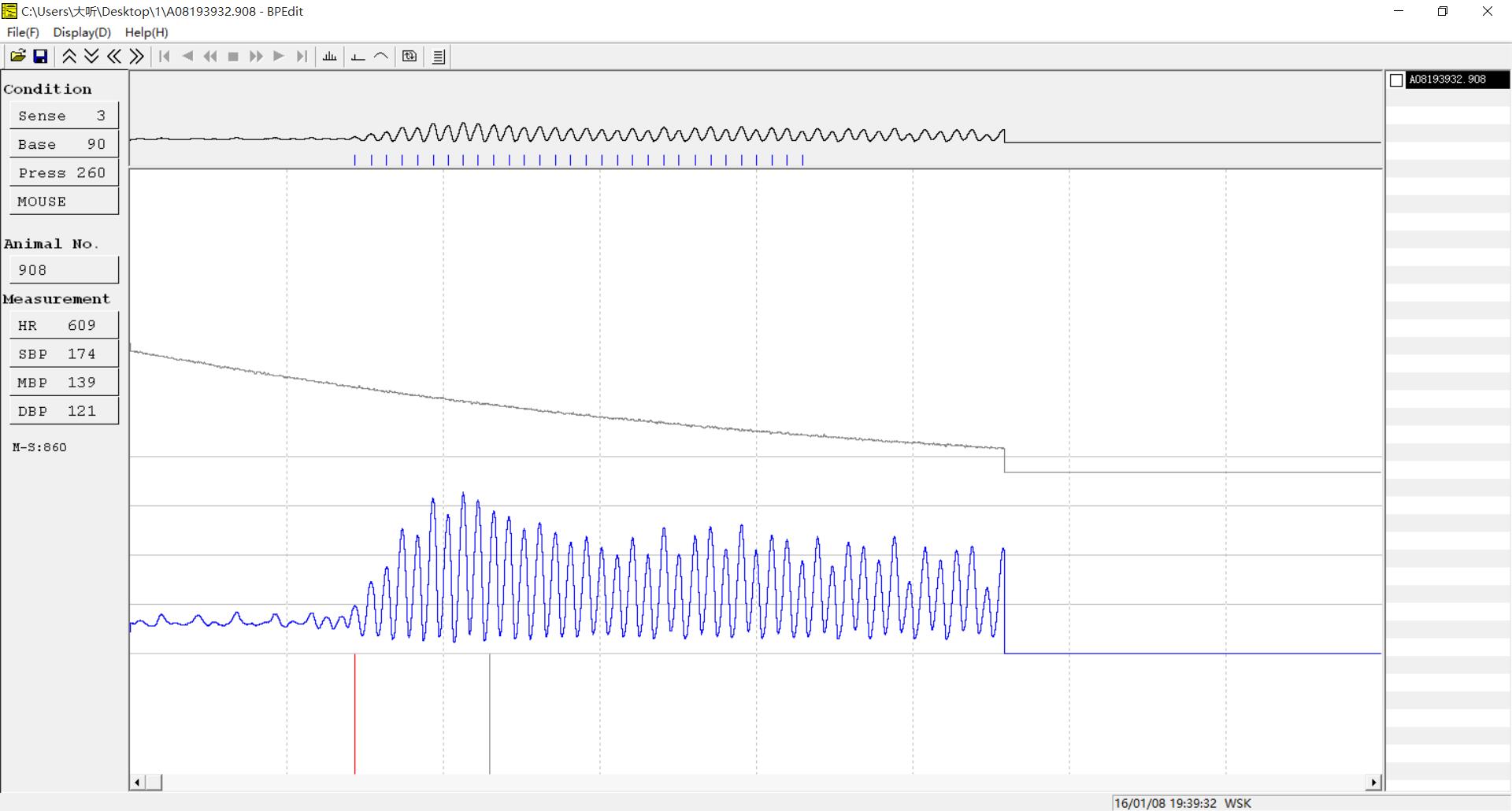

Supplement: S1 File — Pressure data of Ang II-induced AAA model and individual data points corresponding to each statistical graph. (ZIP) [file pone.0174821.s009.zip › Supplyment Data/Ang II model pressure/Image of pressure/A6-2.jpg]

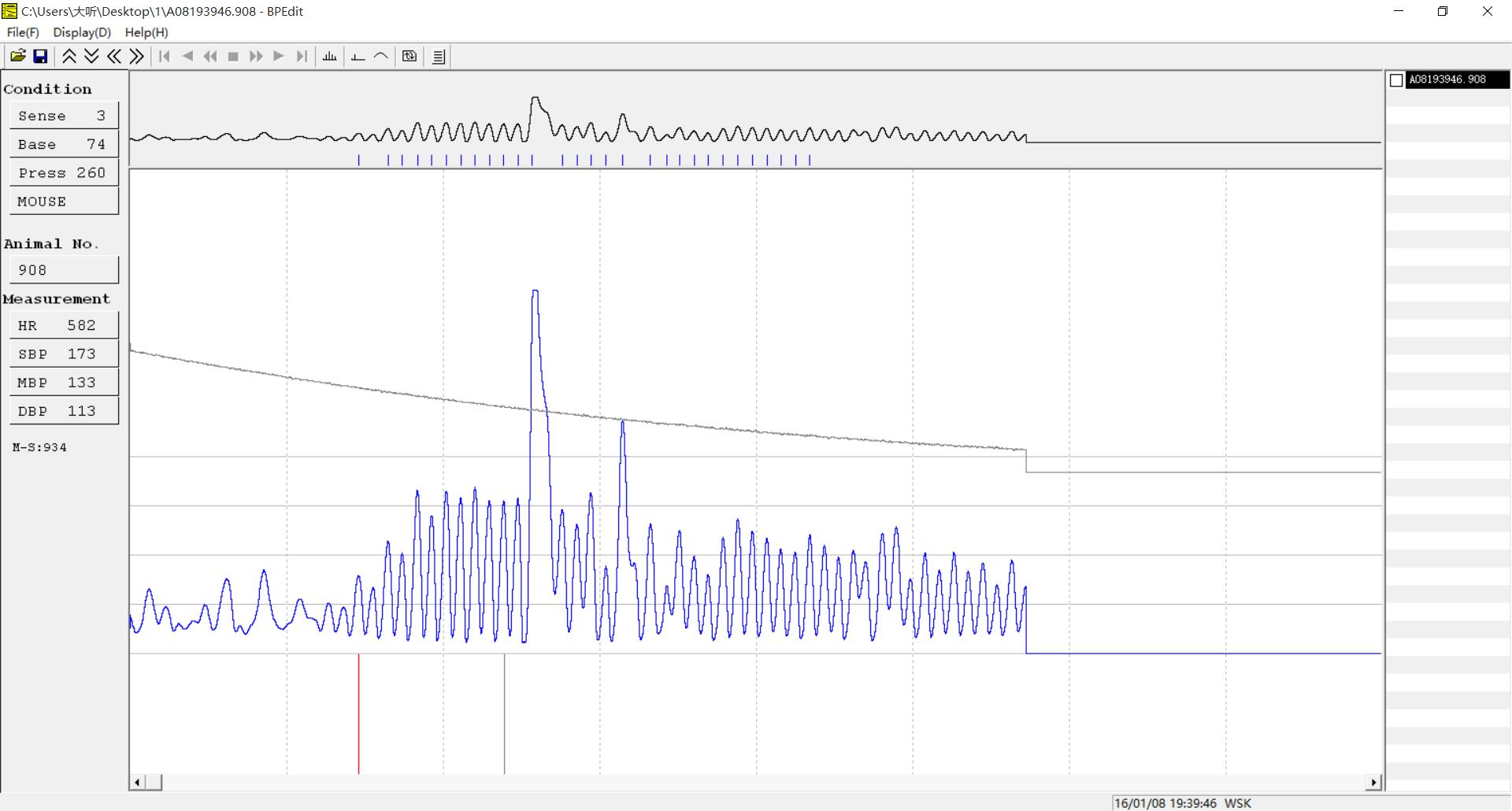

Supplement: S1 File — Pressure data of Ang II-induced AAA model and individual data points corresponding to each statistical graph. (ZIP) [file pone.0174821.s009.zip › Supplyment Data/Ang II model pressure/Image of pressure/A6-3.jpg]

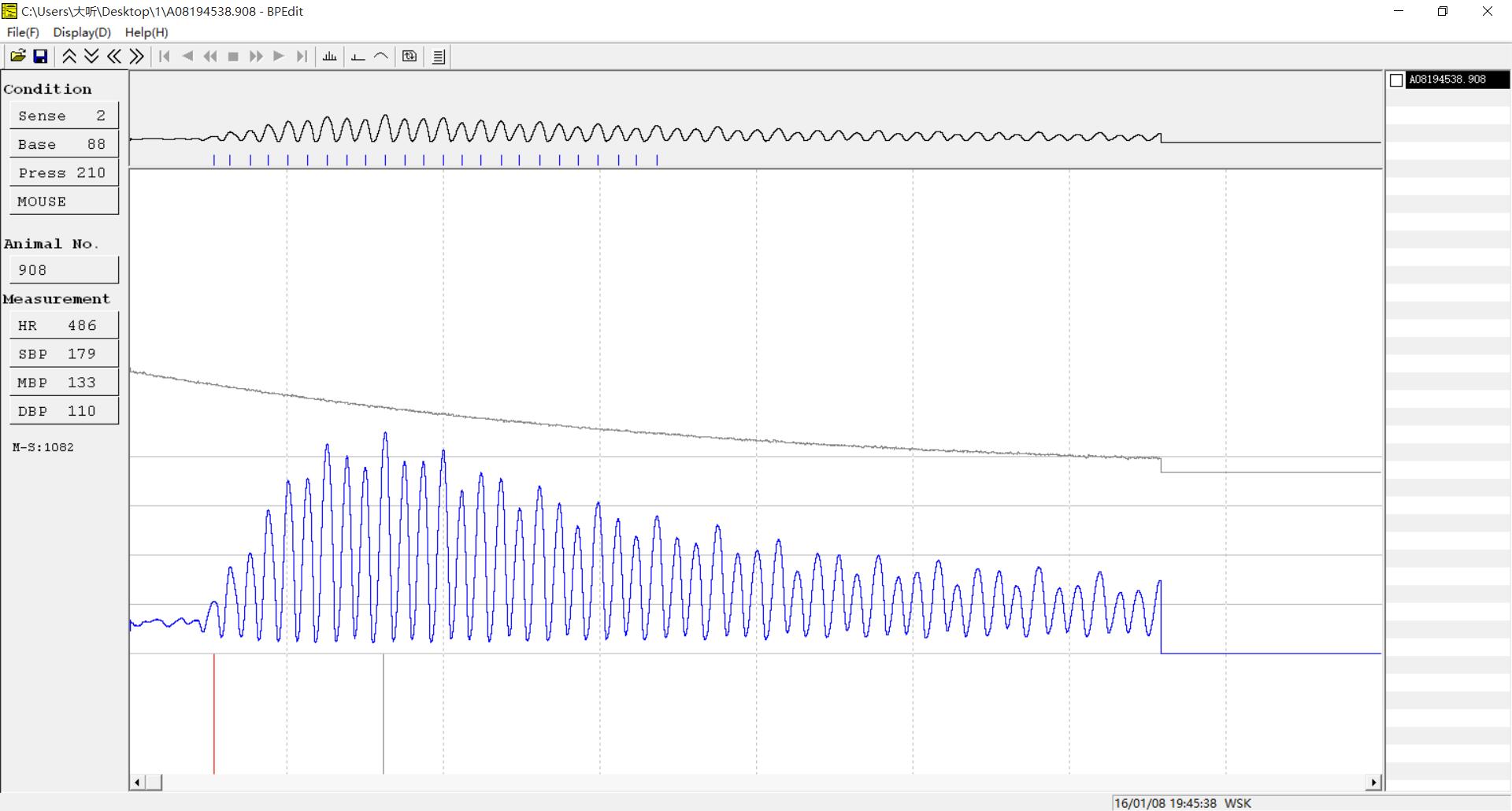

Supplement: S1 File — Pressure data of Ang II-induced AAA model and individual data points corresponding to each statistical graph. (ZIP) [file pone.0174821.s009.zip › Supplyment Data/Ang II model pressure/Image of pressure/A7-1.jpg]

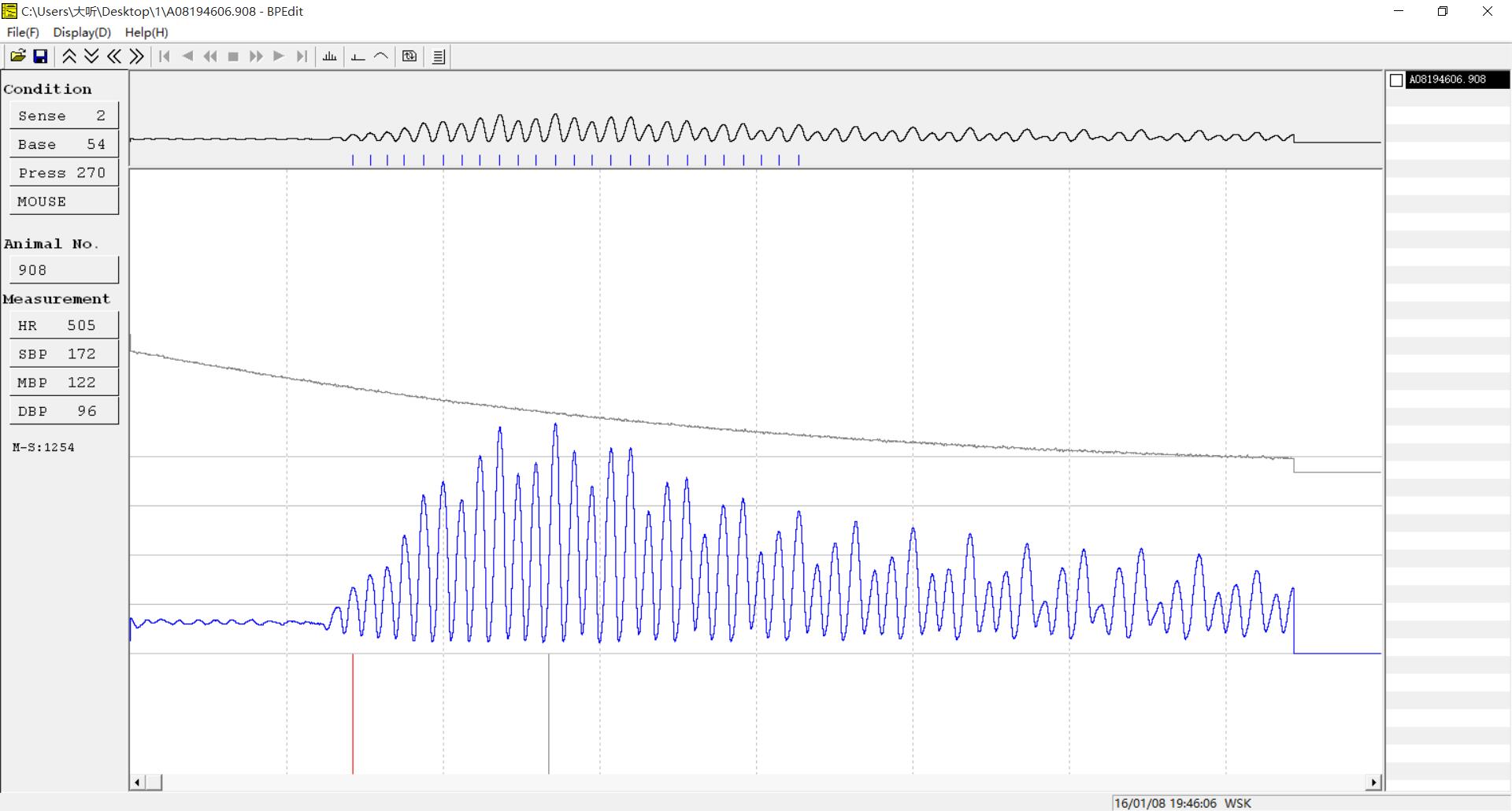

Supplement: S1 File — Pressure data of Ang II-induced AAA model and individual data points corresponding to each statistical graph. (ZIP) [file pone.0174821.s009.zip › Supplyment Data/Ang II model pressure/Image of pressure/A7-2.jpg]

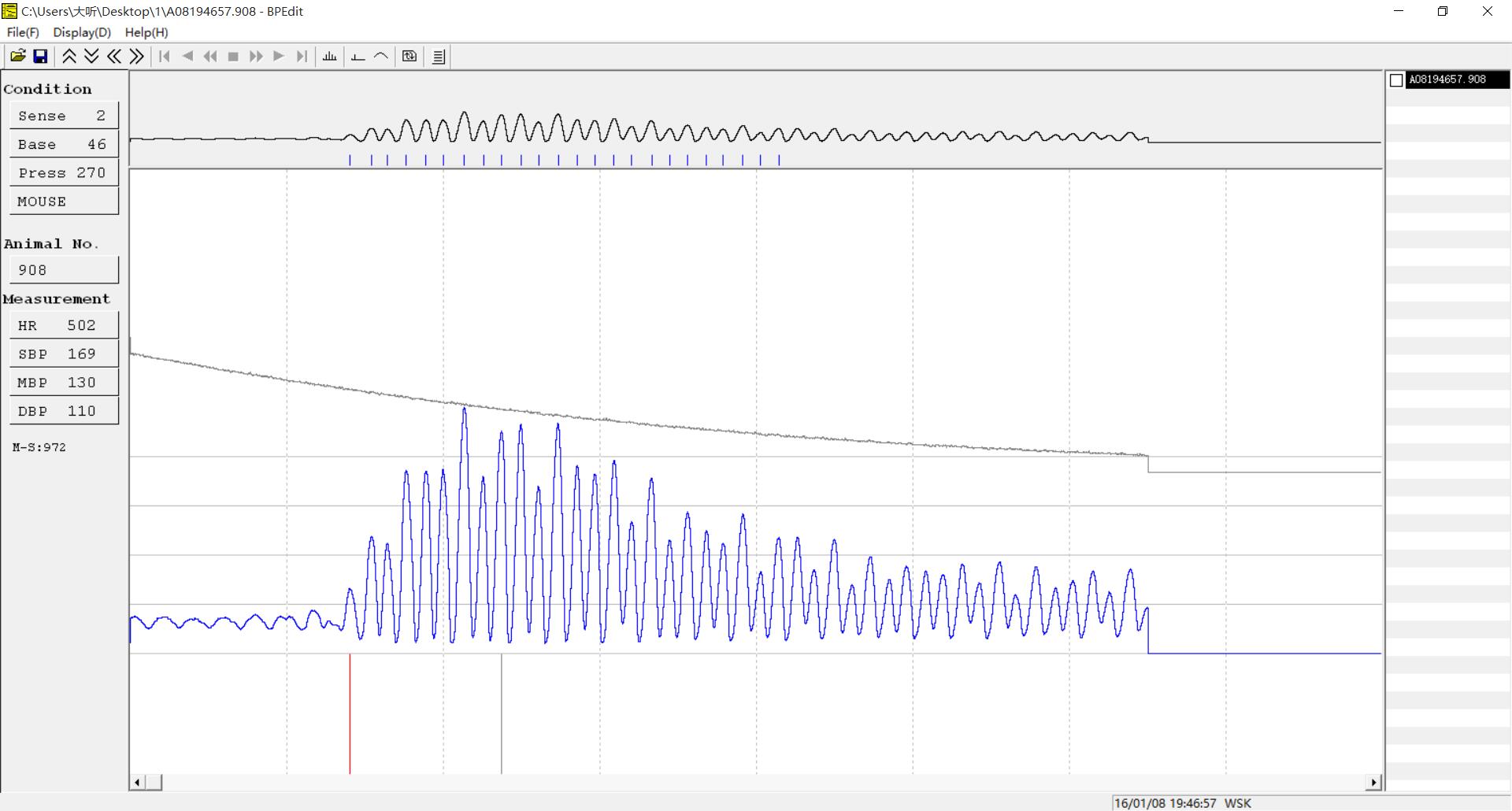

Supplement: S1 File — Pressure data of Ang II-induced AAA model and individual data points corresponding to each statistical graph. (ZIP) [file pone.0174821.s009.zip › Supplyment Data/Ang II model pressure/Image of pressure/A7-3.jpg]

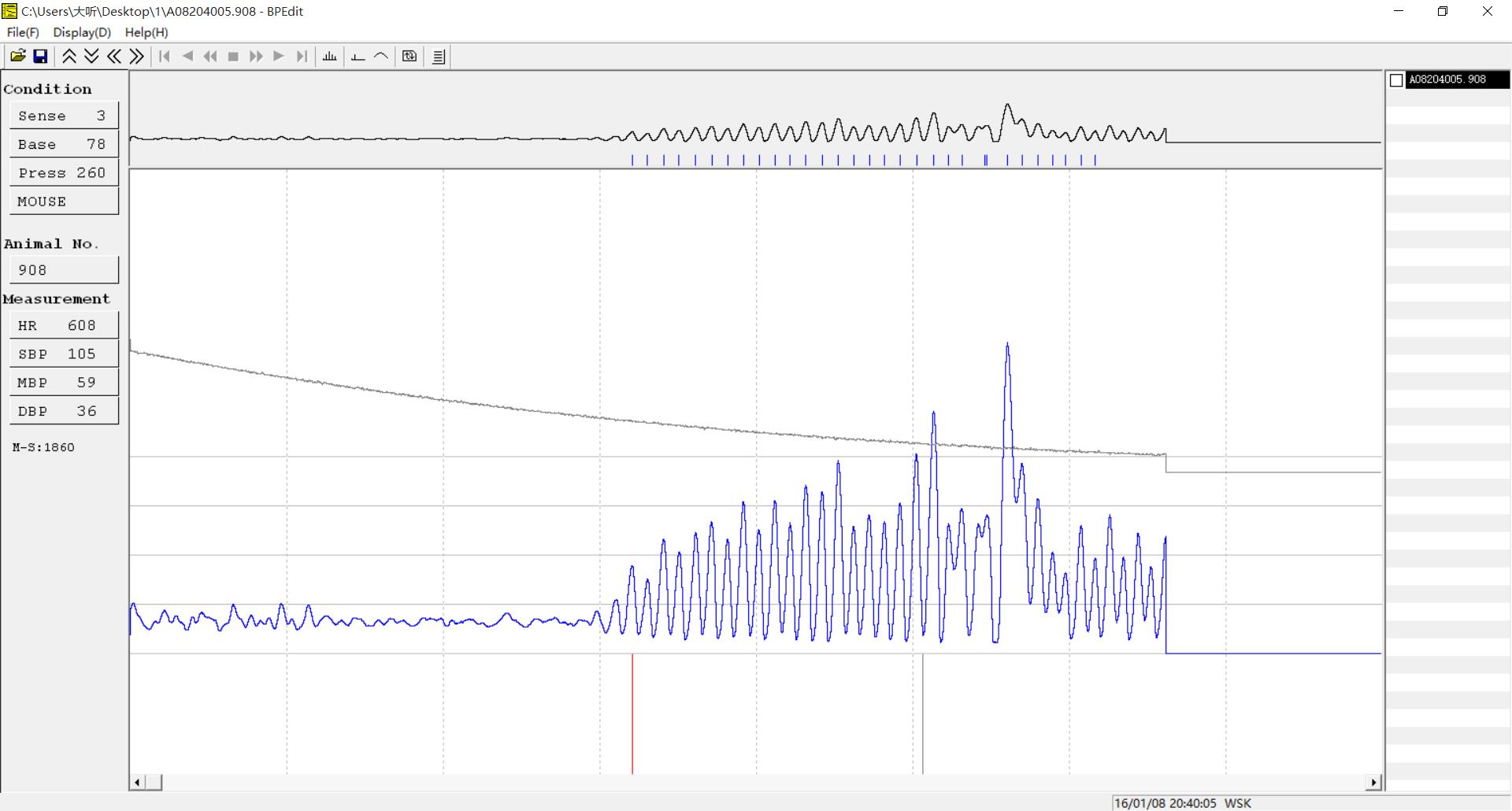

Supplement: S1 File — Pressure data of Ang II-induced AAA model and individual data points corresponding to each statistical graph. (ZIP) [file pone.0174821.s009.zip › Supplyment Data/Ang II model pressure/Image of pressure/P1-1.jpg]

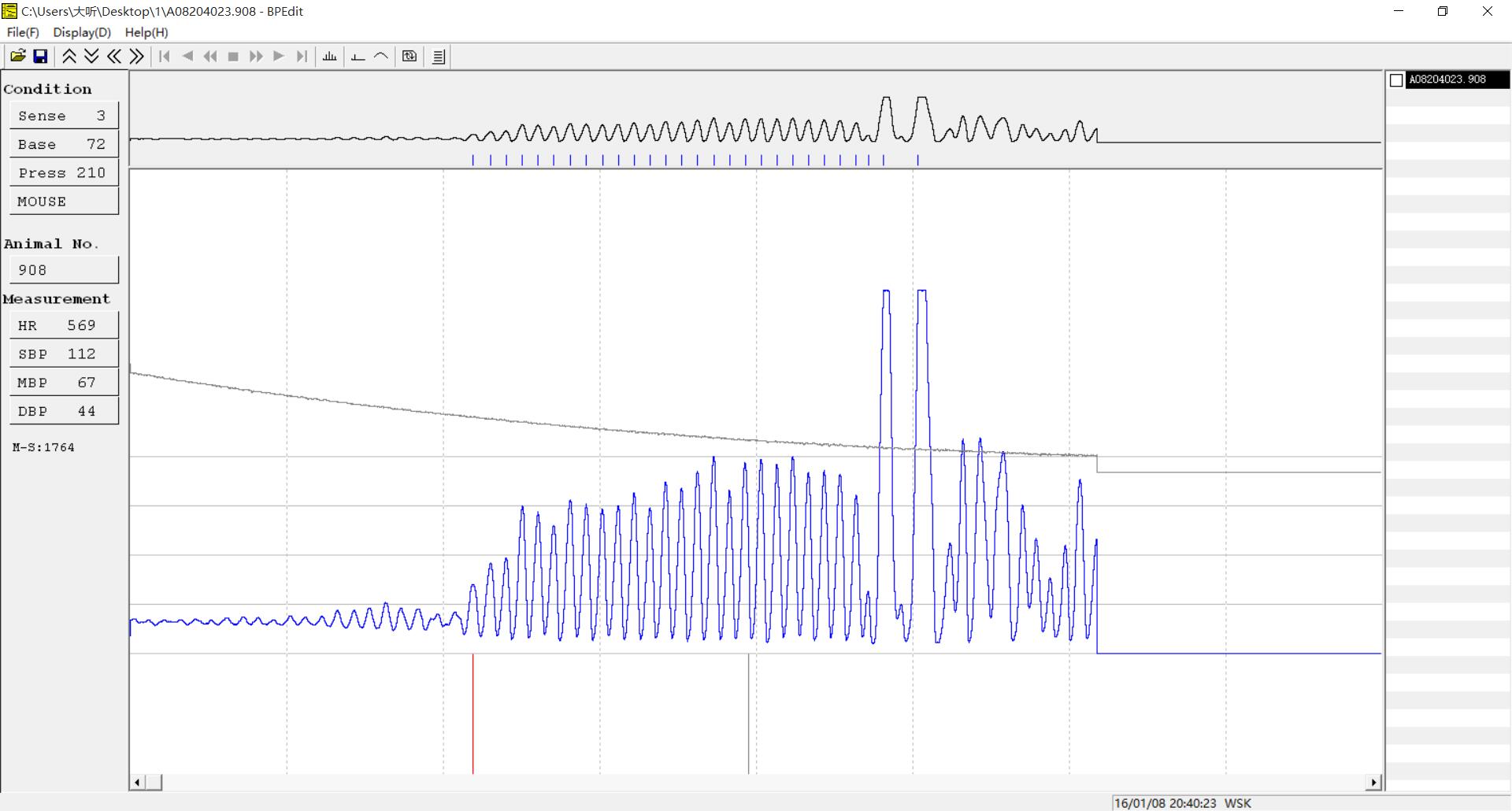

Supplement: S1 File — Pressure data of Ang II-induced AAA model and individual data points corresponding to each statistical graph. (ZIP) [file pone.0174821.s009.zip › Supplyment Data/Ang II model pressure/Image of pressure/P1-2.jpg]

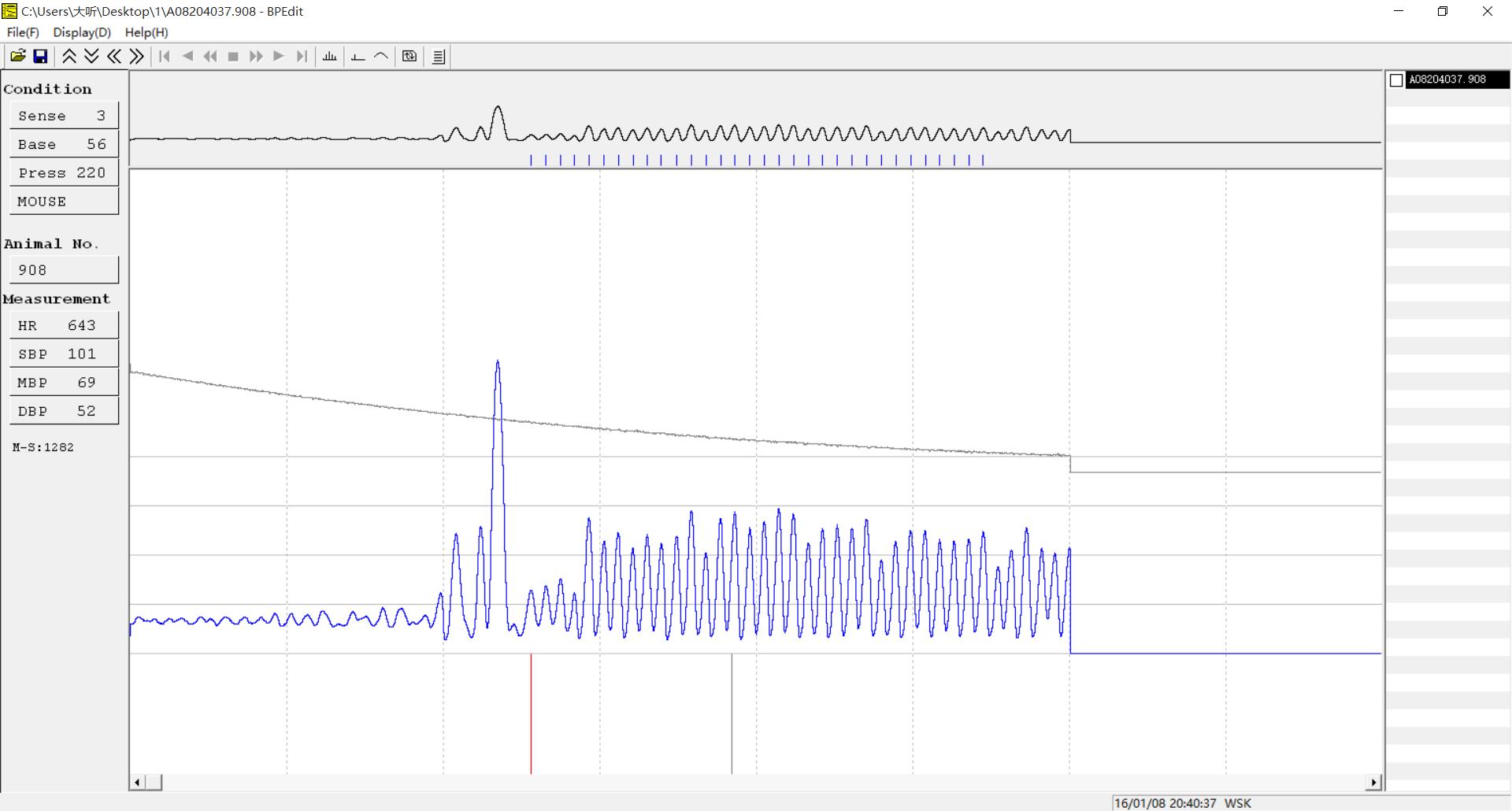

Supplement: S1 File — Pressure data of Ang II-induced AAA model and individual data points corresponding to each statistical graph. (ZIP) [file pone.0174821.s009.zip › Supplyment Data/Ang II model pressure/Image of pressure/P1-3.jpg]

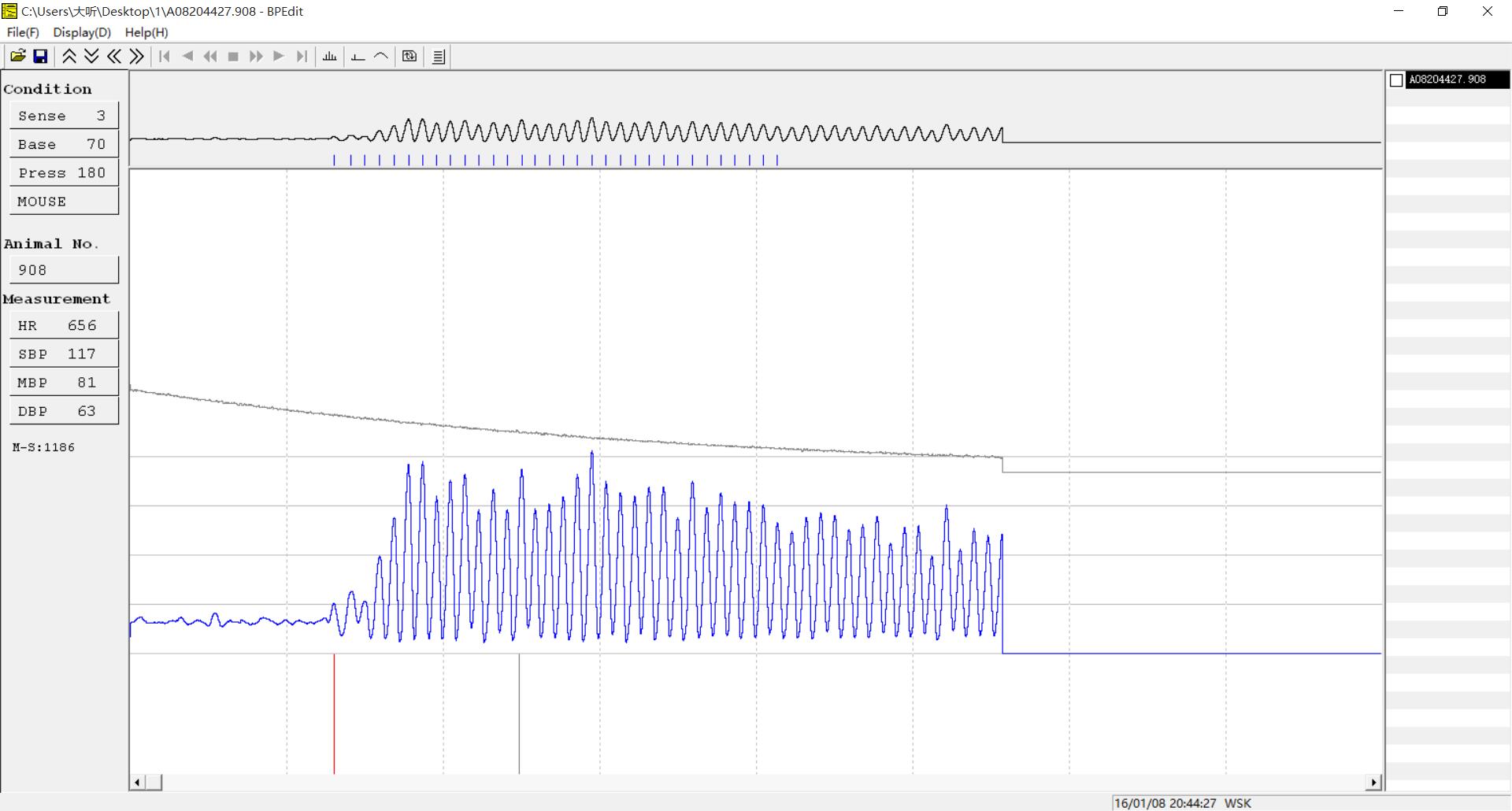

Supplement: S1 File — Pressure data of Ang II-induced AAA model and individual data points corresponding to each statistical graph. (ZIP) [file pone.0174821.s009.zip › Supplyment Data/Ang II model pressure/Image of pressure/P2-1.jpg]

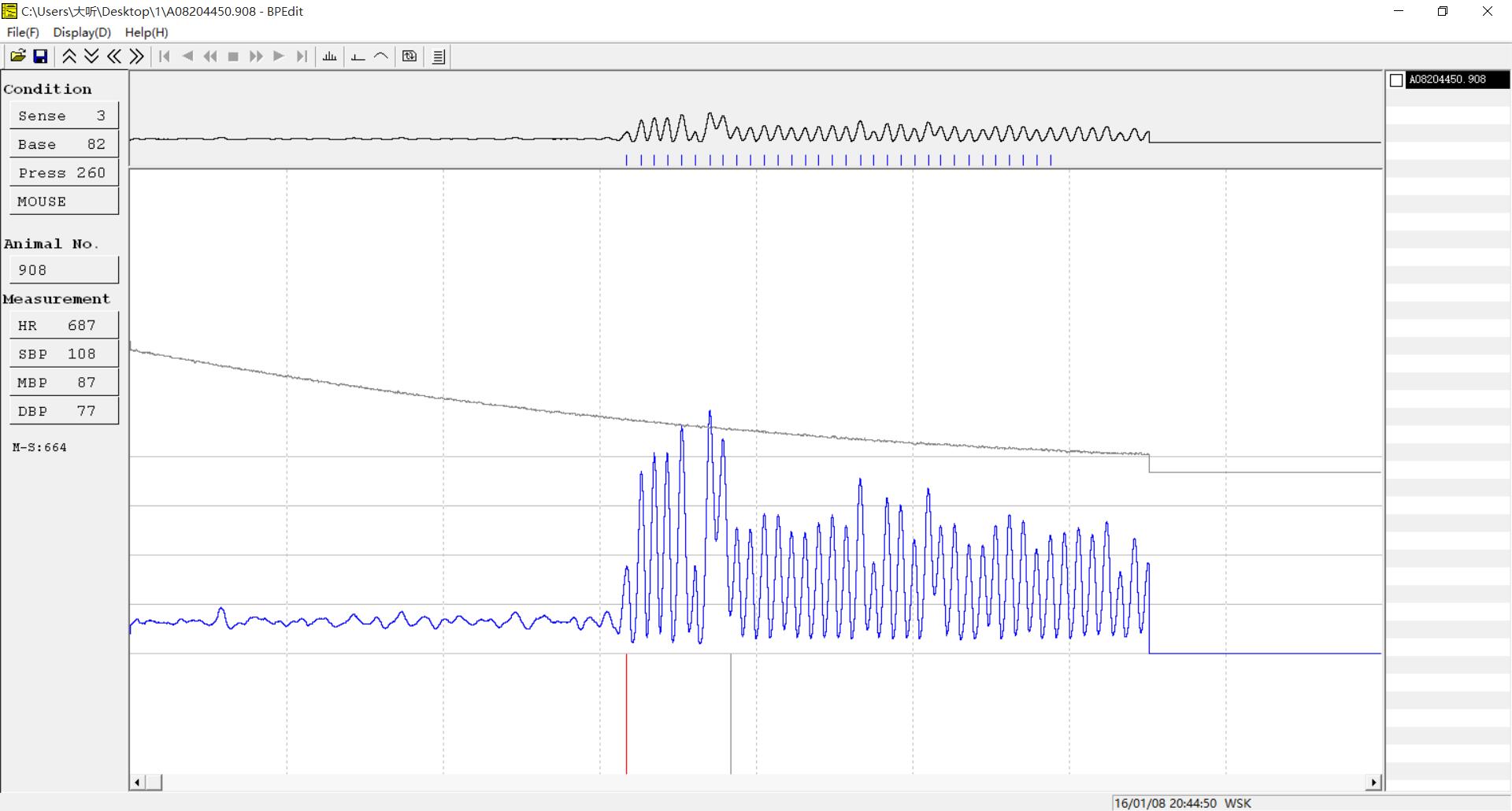

Supplement: S1 File — Pressure data of Ang II-induced AAA model and individual data points corresponding to each statistical graph. (ZIP) [file pone.0174821.s009.zip › Supplyment Data/Ang II model pressure/Image of pressure/P2-2.jpg]

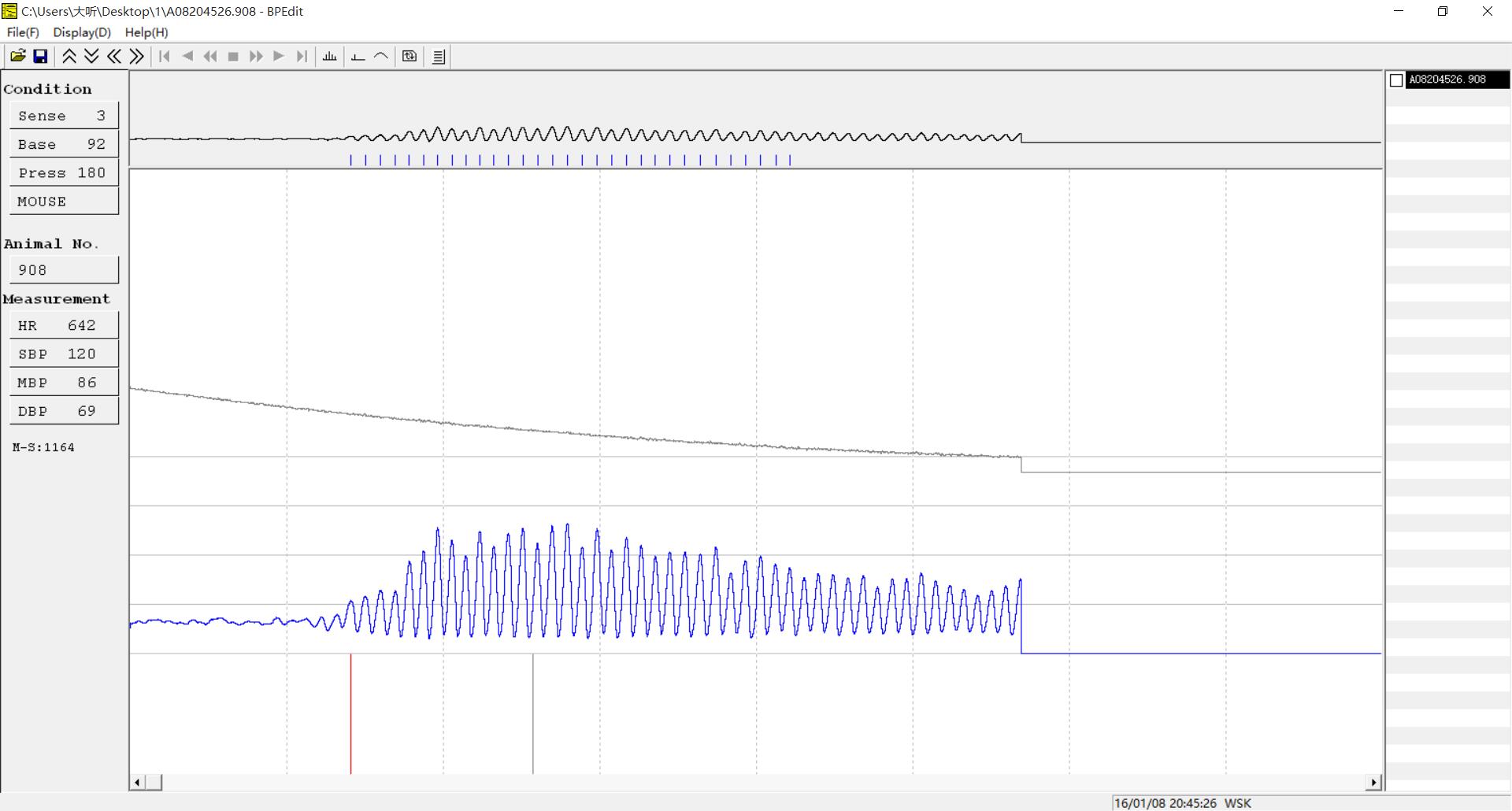

Supplement: S1 File — Pressure data of Ang II-induced AAA model and individual data points corresponding to each statistical graph. (ZIP) [file pone.0174821.s009.zip › Supplyment Data/Ang II model pressure/Image of pressure/P2-3.jpg]

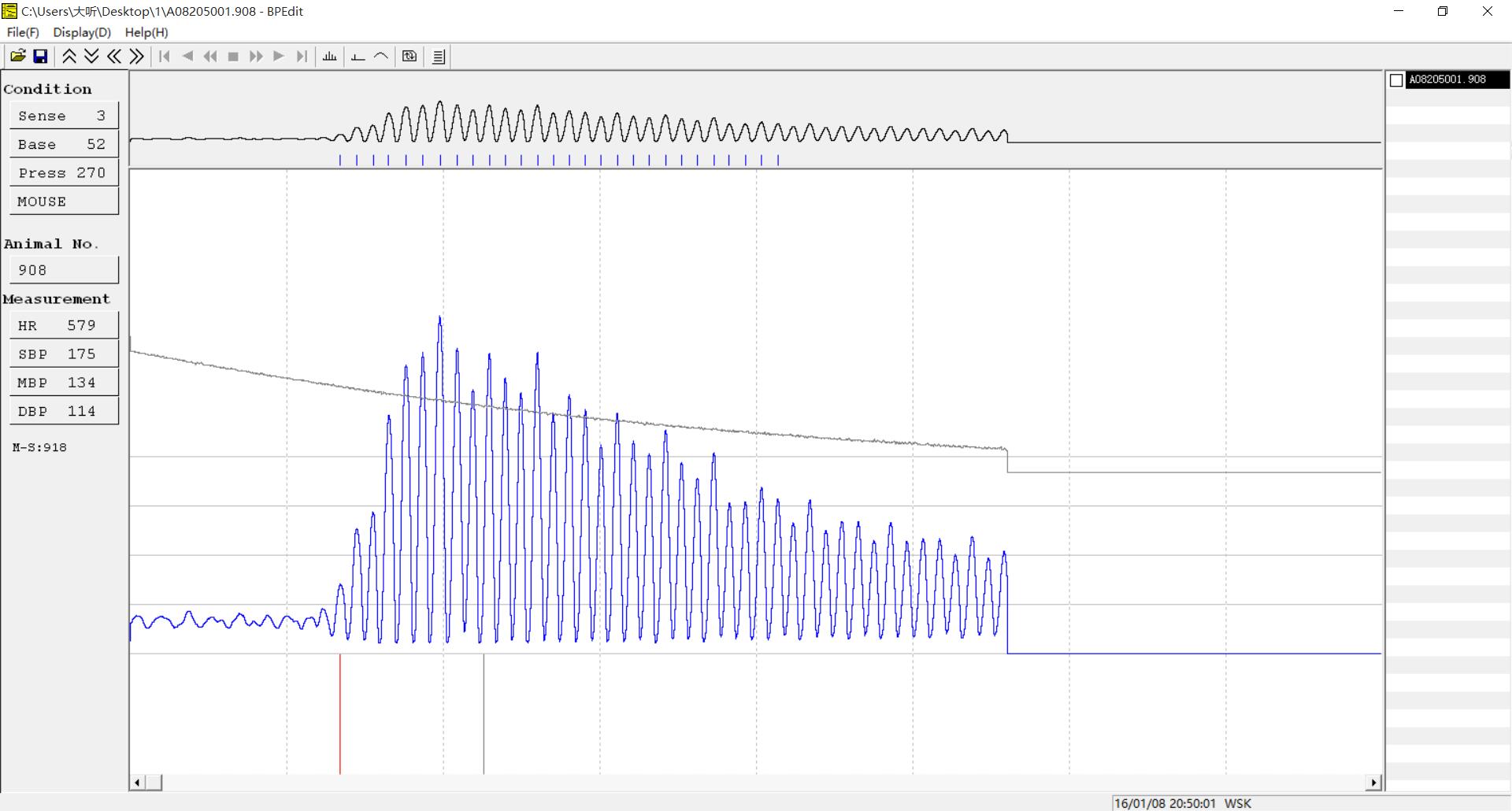

Supplement: S1 File — Pressure data of Ang II-induced AAA model and individual data points corresponding to each statistical graph. (ZIP) [file pone.0174821.s009.zip › Supplyment Data/Ang II model pressure/Image of pressure/P4-1.jpg]

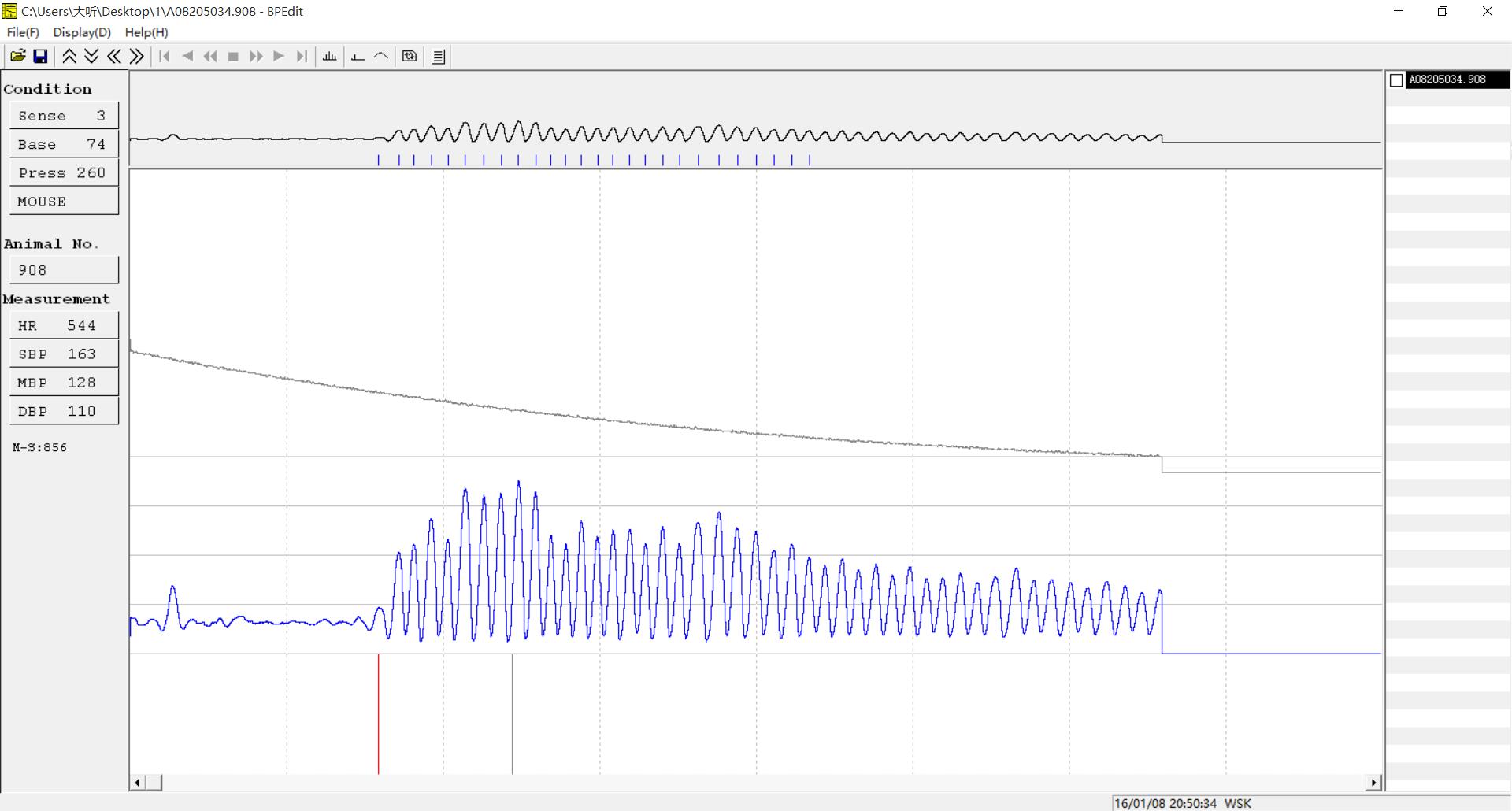

Supplement: S1 File — Pressure data of Ang II-induced AAA model and individual data points corresponding to each statistical graph. (ZIP) [file pone.0174821.s009.zip › Supplyment Data/Ang II model pressure/Image of pressure/P4-2.jpg]

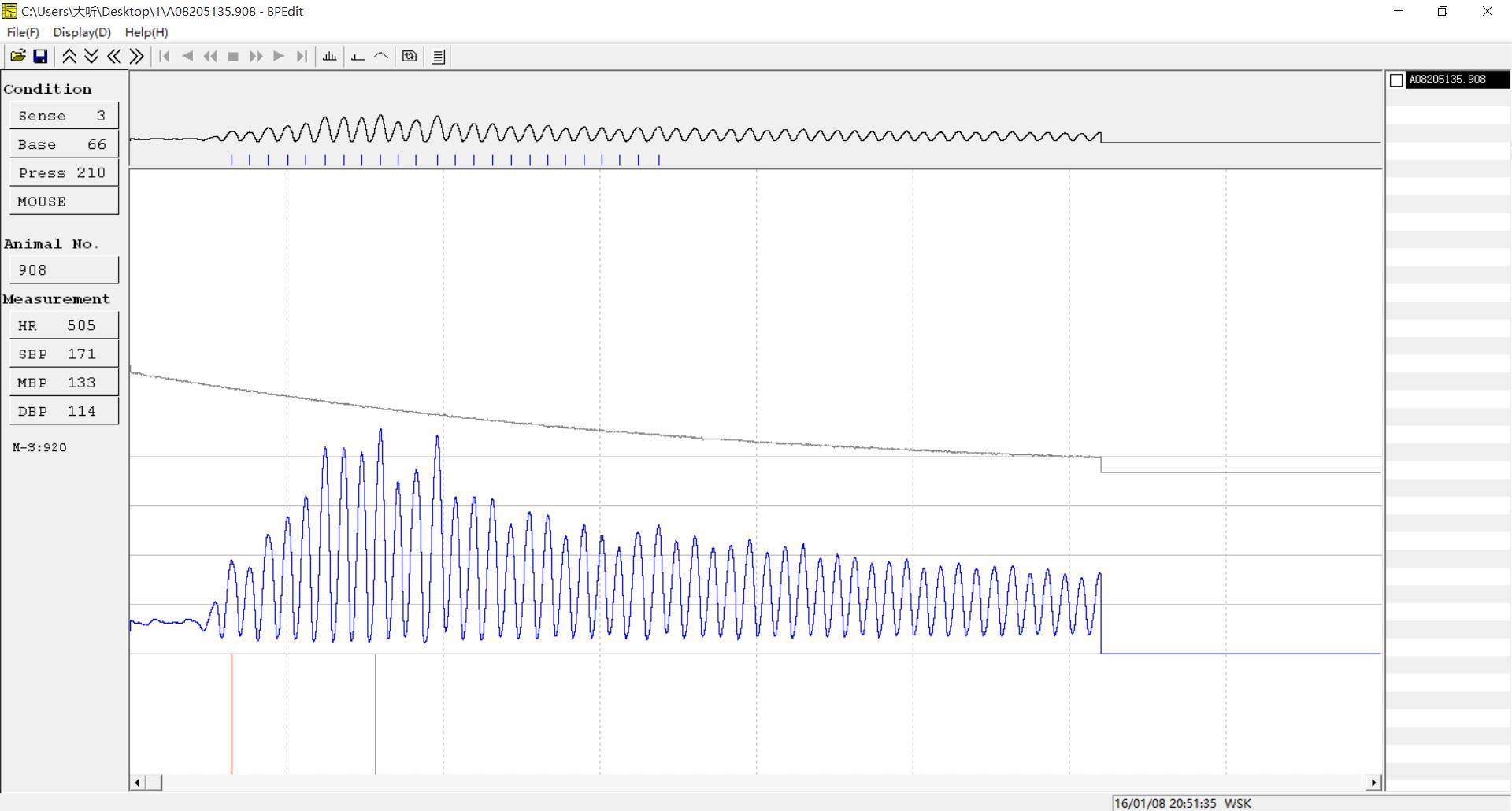

Supplement: S1 File — Pressure data of Ang II-induced AAA model and individual data points corresponding to each statistical graph. (ZIP) [file pone.0174821.s009.zip › Supplyment Data/Ang II model pressure/Image of pressure/P4-3.jpg]

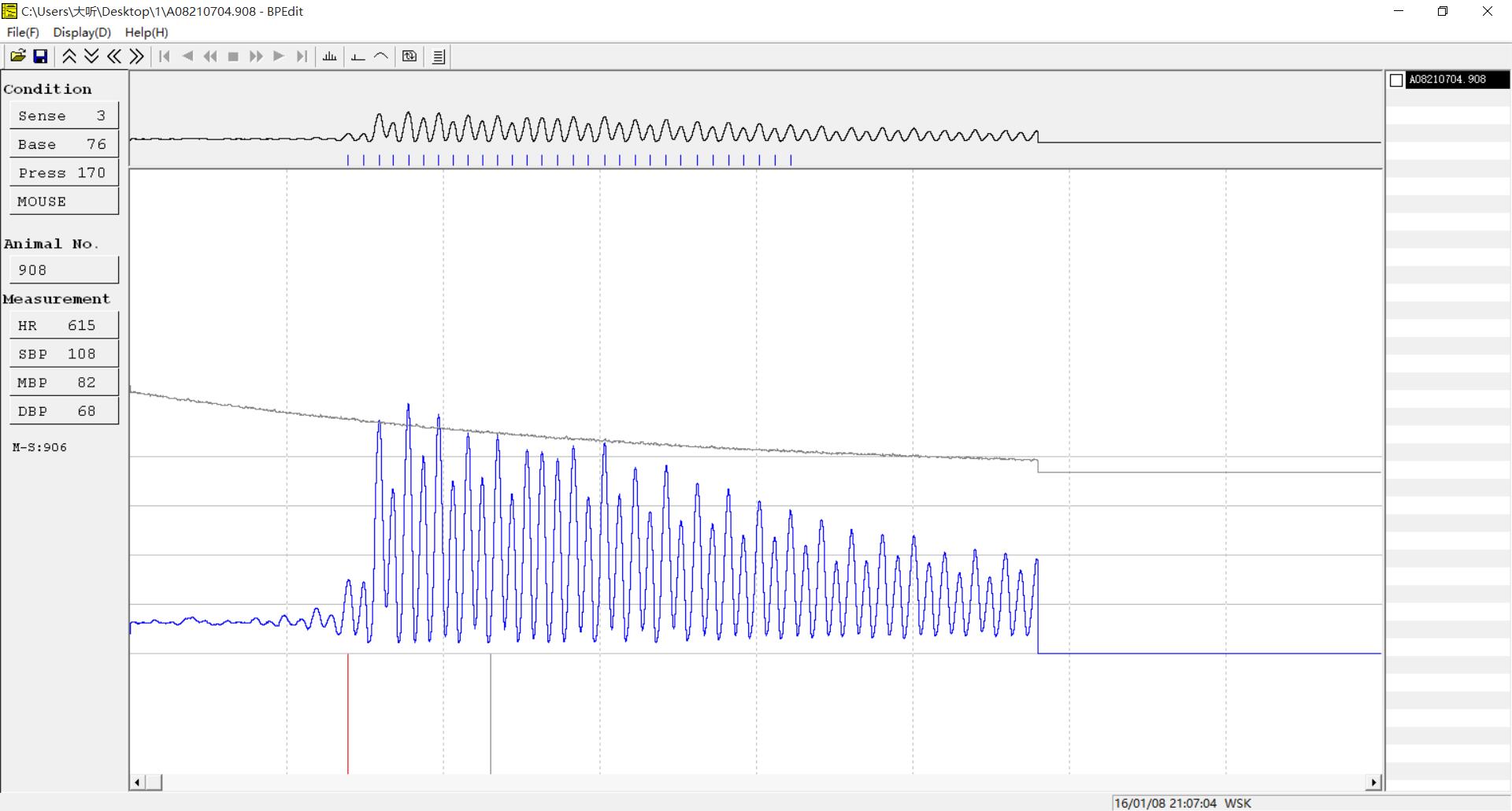

Supplement: S1 File — Pressure data of Ang II-induced AAA model and individual data points corresponding to each statistical graph. (ZIP) [file pone.0174821.s009.zip › Supplyment Data/Ang II model pressure/Image of pressure/P5-1.jpg]

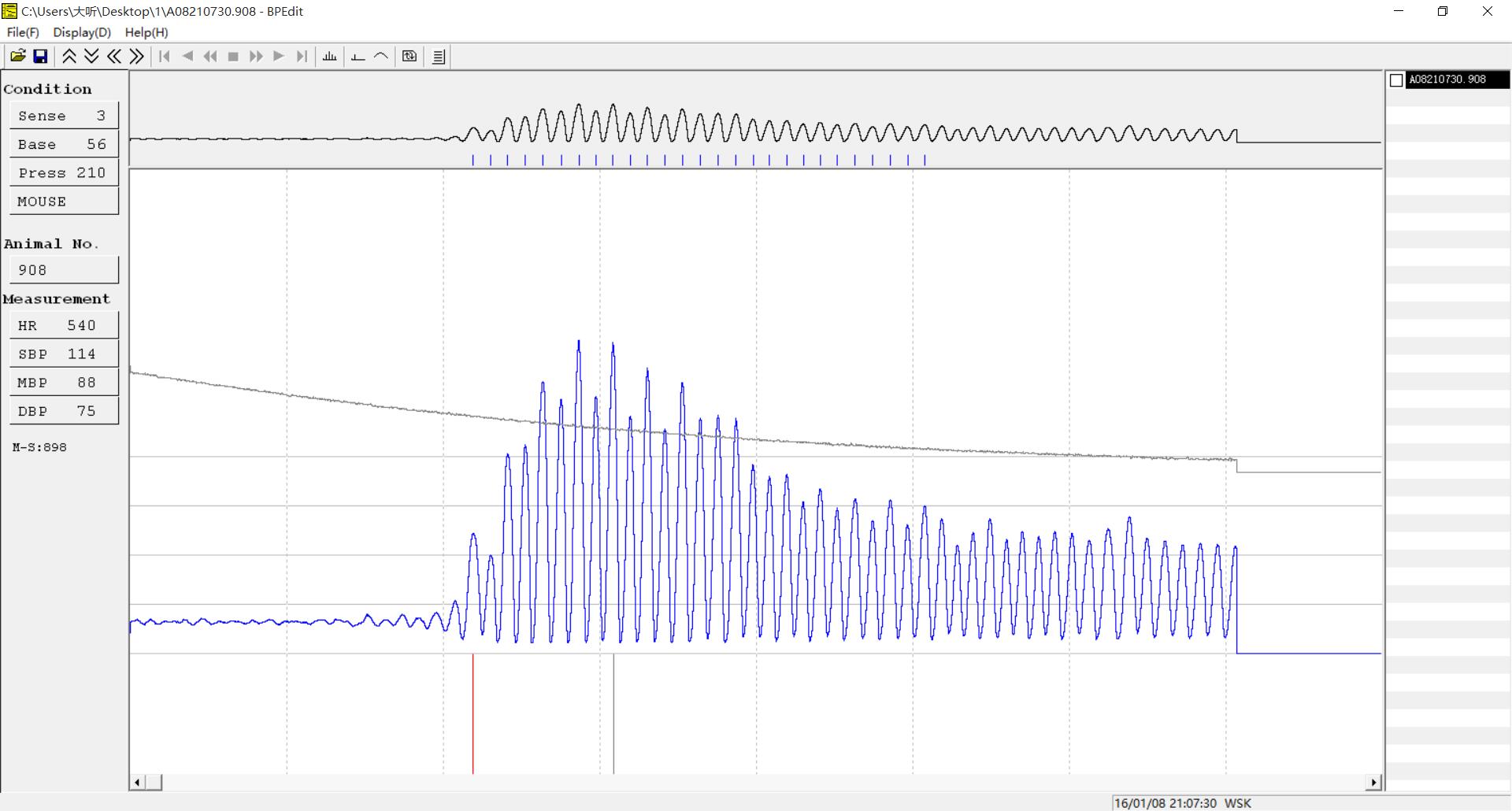

Supplement: S1 File — Pressure data of Ang II-induced AAA model and individual data points corresponding to each statistical graph. (ZIP) [file pone.0174821.s009.zip › Supplyment Data/Ang II model pressure/Image of pressure/P5-2.jpg]

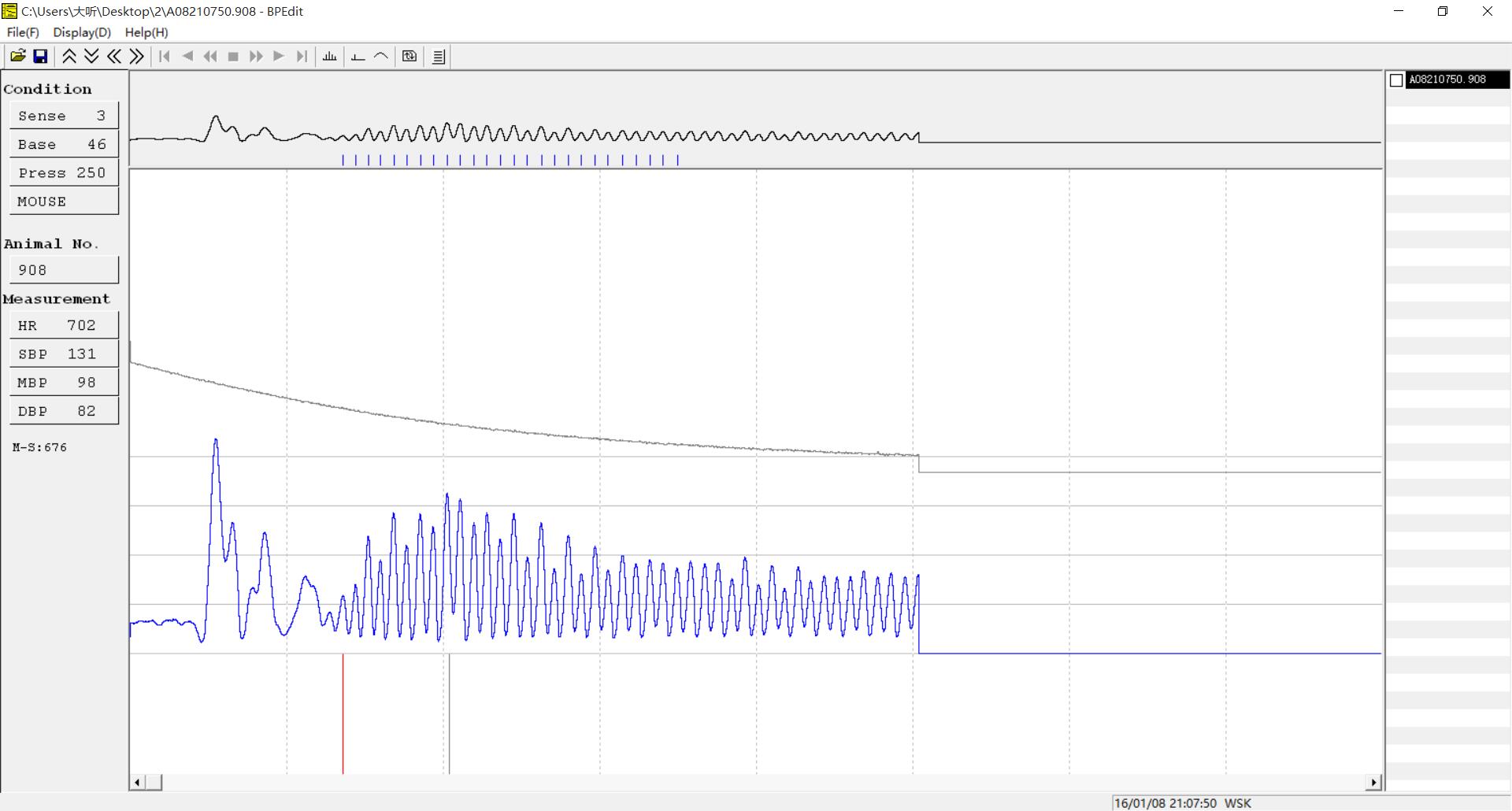

Supplement: S1 File — Pressure data of Ang II-induced AAA model and individual data points corresponding to each statistical graph. (ZIP) [file pone.0174821.s009.zip › Supplyment Data/Ang II model pressure/Image of pressure/P5-3.jpg]

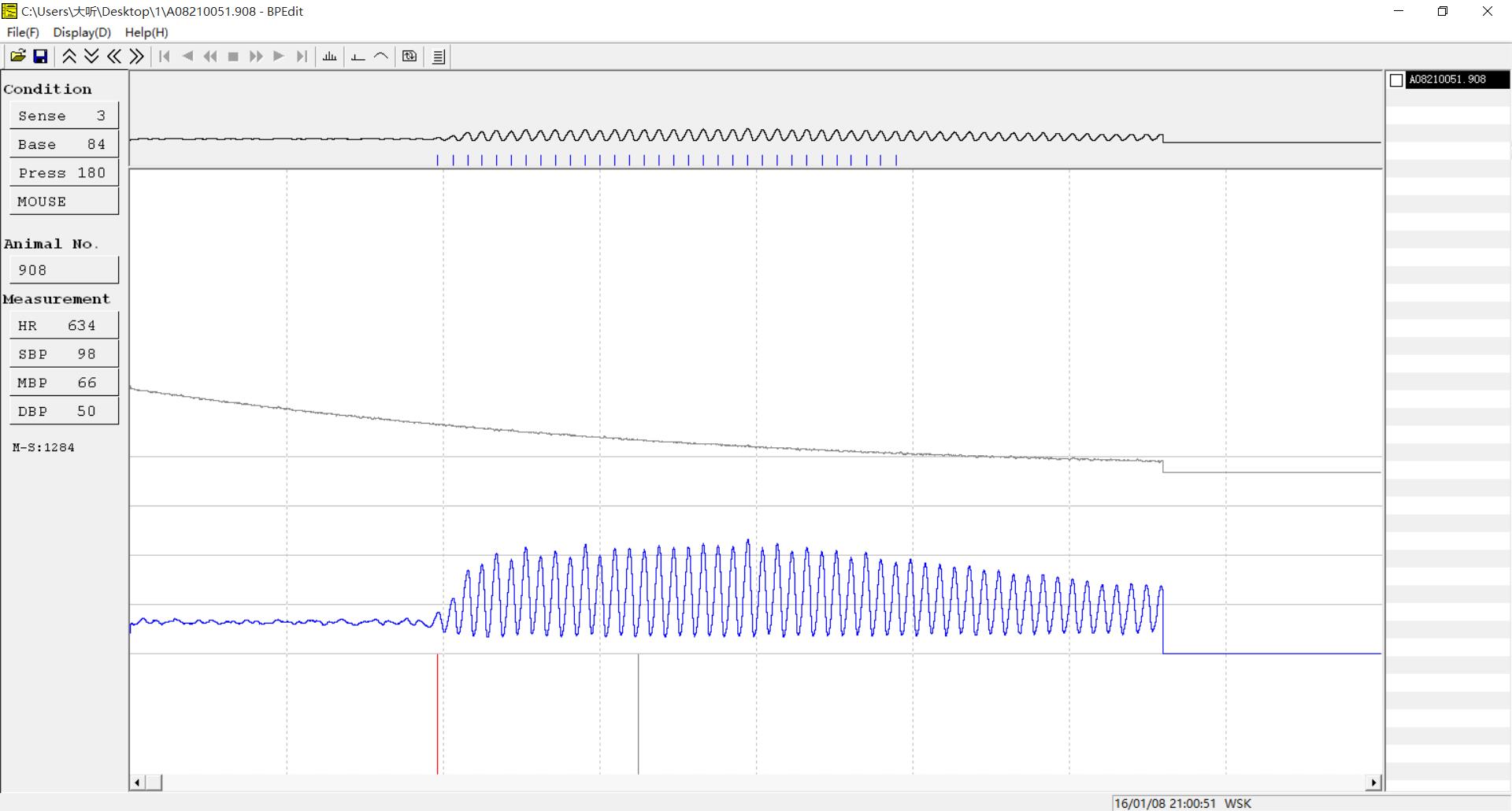

Supplement: S1 File — Pressure data of Ang II-induced AAA model and individual data points corresponding to each statistical graph. (ZIP) [file pone.0174821.s009.zip › Supplyment Data/Ang II model pressure/Image of pressure/P6-1.jpg]

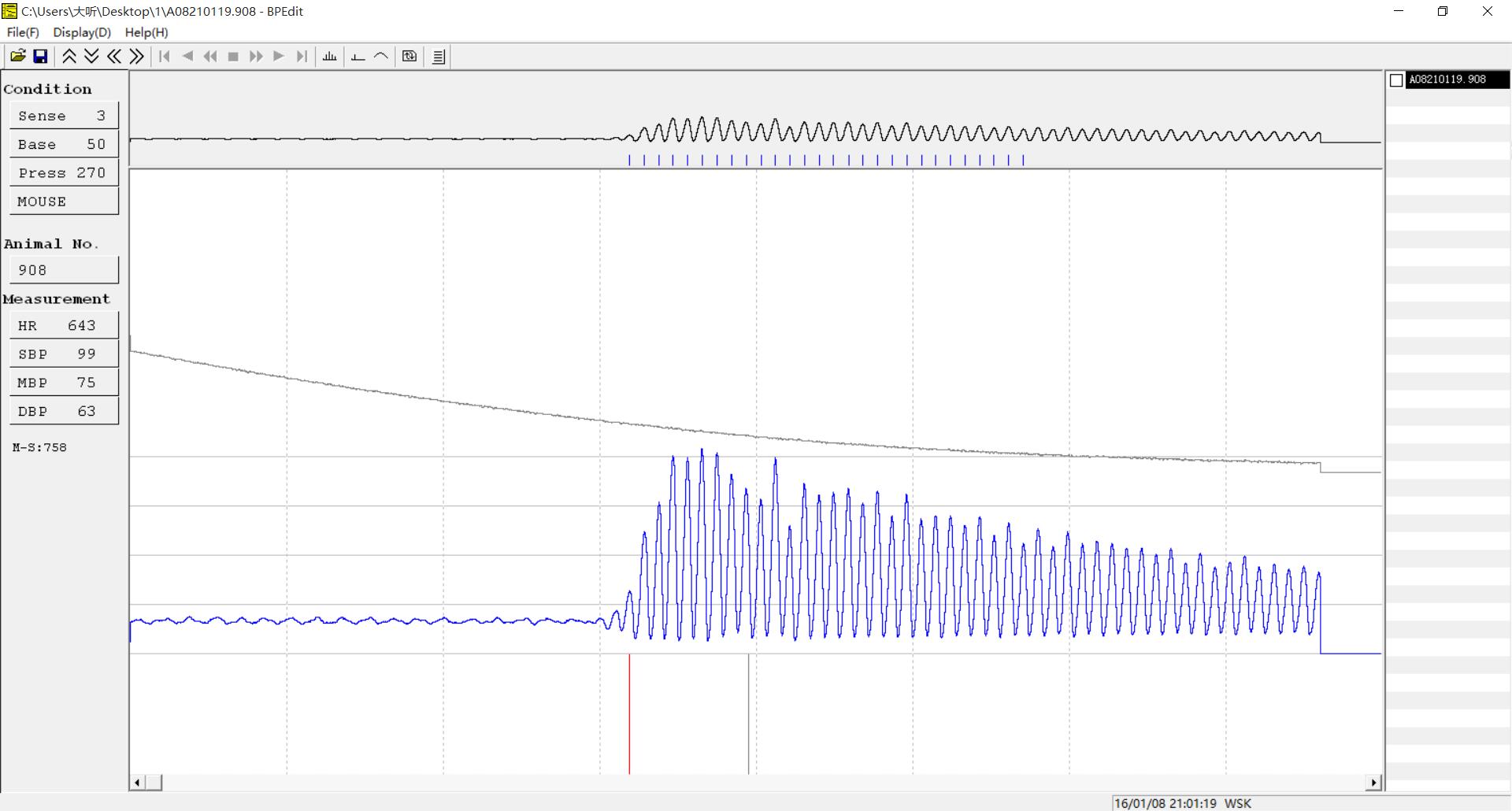

Supplement: S1 File — Pressure data of Ang II-induced AAA model and individual data points corresponding to each statistical graph. (ZIP) [file pone.0174821.s009.zip › Supplyment Data/Ang II model pressure/Image of pressure/P6-2.jpg]

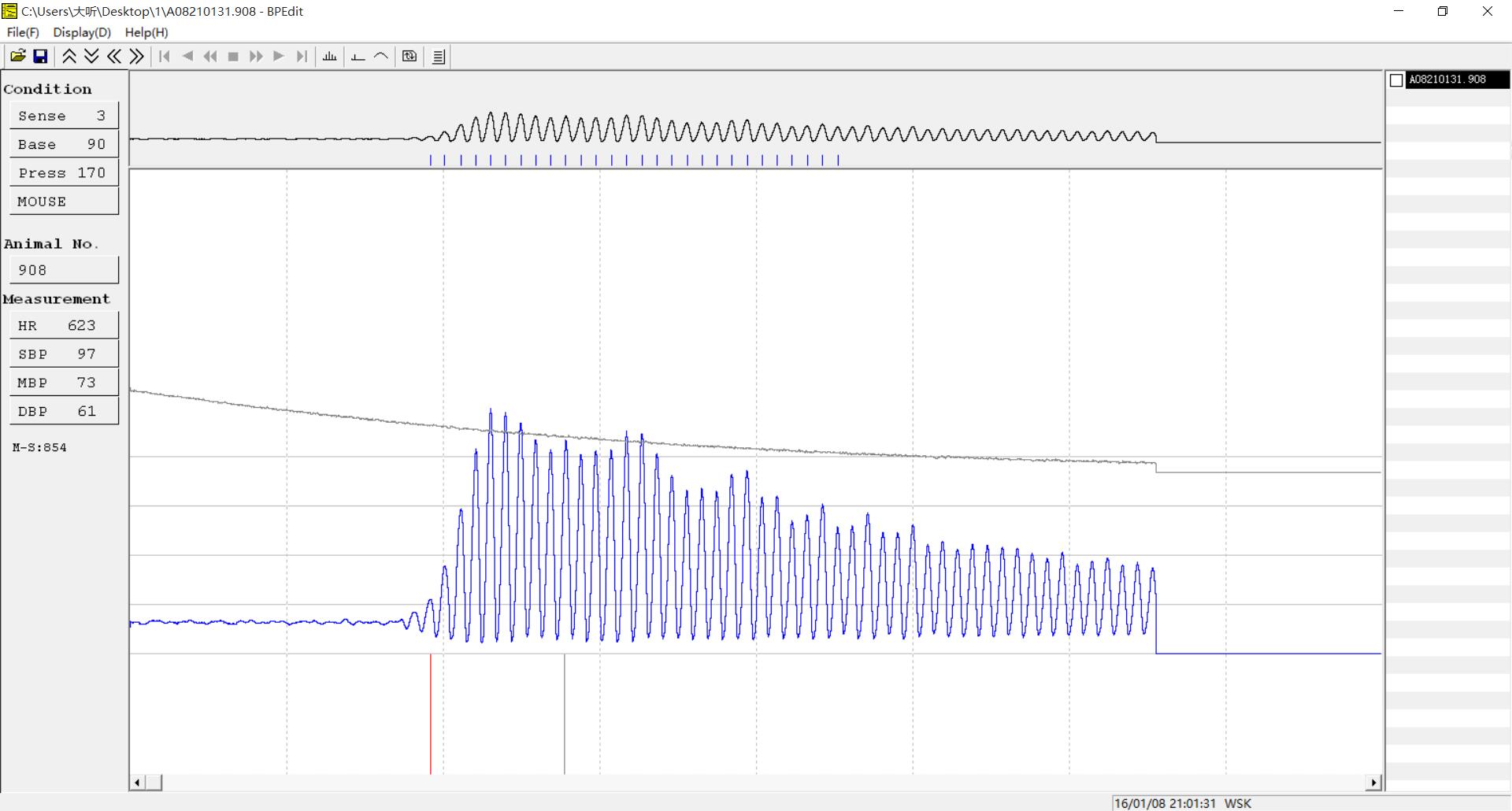

Supplement: S1 File — Pressure data of Ang II-induced AAA model and individual data points corresponding to each statistical graph. (ZIP) [file pone.0174821.s009.zip › Supplyment Data/Ang II model pressure/Image of pressure/P6-3.jpg]

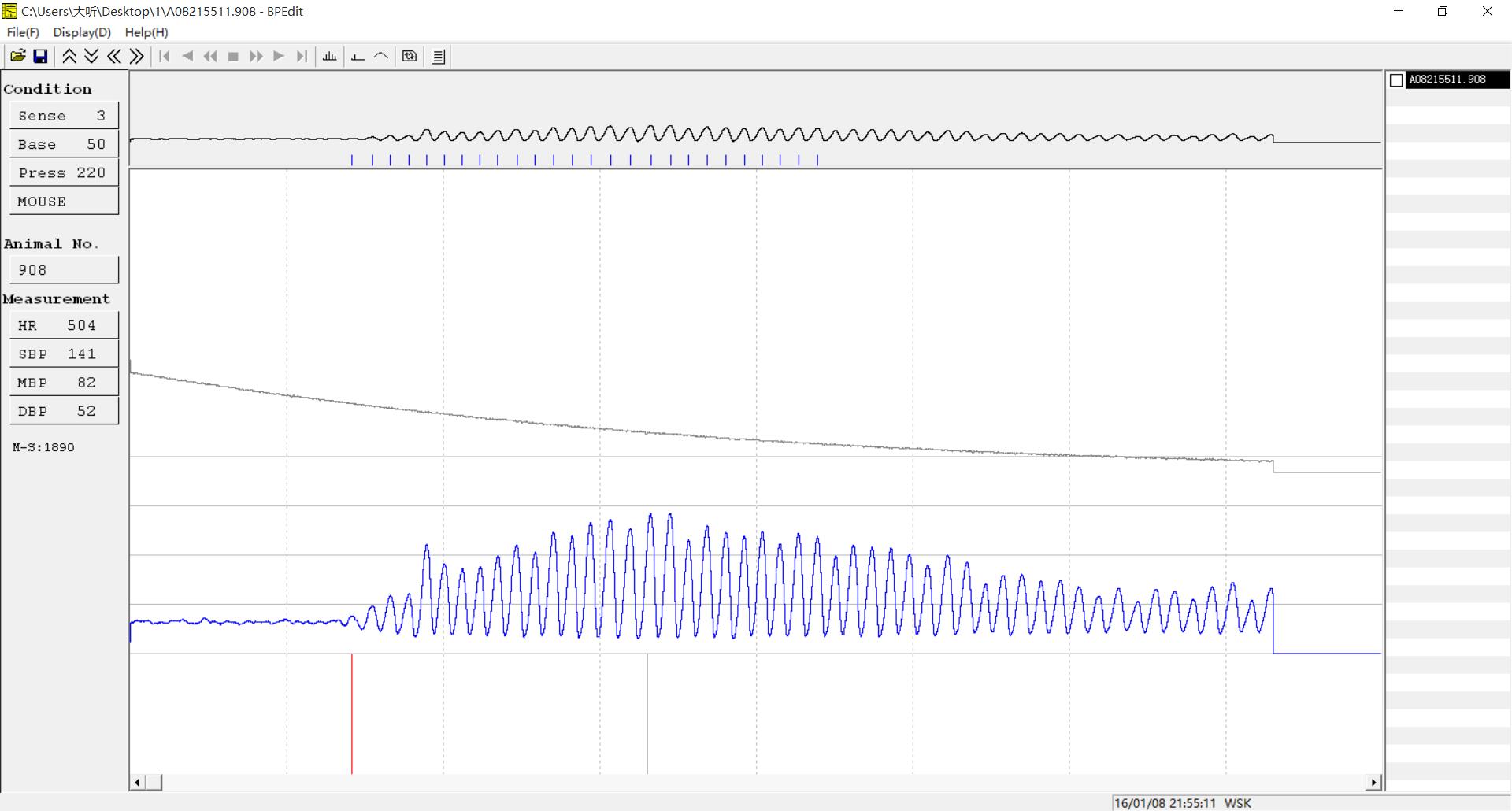

Supplement: S1 File — Pressure data of Ang II-induced AAA model and individual data points corresponding to each statistical graph. (ZIP) [file pone.0174821.s009.zip › Supplyment Data/Ang II model pressure/Image of pressure/S1-1.jpg]

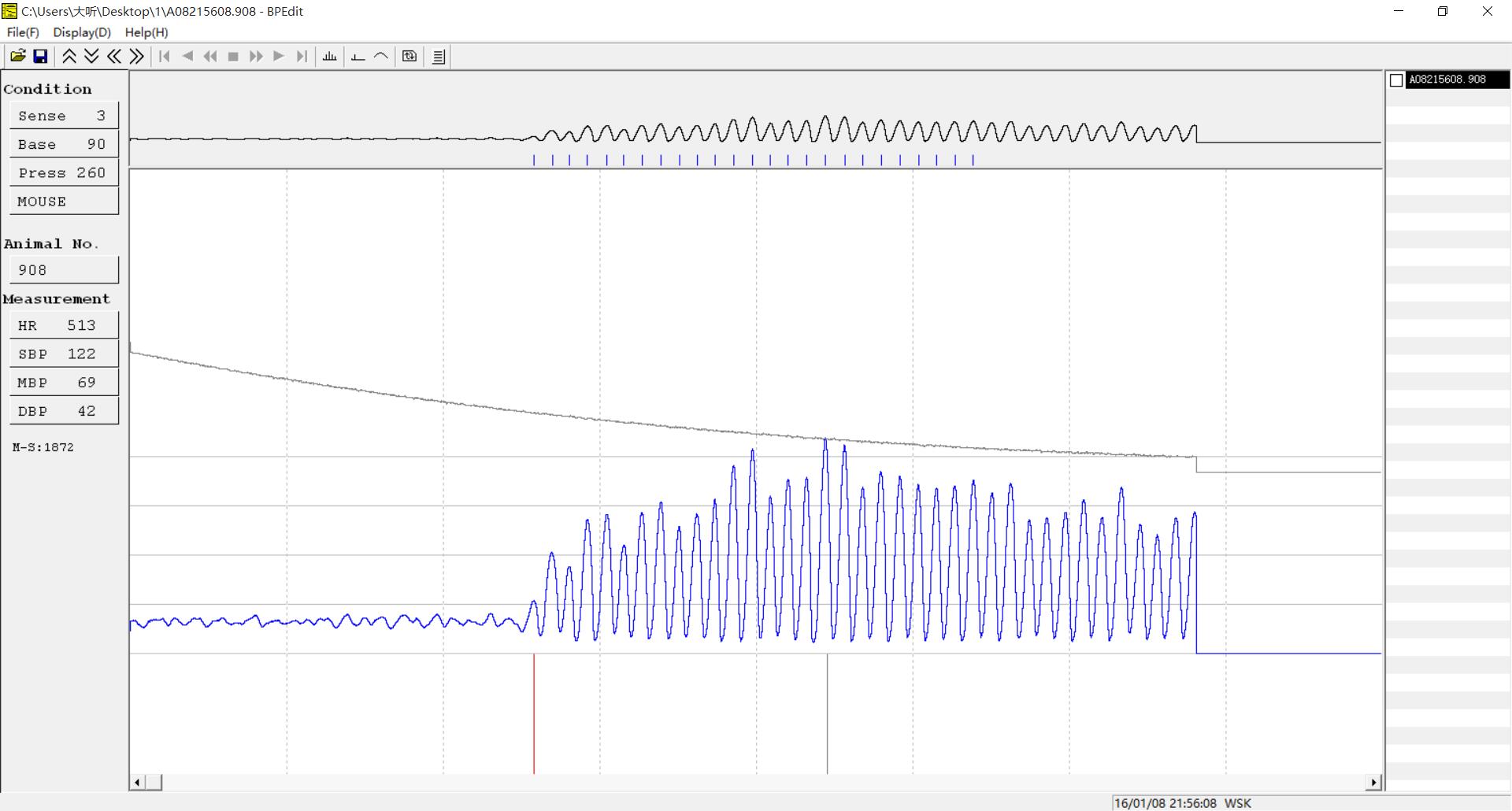

Supplement: S1 File — Pressure data of Ang II-induced AAA model and individual data points corresponding to each statistical graph. (ZIP) [file pone.0174821.s009.zip › Supplyment Data/Ang II model pressure/Image of pressure/S1-3.jpg]

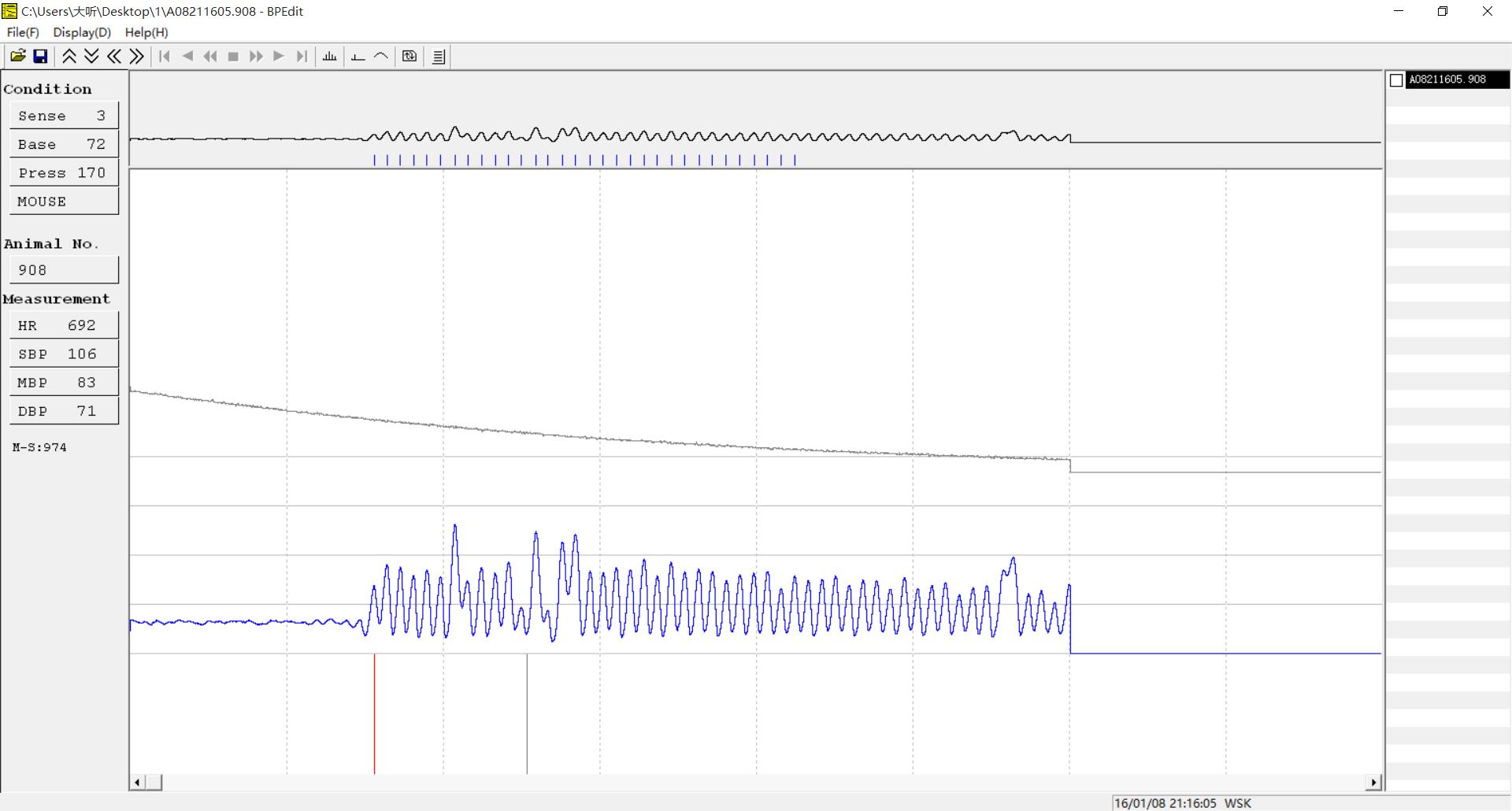

Supplement: S1 File — Pressure data of Ang II-induced AAA model and individual data points corresponding to each statistical graph. (ZIP) [file pone.0174821.s009.zip › Supplyment Data/Ang II model pressure/Image of pressure/S2-1.jpg]

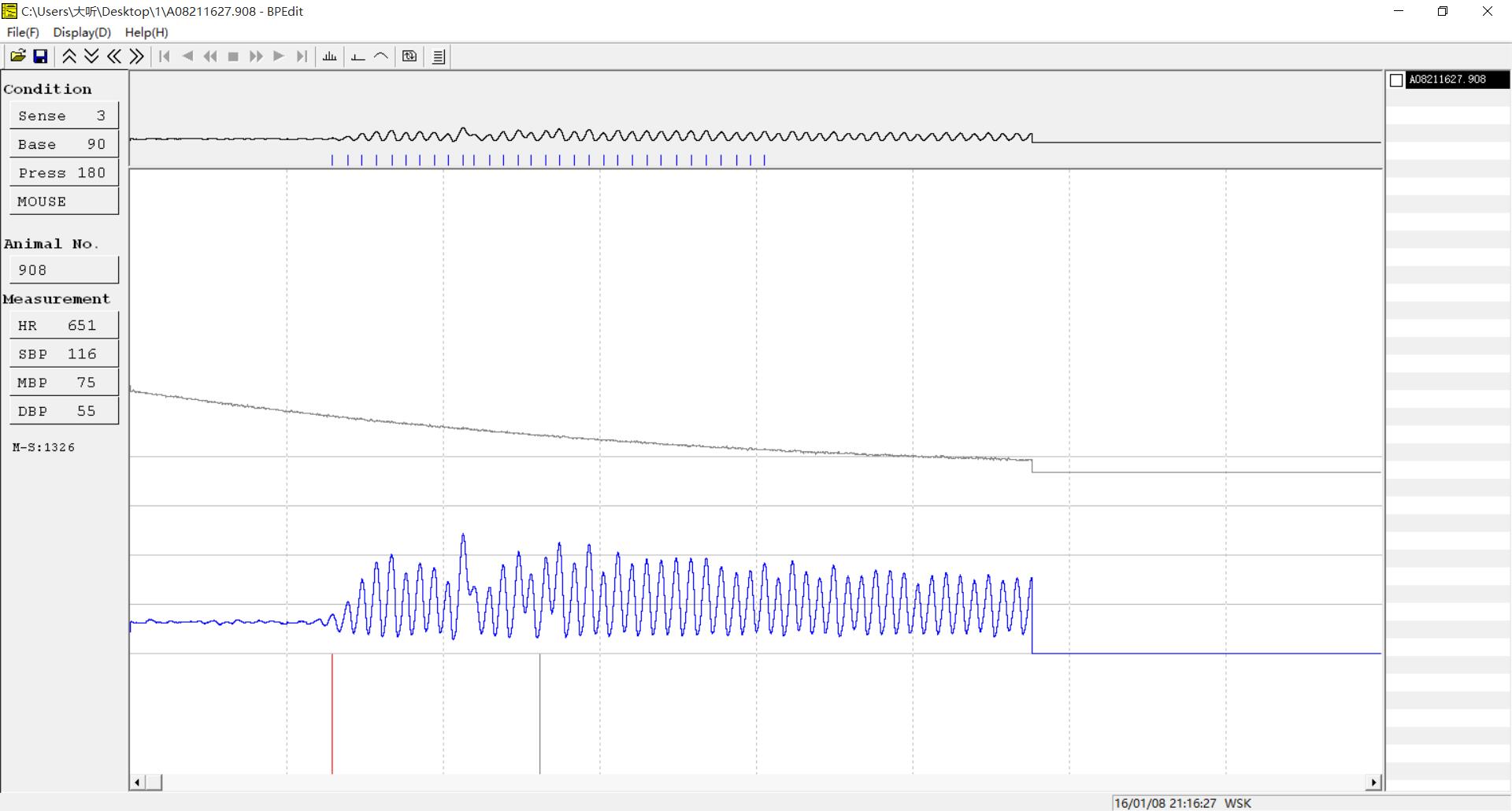

Supplement: S1 File — Pressure data of Ang II-induced AAA model and individual data points corresponding to each statistical graph. (ZIP) [file pone.0174821.s009.zip › Supplyment Data/Ang II model pressure/Image of pressure/S2-2.jpg]

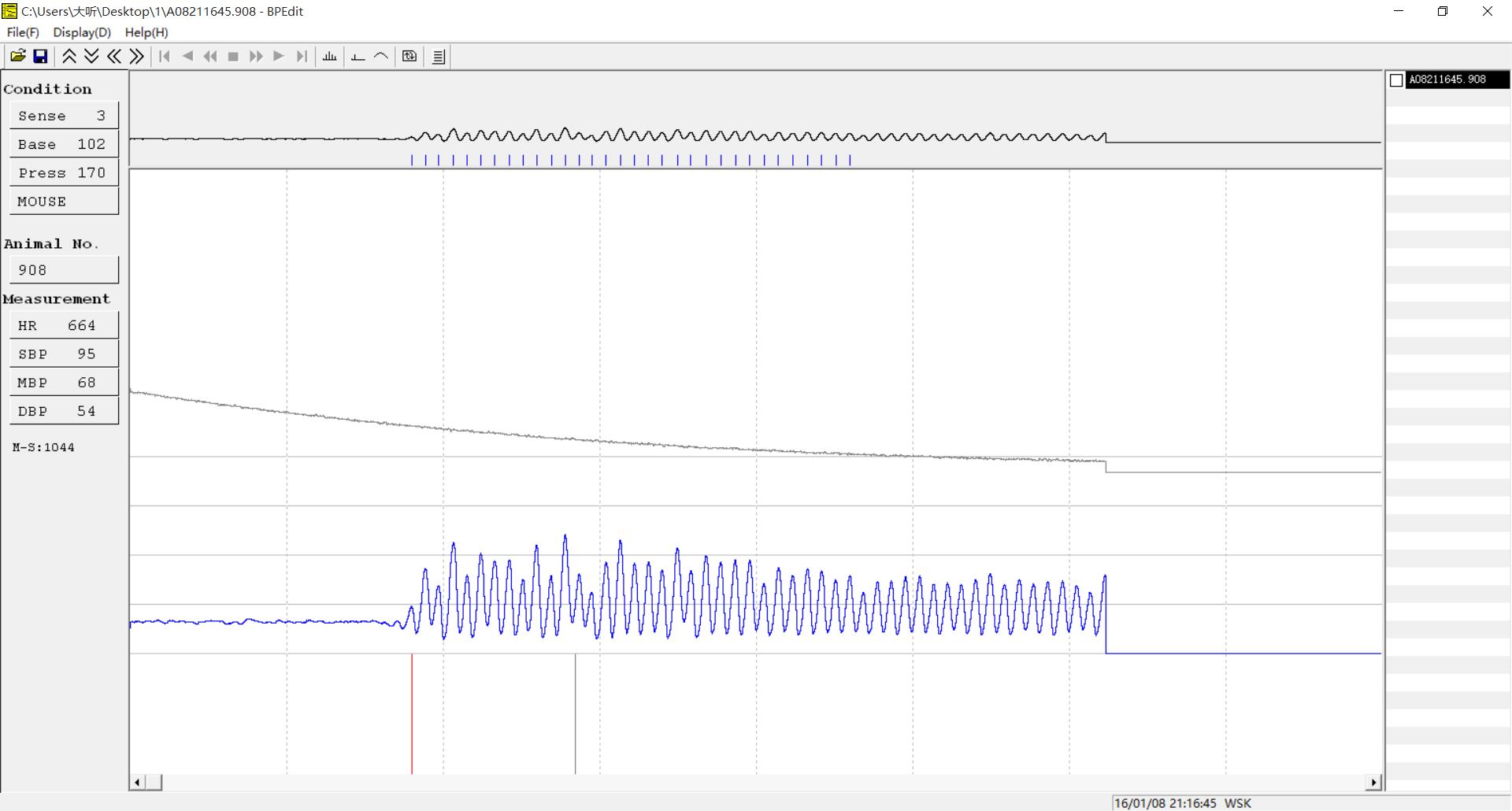

Supplement: S1 File — Pressure data of Ang II-induced AAA model and individual data points corresponding to each statistical graph. (ZIP) [file pone.0174821.s009.zip › Supplyment Data/Ang II model pressure/Image of pressure/S2-3.jpg]

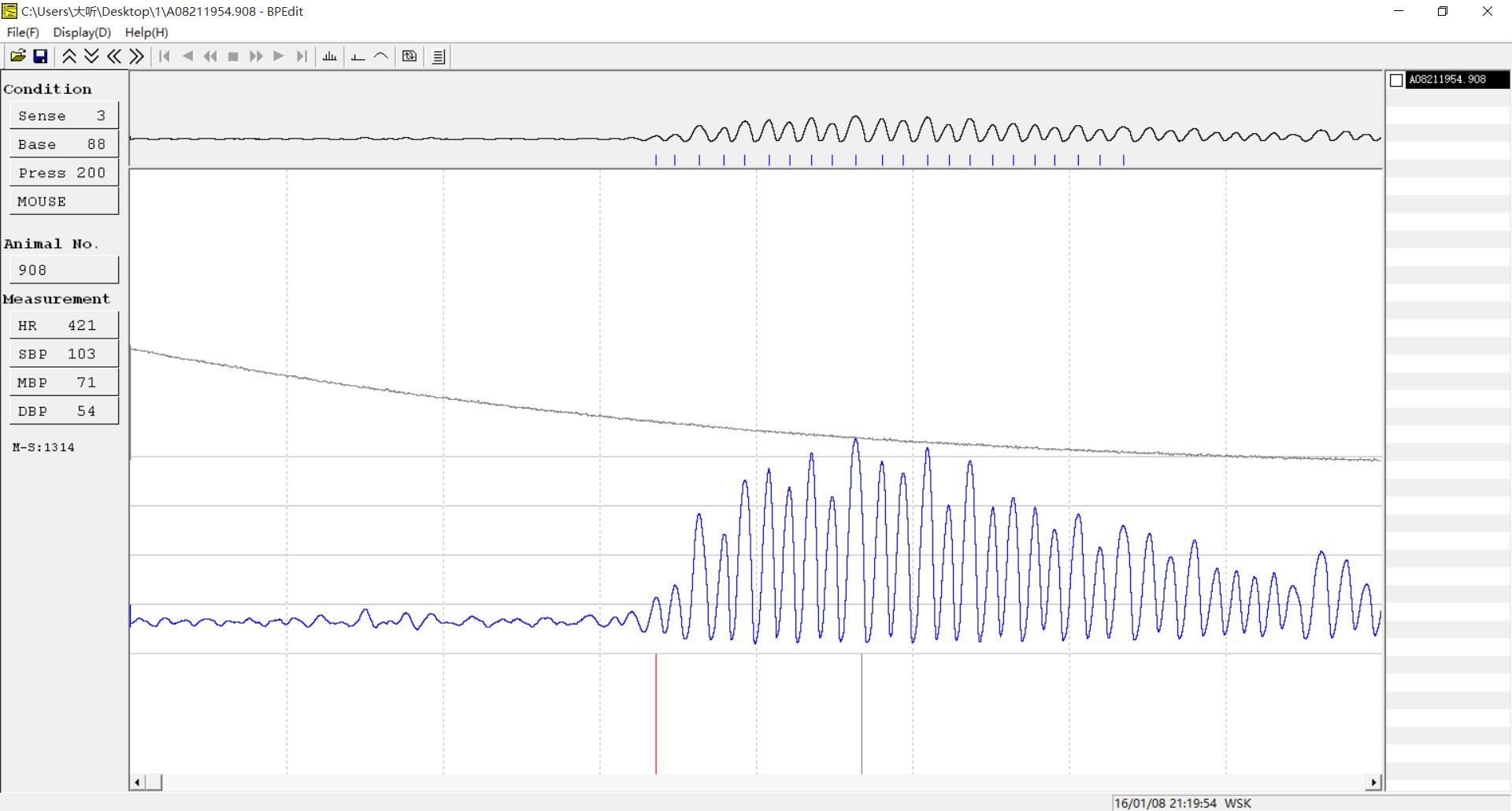

Supplement: S1 File — Pressure data of Ang II-induced AAA model and individual data points corresponding to each statistical graph. (ZIP) [file pone.0174821.s009.zip › Supplyment Data/Ang II model pressure/Image of pressure/S4-1.jpg]

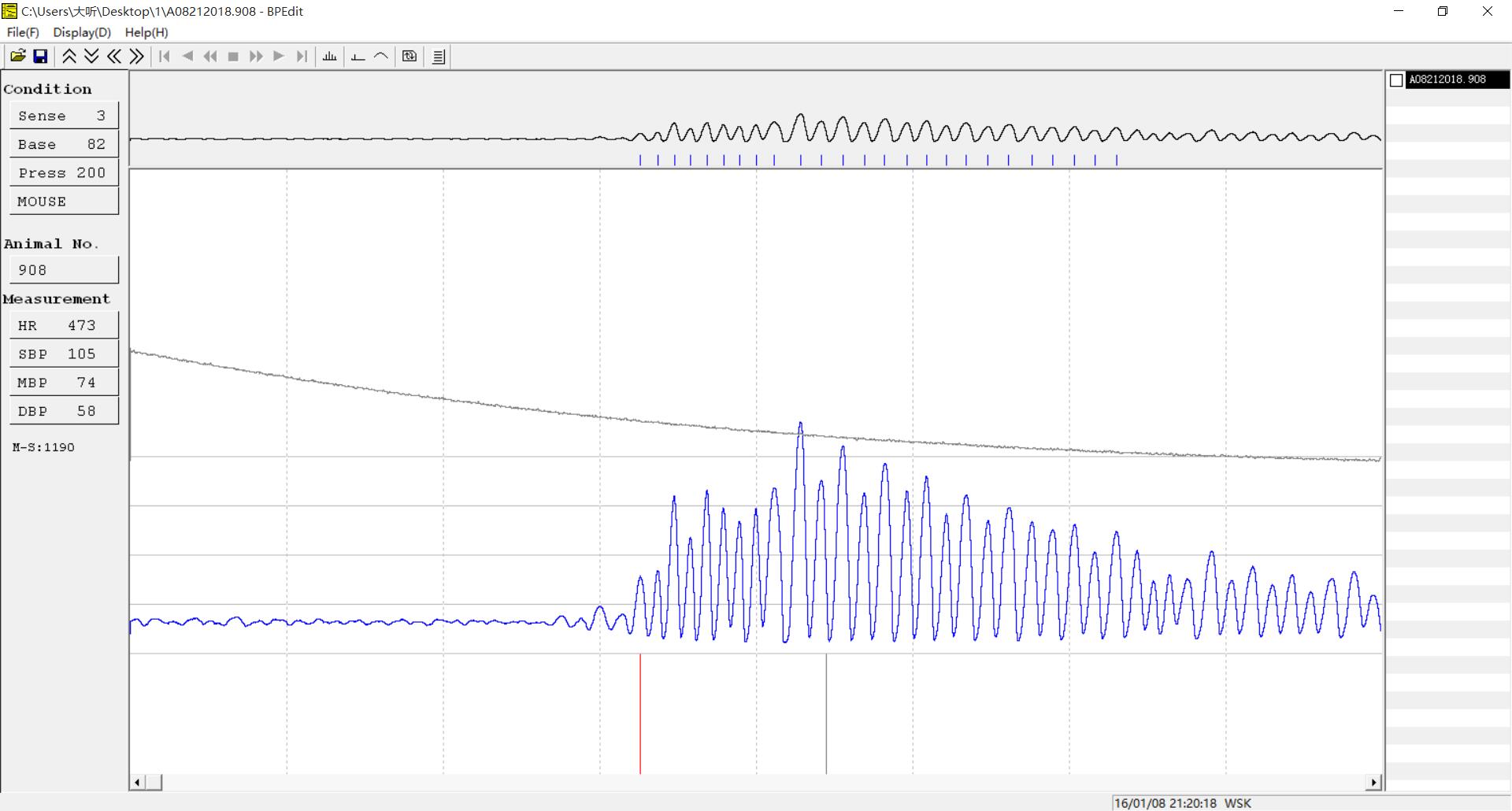

Supplement: S1 File — Pressure data of Ang II-induced AAA model and individual data points corresponding to each statistical graph. (ZIP) [file pone.0174821.s009.zip › Supplyment Data/Ang II model pressure/Image of pressure/S4-2.jpg]

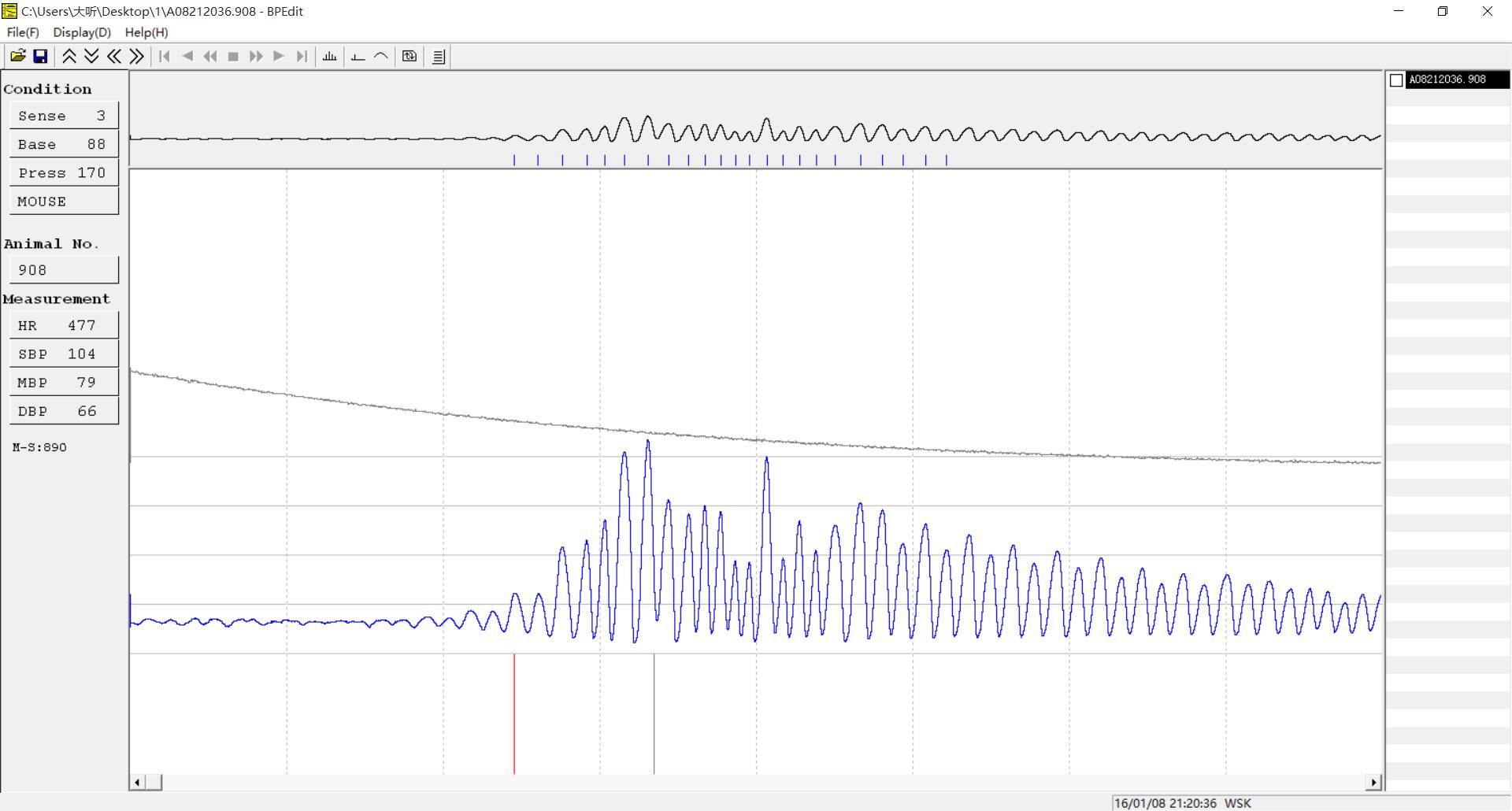

Supplement: S1 File — Pressure data of Ang II-induced AAA model and individual data points corresponding to each statistical graph. (ZIP) [file pone.0174821.s009.zip › Supplyment Data/Ang II model pressure/Image of pressure/S4-3.jpg]

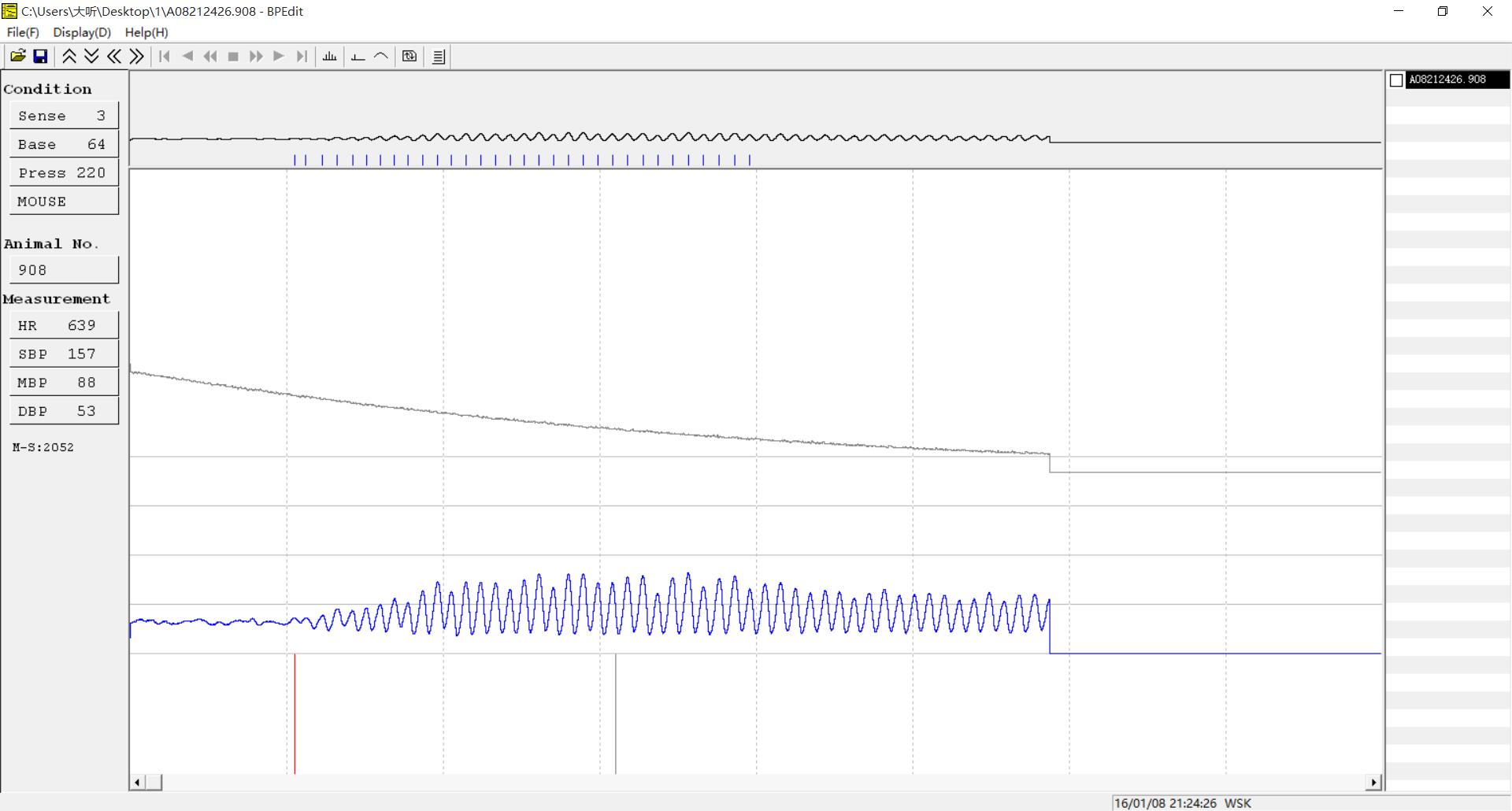

Supplement: S1 File — Pressure data of Ang II-induced AAA model and individual data points corresponding to each statistical graph. (ZIP) [file pone.0174821.s009.zip › Supplyment Data/Ang II model pressure/Image of pressure/S5-1.jpg]

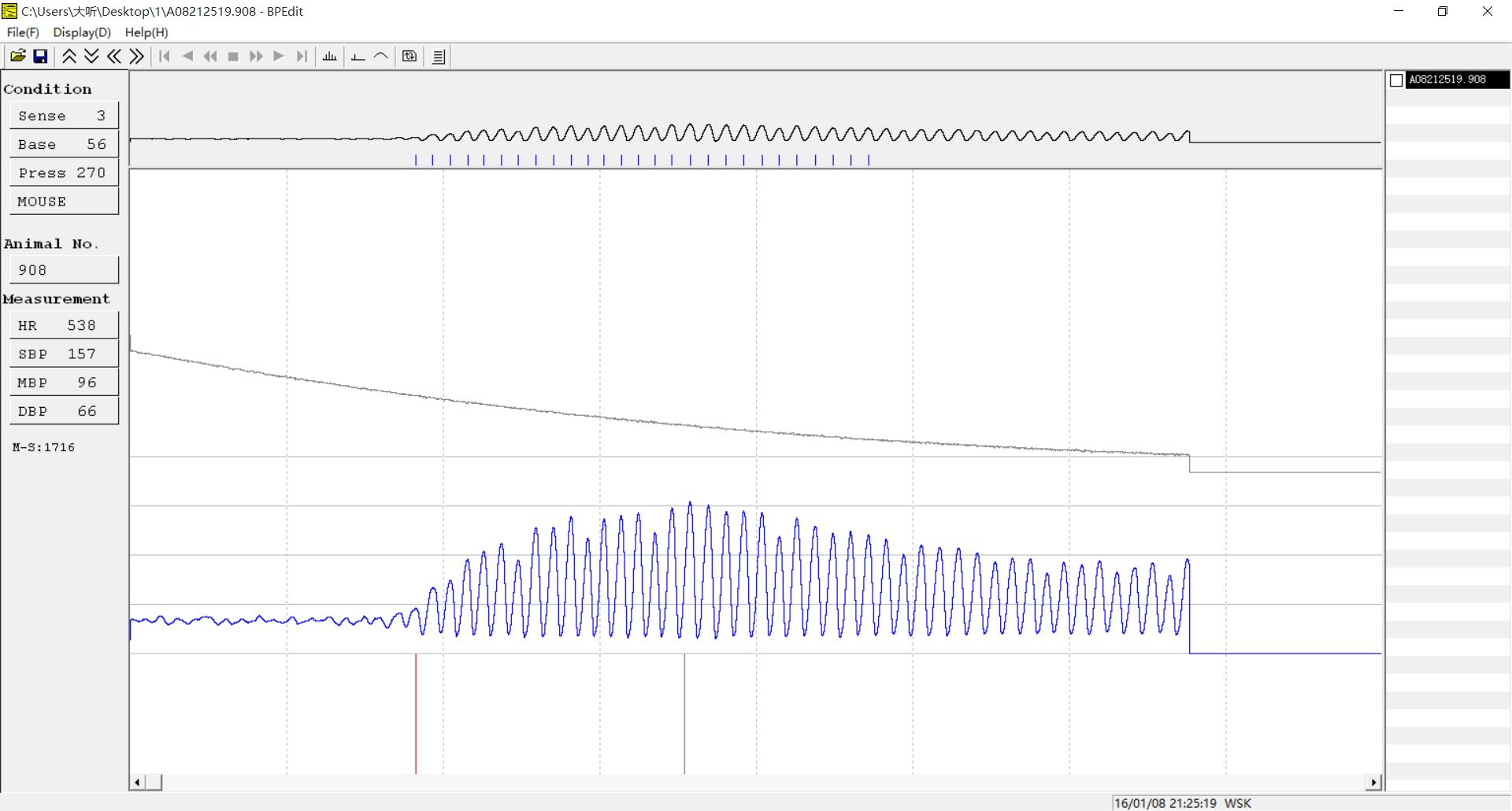

Supplement: S1 File — Pressure data of Ang II-induced AAA model and individual data points corresponding to each statistical graph. (ZIP) [file pone.0174821.s009.zip › Supplyment Data/Ang II model pressure/Image of pressure/S5-2.jpg]

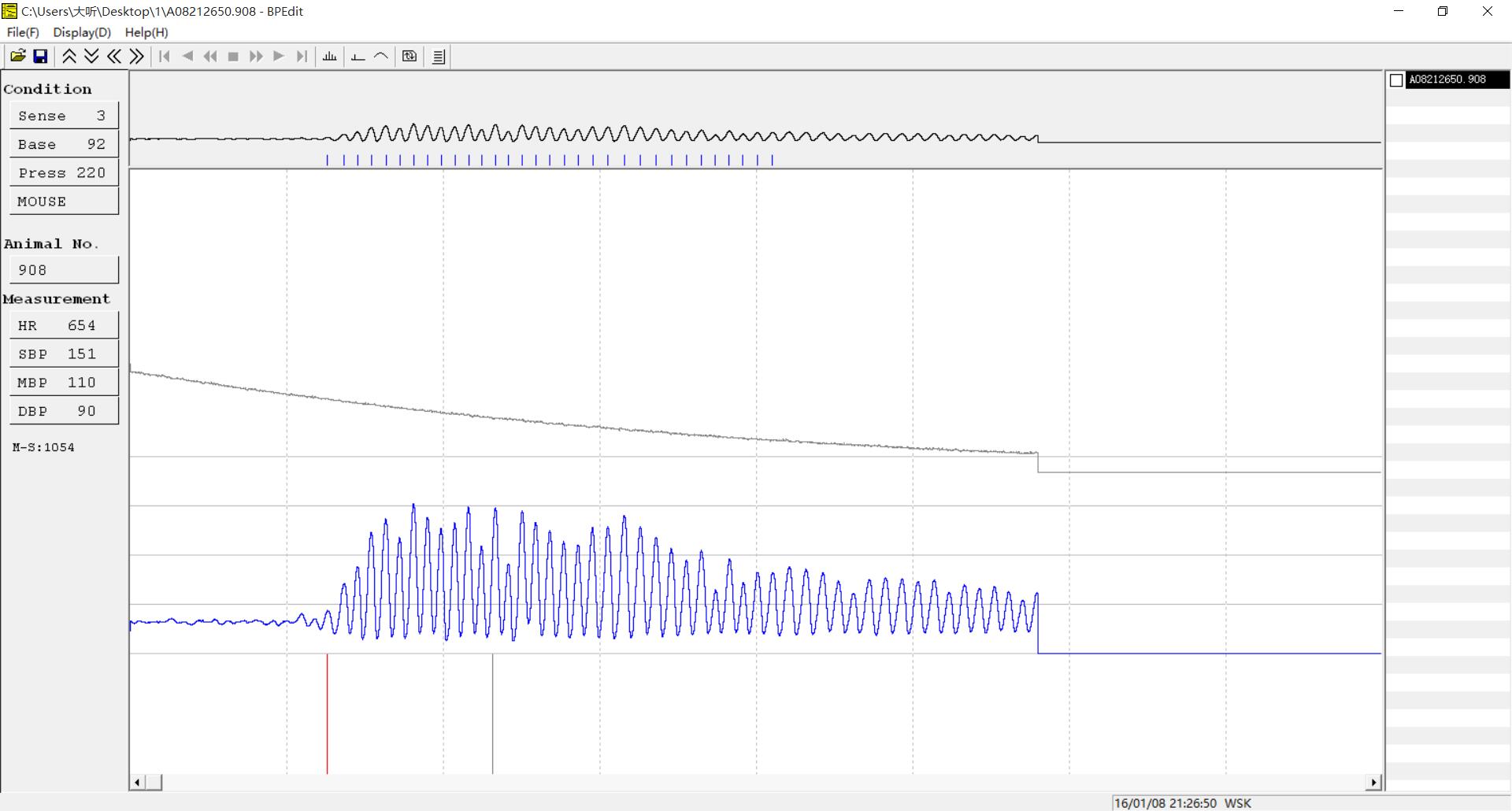

Supplement: S1 File — Pressure data of Ang II-induced AAA model and individual data points corresponding to each statistical graph. (ZIP) [file pone.0174821.s009.zip › Supplyment Data/Ang II model pressure/Image of pressure/S5-3.jpg]

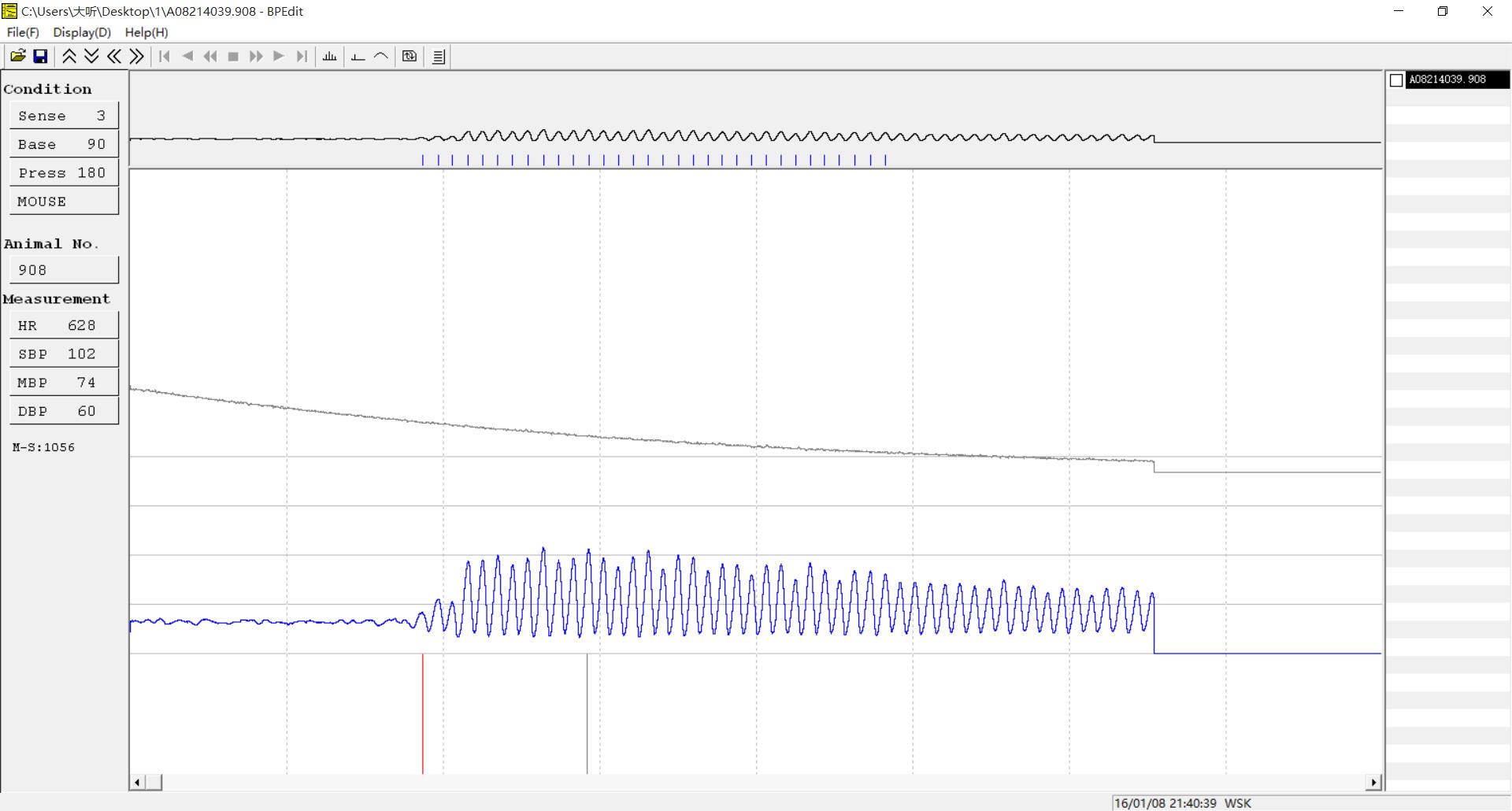

Supplement: S1 File — Pressure data of Ang II-induced AAA model and individual data points corresponding to each statistical graph. (ZIP) [file pone.0174821.s009.zip › Supplyment Data/Ang II model pressure/Image of pressure/S6-1.jpg]

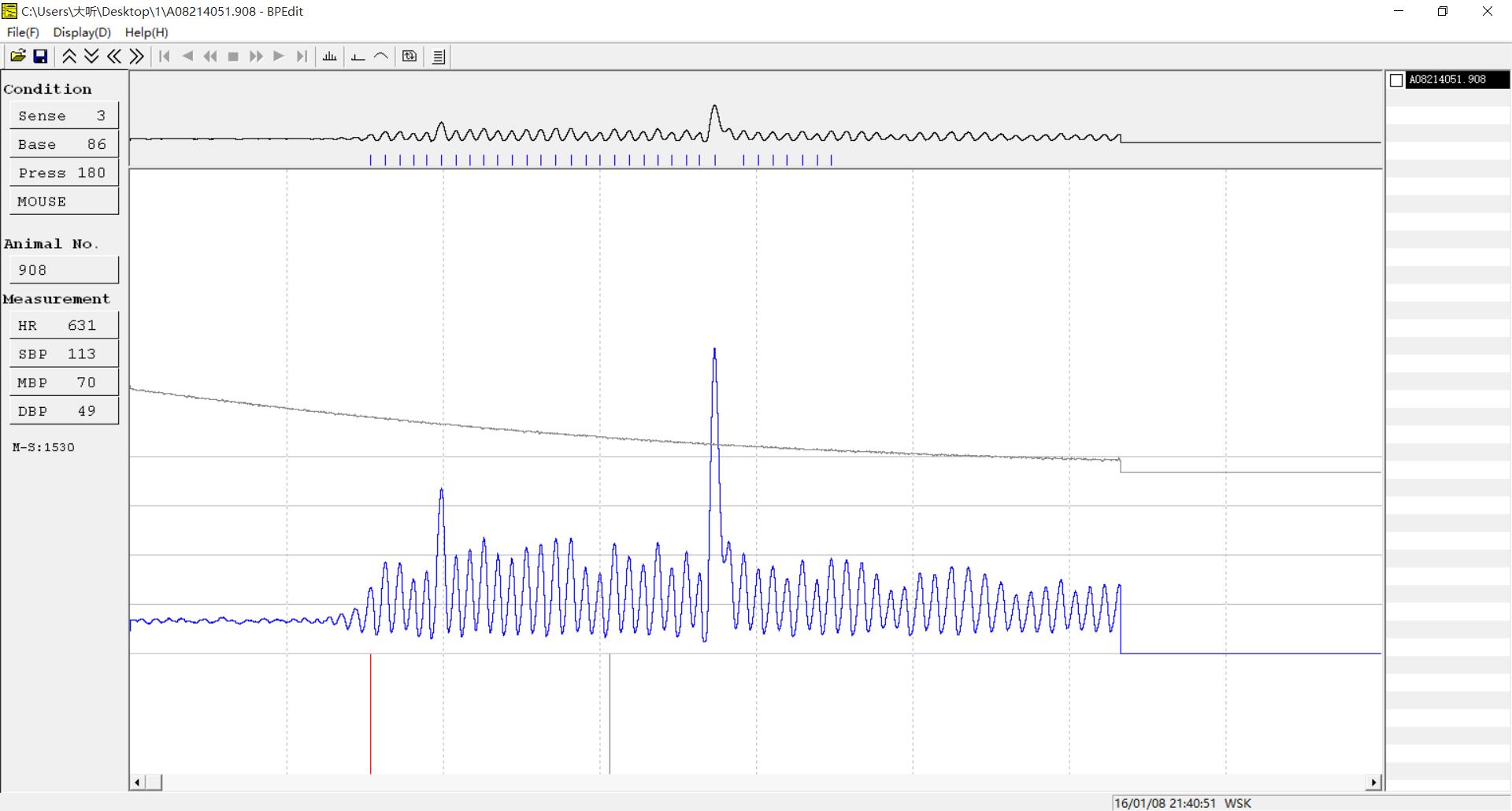

Supplement: S1 File — Pressure data of Ang II-induced AAA model and individual data points corresponding to each statistical graph. (ZIP) [file pone.0174821.s009.zip › Supplyment Data/Ang II model pressure/Image of pressure/S6-2.jpg]

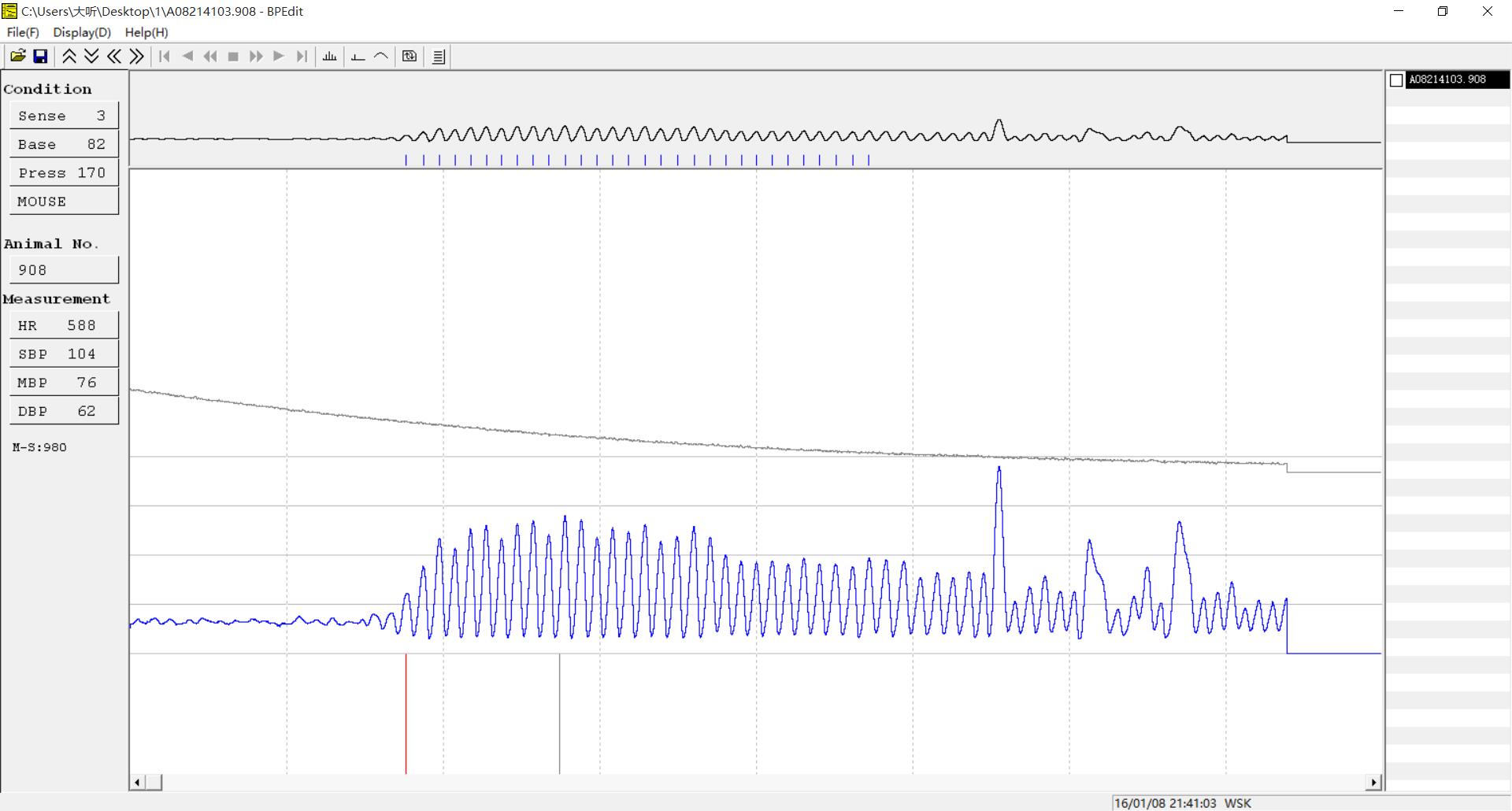

Supplement: S1 File — Pressure data of Ang II-induced AAA model and individual data points corresponding to each statistical graph. (ZIP) [file pone.0174821.s009.zip › Supplyment Data/Ang II model pressure/Image of pressure/S6-3.jpg]

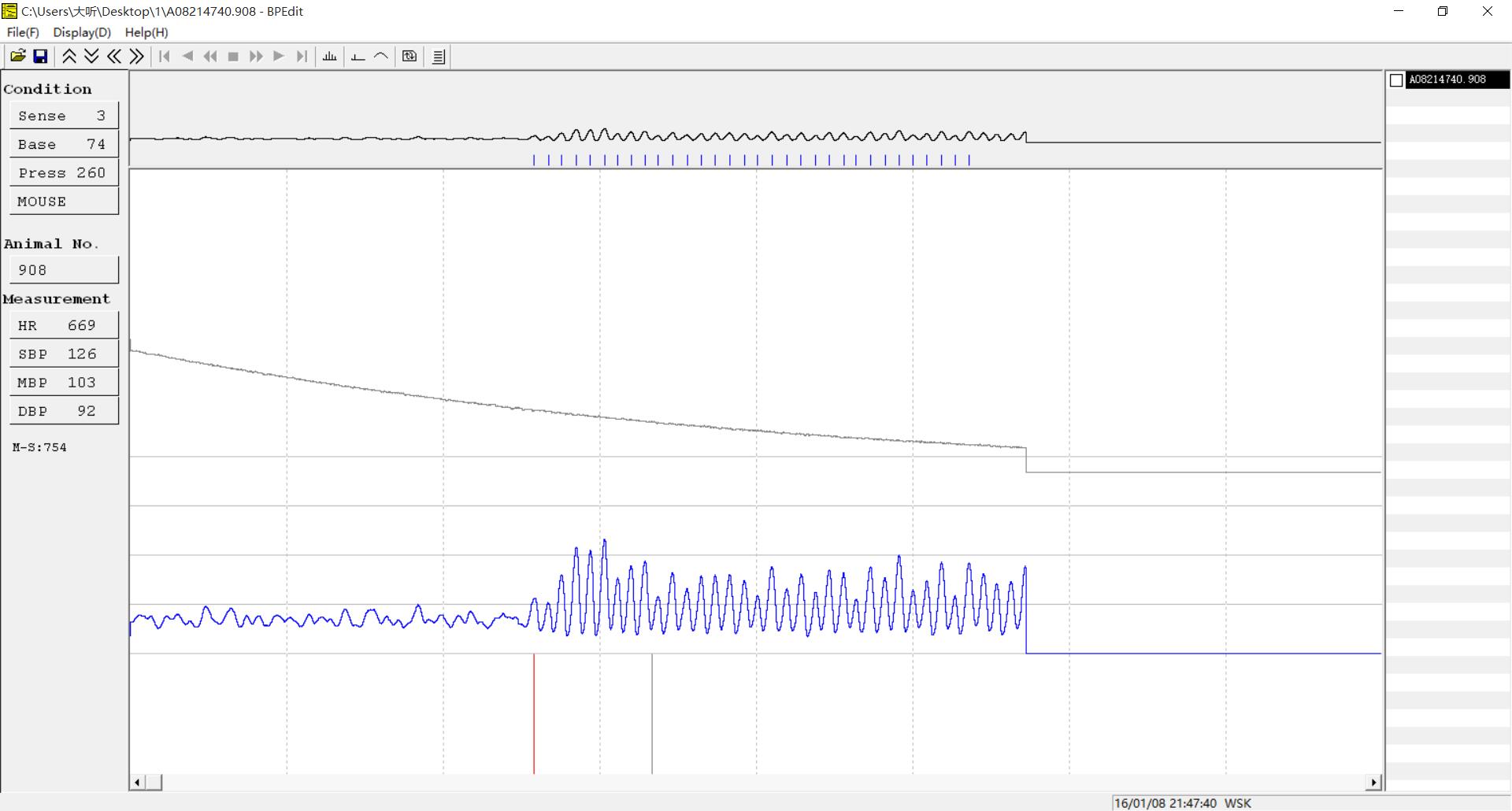

Supplement: S1 File — Pressure data of Ang II-induced AAA model and individual data points corresponding to each statistical graph. (ZIP) [file pone.0174821.s009.zip › Supplyment Data/Ang II model pressure/Image of pressure/S7-1.jpg]

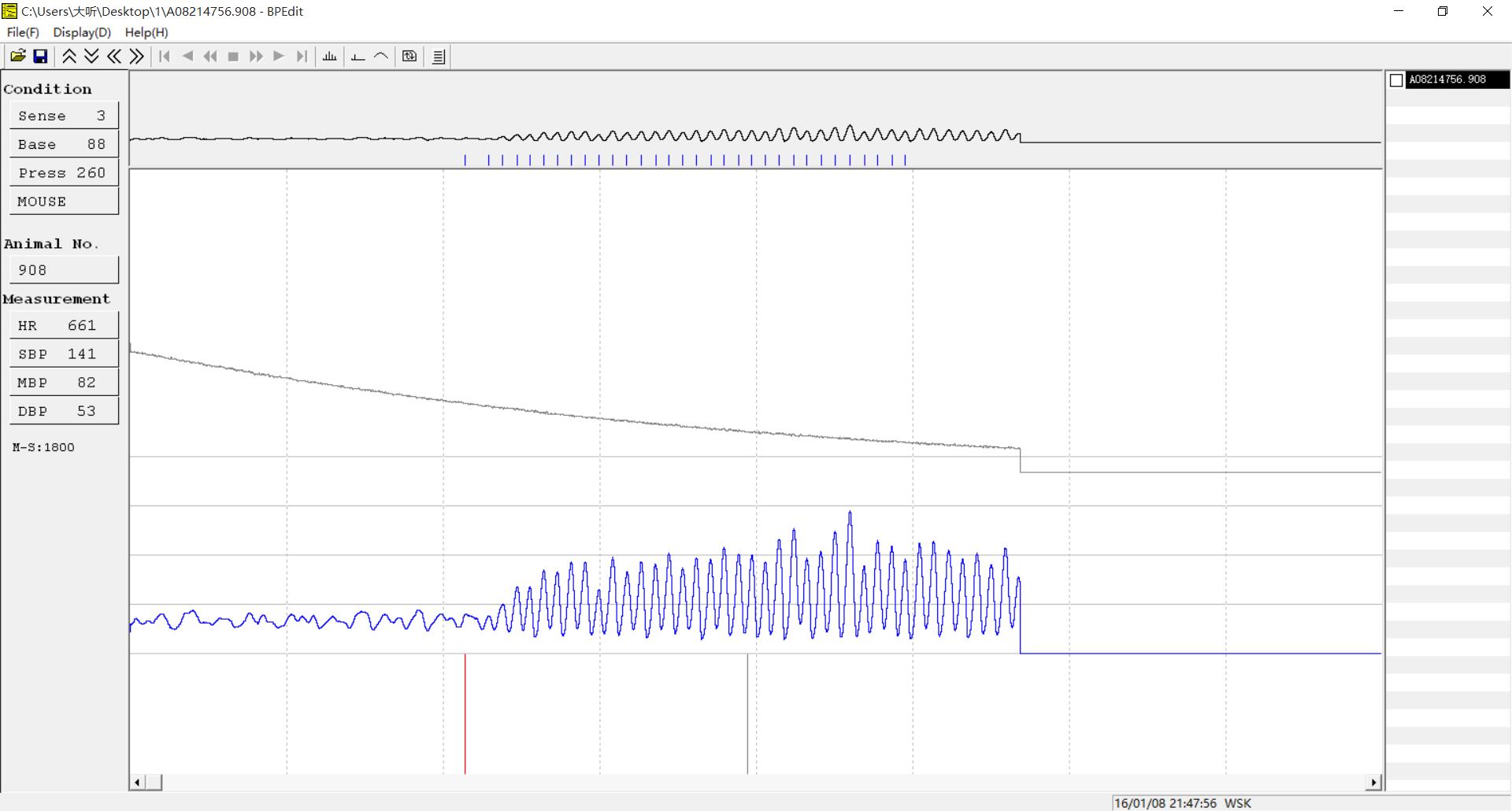

Supplement: S1 File — Pressure data of Ang II-induced AAA model and individual data points corresponding to each statistical graph. (ZIP) [file pone.0174821.s009.zip › Supplyment Data/Ang II model pressure/Image of pressure/S7-2.jpg]

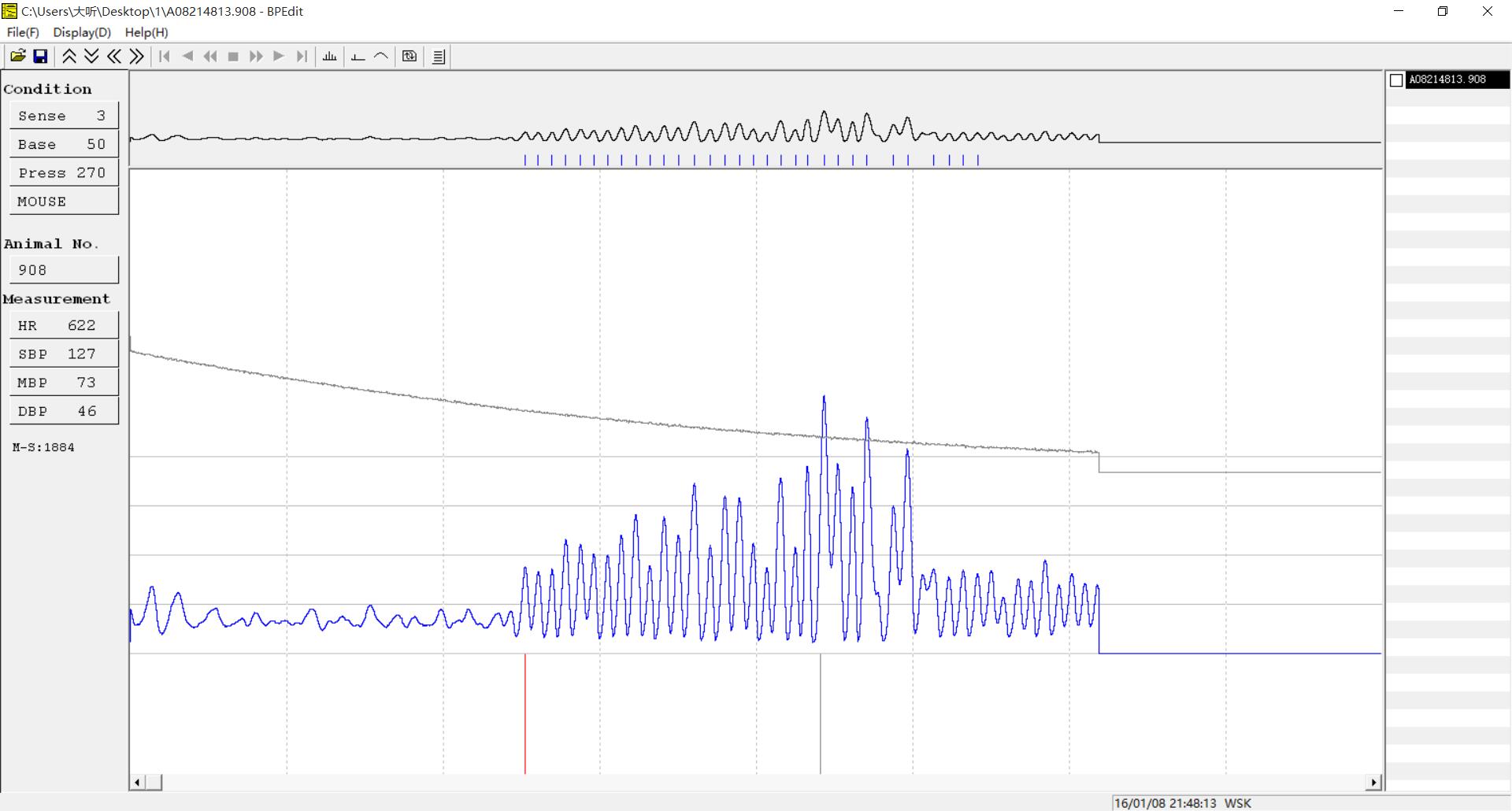

Supplement: S1 File — Pressure data of Ang II-induced AAA model and individual data points corresponding to each statistical graph. (ZIP) [file pone.0174821.s009.zip › Supplyment Data/Ang II model pressure/Image of pressure/S7-3.jpg]

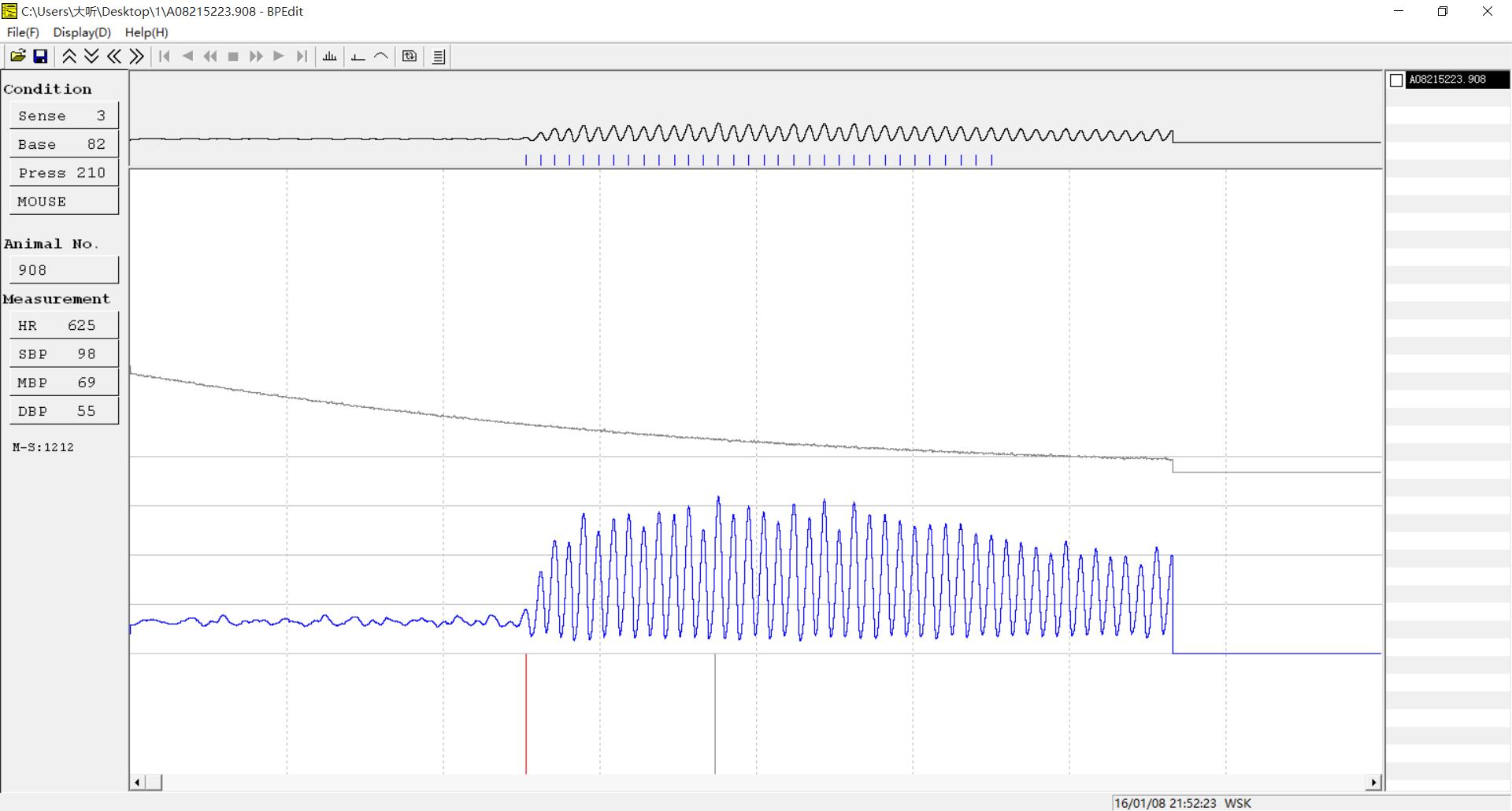

Supplement: S1 File — Pressure data of Ang II-induced AAA model and individual data points corresponding to each statistical graph. (ZIP) [file pone.0174821.s009.zip › Supplyment Data/Ang II model pressure/Image of pressure/S8-1.jpg]

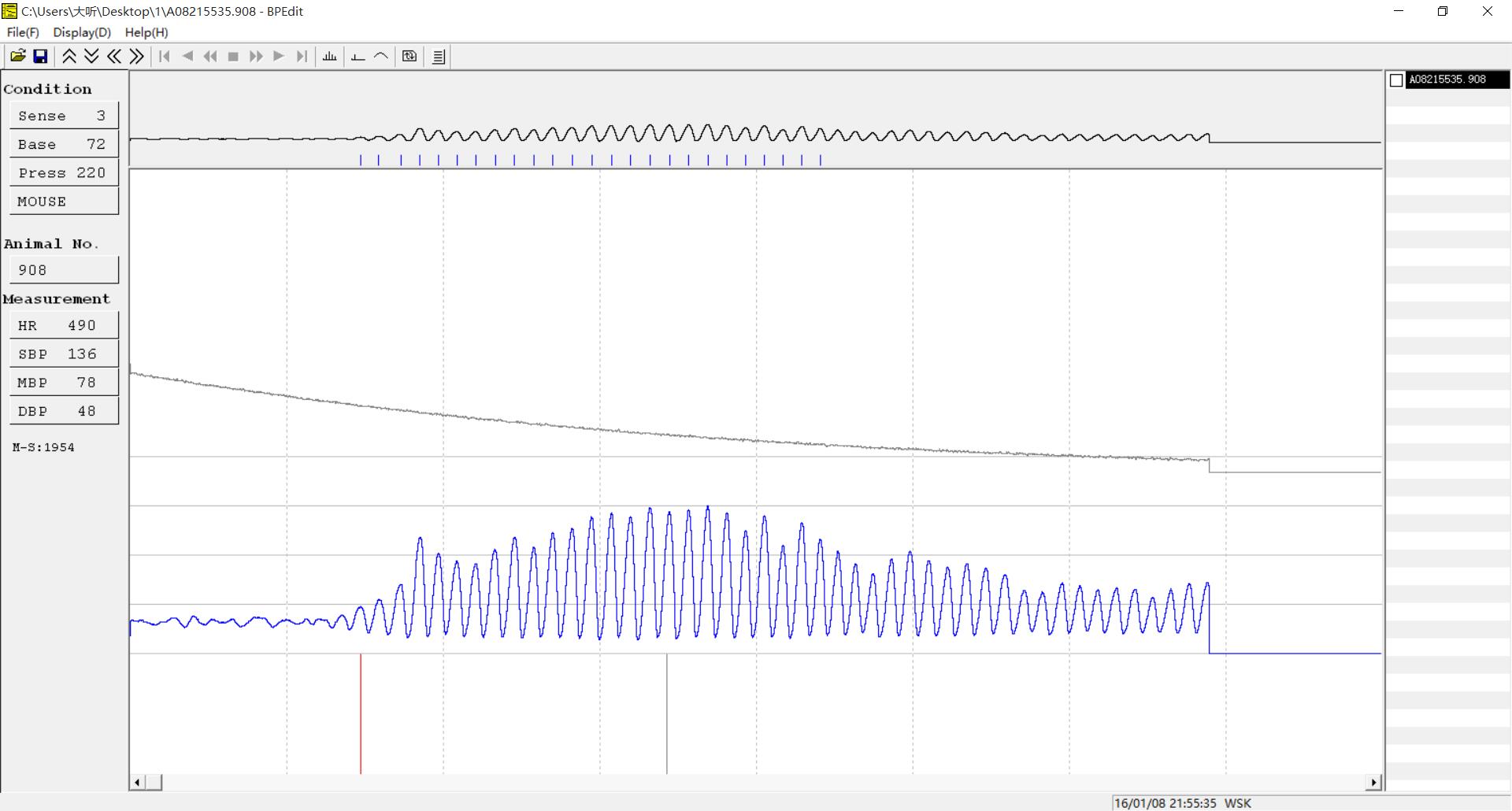

Supplement: S1 File — Pressure data of Ang II-induced AAA model and individual data points corresponding to each statistical graph. (ZIP) [file pone.0174821.s009.zip › Supplyment Data/Ang II model pressure/Image of pressure/S8-2-2.jpg]

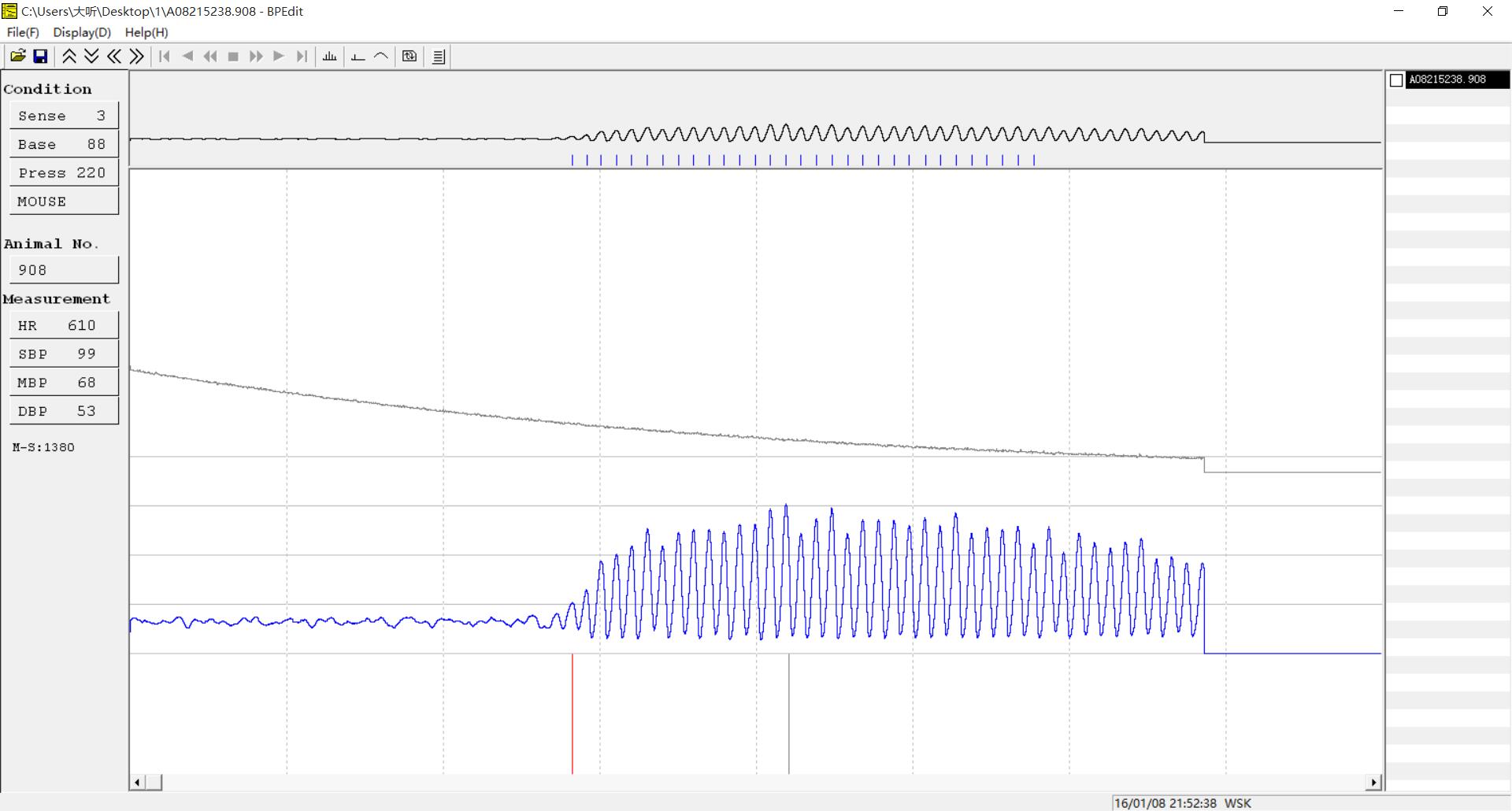

Supplement: S1 File — Pressure data of Ang II-induced AAA model and individual data points corresponding to each statistical graph. (ZIP) [file pone.0174821.s009.zip › Supplyment Data/Ang II model pressure/Image of pressure/S8-2.jpg]

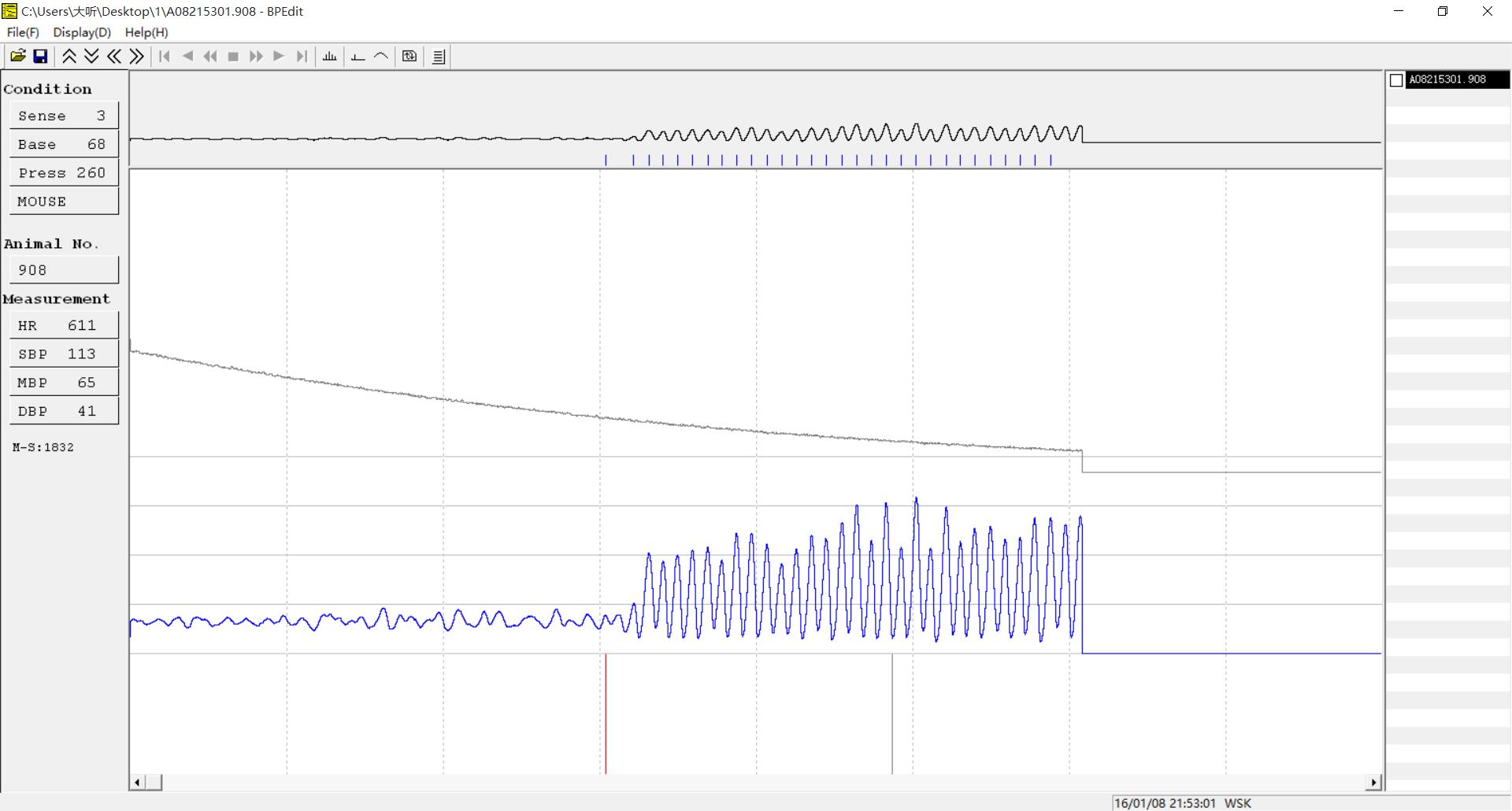

Supplement: S1 File — Pressure data of Ang II-induced AAA model and individual data points corresponding to each statistical graph. (ZIP) [file pone.0174821.s009.zip › Supplyment Data/Ang II model pressure/Image of pressure/S8-3.jpg]
